# CLK2 Expression Is Associated with the Progression of Colorectal Cancer and Is a Prognostic Biomarker

**DOI:** 10.1155/2022/7250127

**Published:** 2022-07-07

**Authors:** Jiarui Lin, Guixing Lin, Binbin Chen, Jinpeng Yuan, Yezhong Zhuang

**Affiliations:** Department of Gastrointestinal surgery, Cancer Hospital of Shantou University Medical College, Shantou, China

## Abstract

**Background:**

CLK2 is a splicing regulator and expressed ubiquitously in various malignancies. The study is aimed at exploring the potential roles of CLK2 in the development of colorectal cancer (CRC).

**Methods:**

Real-time PCR and analyses of The Cancer Genome Atlas (TCGA) and Human Protein Atlas (HPA) database were utilized to evaluate the CLK2 gene transcription level and protein level of colorectal cancer (CRC) tissue. The chi-squared and logistic regression tests were used to evaluate the relationship between CLK2 and clinicopathologic features. Kaplan-Meier survival curve and Cox regression analysis were performed to explore the prognostic significance of CLK2. The association between CLK2 expression and immune landscapes was explored by CIBERSORT and ESTIMATE. Furthermore, GSEA (Gene Set Enrichment Analysis) and alternative splicing (AS) analyses were performed to investigate the relationship between CLK2 expression and downstream signaling pathway.

**Results:**

The CLK2 expression was upregulated in CRC in both transcript and protein level. The elevated expression of CLK2 was correlated with local invasion and poor prognosis. Furthermore, CLK2 induced tumor cell adhesion and thereby promotes local invasion of CRC. The CLK2 expression significantly inhibited plasma cells and eosinophil infiltration and showed no relationship with immune and stromal scores of CRC samples. CLK2 might involve in Notch signaling pathway by regulating the AS of CTBP1.

**Conclusions:**

CLK2 might be a potential prognostic biomarker and therapeutic target for colorectal cancer.

## 1. Introduction

Colorectal cancer (CRC) is a common malignant tumor in the world, and its incidence is increasing year by year in China [1]. Surgery, radiotherapy, and chemotherapy are the main treatments for CRC [2, 3]. Despite the great improvement in the diagnosis and treatment of CRC, many patients die due to local recurrence and distant metastasis [4]. Therefore, it is essential to explore the potential mechanisms of CRC development and identify the potential biomarkers to improve the prognosis of CRC.

CLK2 is a protein kinase that phosphorylates splice factors and plays an essential role in regulating alternative splicing (AS) [5]. CLK2 is expressed ubiquitously, and increasing evidence has found that CLK2 is closely associated with various diseases, such as neurodegenerative diseases and cancers [6]. The abnormal expression of CLK2 contributes to misregulate the AS of tau gene, which is closely associated with sporadic Alzheimer's disease [7]. CLK2 acts as an oncogene and promotes cell invasion and migration via AS of genes in EMT pathways in breast cancer [8]. The phosphorylation function of CLK2 leads to the conformational switching of PAGE4, which affects the treatment response of androgen receptor antagonists [9]. In glioblastoma, the CLK2 expression is elevated and correlated with poor survival. Furthermore, CLK2 regulates the cell cycle of glioblastoma cells via FOXO3/p27 signaling pathway [10]. As a result, CLK2 inhibitors are developed in breast cancer and even other cancers [11–13]. A previous study has demonstrated that CLK2 inhibitor T-025 has antitumor efficacy in many tumors, especially in MYC-driven cancers [14]. A recent research indicated that CLK inhibitor SM08502 plays important roles in antitumor activities via regulation of Wnt pathway in gastrointestinal tumor models [15]. However, few studies have been done to explore the molecular mechanisms and prognostic significance of CLK2 in CRC.

In the present study, we aimed to explore the clinical and prognostic significance of the CLK2 expression. Also, the relationship between CLK2 expression and immune landscapes was evaluated. Finally, we further explore the potential molecular mechanisms of CLK2 in the development and tumorigenesis of CRC.

## 2. Materials and Methods

### 2.1. Data Mining

We downloaded the data of CLK2 and the corresponding clinical features from the colon adenocarcinoma (COAD) and rectum adenocarcinoma (READ) projects of The Cancer Genome Atlas (TCGA) database. The expression profiles with corresponding clinical data were used for evaluation of the clinical and prognostic significance of CLK2. Additionally, the high-throughput sequencing data of CRC was used to calculate the Percent-Spliced-In (PSI) value for each AS event by utilizing SpliceSeq software [16, 17]. The immunohistochemistry results of CLK2 in colorectal normal tissue and colorectal cancer tissue were downloaded from the Human Protein Atlas database.

A total of 50 fresh colorectal tissue samples, comprising 50 cancer samples and 50 adjacent nontumor samples, were obtained from the First Affiliated Hospital of Shantou University Medical college from 2020 to 2021. All specimens were frozen immediately after surgery and stored at −80°C. This study was approved by the Institutional Research Ethics Committee of the First Affiliated Hospital of Shantou University Medical College. All patients who participated in this study signed a written, informed consent before surgery.

### 2.2. Bioinformatics Analysis

The unpaired and paired Mann–Whitney *U* test was used to evaluate the differential expression of CLK2 between normal samples and CRC samples. To explore the relationship between CLK2 expression and the corresponding clinical features, the chi-squared and logistic regression tests were applied. The expression level of CLK2 was divided into two groups (high expression group and low expression group) according to its median expression. Kaplan–Meier curve, univariate, and multivariate Cox regression analysis was performed to evaluate the prognostic value of CLK2.

### 2.3. The Association between CLK2 Expression and Immune Landscapes

ESTIMATE and CIBERSORT are two different bioinformatic algorithms to evaluate the immune landscapes of tumor samples from gene expression profile [18–20]. ESTIMATE *R* package was used to calculate the stromal and immune scores, and the Wilcoxon test was performed to explore the relationship between stromal and immune scores and CLK2 expression level. CIBERSORT *R* package was used to evaluate the relative abundance of 22 immune cells, and each sample will obtain a *P* value. Samples with *P* value <0.05 will be used to explore the association between CLK2 expression level and 22 immune cells.

### 2.4. Exploration of Signaling Pathways That CLK2 Involved in CRC

GSEA is a novel computational algorithm to explore whether a predetermined gene set show statistically significant differences between two different biological states [21]. Patients were categorized into two groups (high CLK2 expression and low CLK2 expression groups) based on the median expression of CLK2. KEGG signaling pathways enriched in the high CLK2 expression group was explored by GSEA 4.1 software with gene set c2 (cp. kegg.v.6.2.symbols.gmt). A NOM *P* value <0.05 and FDR *q* value <0.05 were considered significant difference.

### 2.5. Exploration the Relationship between CLK2 Expression and the Alternative Splicing of Genes

As mentioned before, CLK2 functions as splice factor and plays an essential role in AS. AS events can be divided into 7 types: alternate acceptor site (AA), alternate donor site (AD), alternate promoter (AP), alternate terminator (AT), exon skip (ES), mutually exclusive exons (ME), and retained intron (RI) [22, 23]. We performed Student's *t*-test to evaluate the differential AS event prevalence between normal and tumor samples [24]. Furthermore, the relationship between CLK2 expression and PSI value of differential splicing genes was explored by Spearman correlation analysis.

### 2.6. Quantitative Real-Time Polymerase Chain Reaction

Total RNA from CRC or adjacent tissue samples was extracted by using TRIzol Reagent or TRIzol LS Reagent. The cDNA was generated by using Geneseed® II First Strand cDNA Synthesis Kit. Complementary DNA primers specific for CLK2 amplification were as follows: forward, 5-AATATTTTTACCGGGGTCGC-3′; reverse, 5-AGCCGCTTAGCTGGTTCATA-3′. And the qPCR was performed in a 20 *μ*l reaction system according to the instructions. Ten-microliter 2xqPCR SYBR-Green 30 master mix (Vazyme Biotech), 0.4 *μ*L forward primer (10 *μ*M), 0.4 *μ*L reverse primer (10 *μ*M), and 5 *μ*L cDNA were included in the 20 *μ*L reaction system. All samples were tested in triplicate. Relative mRNA levels of CLK2 were normalized to the GAPDH expression.

## 3. Results

### 3.1. Evaluation of CLK2 Expression and Its Association with Clinical Features in CRC

**Figure 1:**
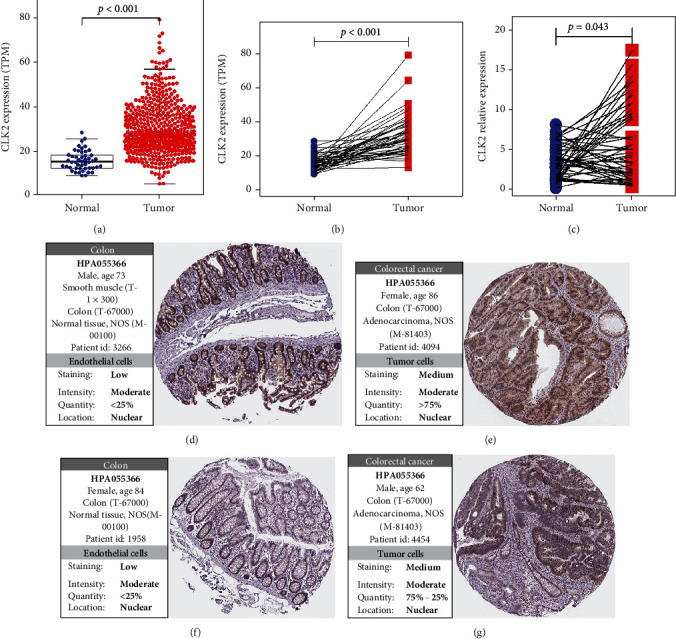
The CLK2 expression in colorectal cancer tissue and adjacent normal tissue. (a) The CLK2 expression in 48 normal colorectal samples and 554 colorectal cancer samples in TCGA cohort. (b) The CLK2 expression in 48 paired colorectal normal and cancer samples in TCGA cohort. (c) The CLK2 expression in 50 paired colorectal normal and cancer samples in our validation cohort. (d)–(g) The Human Protein Atlas database shows the immunohistochemical results of CLK2 in CRC tissue compared with noncancerous colon tissues.

A total of 602 samples (554 tumor samples and 48 normal samples) were included for differential expression analysis. Among them, a part of tumor samples match with normal samples. Hence, differential expression analyses of unpaired and paired samples were performed and validated in our CRC cohort. The results indicated that the CLK2 expression was elevated in CRC tissue compared to noncancerous tissue (Figures 1(a)–1(c)). Furthermore, we explored the protein expression of CLK2 from the HPA database, and the IHC staining results indicated that the protein expression of CLK2 was upregulated in CRC tissue compared to normal colon tissue (Figures 1(d)–1(g)). The elevated expression of CLK2 was closely associated with gender (P = 0.046), local invasion (P = 0.005), and TNM stage (P = 0.032). However, other clinical parameters, such as age, radiation therapy, chemotherapy, lymph node metastasis, and distant metastasis, showed no relationship with the CLK2 expression (Table 1). Univariate logistic regression analysis showed that the high expression of CLK2 was correlated with gender (female vs. male, OR = 0.707, P = 0.046), local invasion (T4 vs. T1, OR = 8.242, P = 0.002), and TNM stage (stage III vs. stage I, OR = 1.696, P = 0.044) (Table 2). Furthermore, in our validation cohort, the CLK2 expression was significantly correlated with local invasion (T4 vs. T1 + T2 + T3, OR = 6.729, P = 0.003). Other clinical parameters, such as age, gender, grade, tumor size, lymph node metastasis, and TNM stage, showed no correlation with the CLK2 expression.

### 3.2. Evaluation of the Prognostic Value of CLK2 in CRC

**Figure 2:**
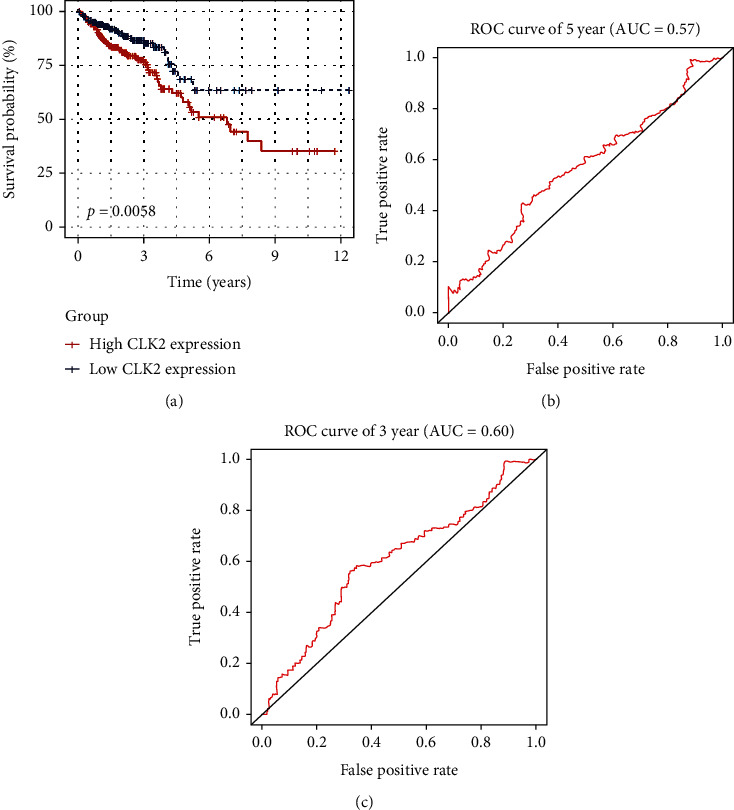
Kaplan-Meier survival analysis for high CLK2 expression group and low CLK2 expression group. ns: P > .05, not significant. ^∗^P < 0.05, ^∗∗^P < 0.01, ^∗∗∗^P < 0.001.

We performed Kaplan–Meier curve analysis, and the result indicated that the high expression of CLK2 correlated with poor overall survival (P = 0.0058, Figure 2(a)). The area under the ROC curve of 3 and 5 years was 0.60 and 0.57, respectively (Figures 2(b) and 2(c)). Then, a total of 480 patients were included for further Cox regression analysis after removal of invalid clinical parameters. The result of univariate Cox analysis shown that age (HR = 2.09, P = 0.002), TNM stage (HR = 3.76, P < 0.001), local invasion (HR = 3.88, P < 0.001), lymph metastasis (HR = 3.23, P < 0.001), distant metastasis (HR = 4.35, P < 0.001), and CLK2 expression (HR = 2.01, P = 0.002) were closely associated with unfavorable prognosis. Furthermore, these factors were included to perform multivariate Cox analysis, and the result demonstrated that CLK2 expression (HR = 1.71, P = 0.019), age (HR = 2.70, P < 0.001), TNM stage (HR = 1.33, P = 0.03), local invasion (HR = 2.30, P = 0.003), and distant metastasis (HR = 2.30, P = 0.002) were independent prognostic factors for CRC (Table 3).

### 3.3. The Association between CLK2 Expression and Immune Infiltration Level

**Figure 3:**
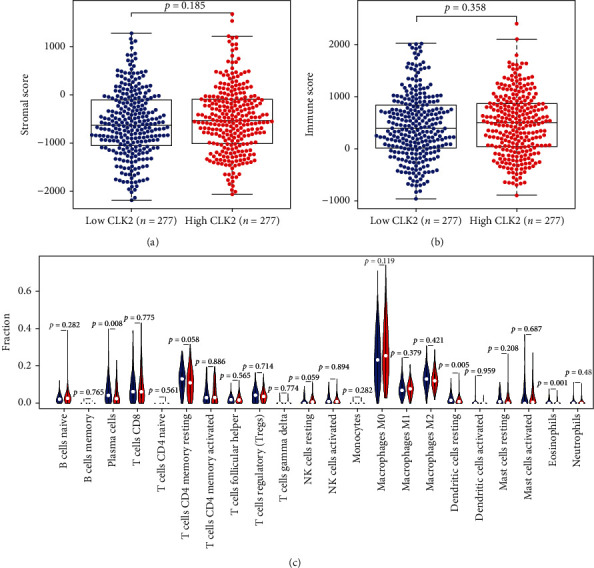
Analysis of relationship between CLK2 expression and immune landscapes. (a) Stromal scores between high and low CLK2 expression groups. (b) Immune scores between high and low CLK2 expression groups. (c) Violin plot of immune infiltration level between high and low CLK2 expression groups.

The ESTIMATE analysis indicated that the CLK2 expression showed no relationship with stromal score (P = 0.185, Figure 3(a)) and immune score (P = 0.358, Figure 3(b)). However, the CIBERSORT analysis revealed that the infiltration level of plasma cells and eosinophils was significantly higher in the high CLK2 expression group, and the infiltration level of dendritic cells resting was significantly higher in the low CLK2 expression group (Figure 3(c)).

### 3.4. Exploration of Signaling Pathways Enriched in High CLK2 Expression Group

**Figure 4:**
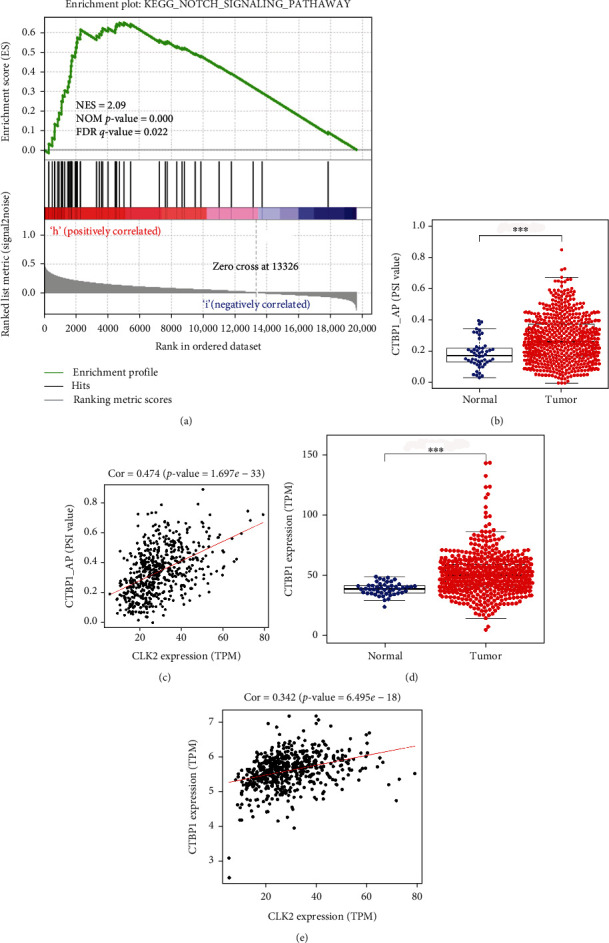
Analysis of signaling pathways enriched in the high CLK2 expression group. (a) Analysis of GSEA. (b) AP event of CTBP1 in colorectal cancer tissue and adjacent normal tissue. (c) Relationship between AP event of CTBP1 and CLK2 expression. (d) the CTBP1 expression in colorectal cancer tissue and adjacent normal tissue. (e) Relationship between CTBP1 and CLK2 in transcriptional level. ^∗∗∗^P < 0.001.

The result of GSEA indicated that the Notch signaling pathway was the only significant pathway enriched in the high CLK2 expression group (Figure 4(a), Supporting Information Table S1). We further explored the differential AS event prevalence between normal and tumor samples (Supporting Information Table S2) and the relationship between CLK2 expression and PSI value of differential splicing genes (Supporting Information Table S3). Interestingly, CTBP1, a key gene in the Notch pathway, was closely associated with the functions of CLK2. The results suggested that AP of CTBP1 was significantly increased in CRC tissue than normal tissue (Figure 4(b)), and the CLK2 expression was positively associated with AP event of CTBP1 (Figure 4(c), cor = 0.474, P = 1.697*e* − 33). In addition, we explored the expression level of CTBP1 and the relationship between CTBP1 and CLK2. And the results showed that CTBP1 was upregulated in CRC tissue than normal tissue and positively correlated with CLK2 in transcriptional level (Figures 4(d) and 4(e)). Taken together, it is indicated that CLK2 might involve in Notch signaling pathway by regulating the AS of CTBP1.

## 4. Discussion

At present, many CRC patients who have underwent surgical resection require a series of examinations including status of some specific genes and blood test, to predict the prognosis of patients and evaluate the recurrence risk and the sensitivity of antitumor drugs [4]. However, the 5-year overall survival of CRC is still unsatisfactory, especially in patients with advanced CRC. Therefore, it is essential to explore novel prognostic biomarkers and therapeutic targets for patients with CRC. In the present study, we found that the CLK2 expression was elevated in CRC tissue compared to normal tissue in transcription and protein level. The CRC patients with higher expression of CLK2 had a longer survival time and favorable prognosis. Univariate and multivariate Cox regression analyses further validated the CLK2 expression to be an essential risk factor for the prognosis of the patients with CRC. These results suggested that CLK2 was an oncogene and associated with poor prognosis. Consistent with these results, a previous study indicated that CLK2 functions as an oncogenic kinase, and high expression of CLK2 is correlated with poor survival in breast cancer [8]. Our study provides the first evidence that the expression of CLK2 may serve as a prognostic biomarker and therapeutic target for CRC patients.

Currently, the immune microenvironment was found strongly associated with therapeutic strategy of CRC [25]. We performed differential analysis between high and low CLK2 expression groups, and the DEGs were obtained for GO enrichment analysis. The results showed that DEGs were enriched in regulation of humoral immune response, regulation of immune effector process, and humoral immune response mediated by circulating immunoglobulin, which indicated that the CLK2 expression may be associated with immune regulation. We further evaluated the relationship between CLK2 expression and the immune landscape in CRC. The results suggested that the high expression of CLK2 significantly inhibited plasma cells and eosinophil infiltration. A previous study indicated that CD138+ plasma cell infiltration was correlated with better prognosis in colorectal cancer [26]. Another study suggested that eosinophil infiltration may activate the immune system of CRC patients because they are closely associated with some important prognostic factors, such as TNM stage and age [27]. These results suggest that the prognostic value of CLK2 may be associated with plasma cell and eosinophil infiltration. We further evaluated the difference of immune and stromal scores between high and low CLK2 expression group. However, immune and stromal scores did not show association with the CLK2 expression.

The molecular mechanisms that CLK2 involved in colorectal cancer have rarely been reported. In our study, we performed GSEA and found that only one signaling pathway—Notch signaling pathway—was enriched in the high CLK2 expression group. As we known, CLK2 gene encodes a type of protein kinase that phosphorylates serine/arginine-rich proteins (SR proteins), which plays essential roles in the AS of pre-mRNA in various cancers [28]. We therefore speculated that CLK2 may function as splicing factor to regulate the gene expression in Notch signaling pathway. AT event of Numb gene was correlated with the activation of Notch pathway in lung cancer [29, 30]. Aloe vera regulates Notch signaling pathway through AS in colorectal cancer cell [31]. These studies revealed that Notch signaling pathway was closely associated with AS in various cancer. Hence, we further explored the functions of CLK2 in regulating AS and found that the CLK2 expression was positively associated with AP event of CTBP1. CTBP1 is a key gene in the Notch signaling pathway and encodes a transcriptional corepressor protein that plays crucial roles in tumor development and progression [32–34]. Additionally, the functions of CTBP1 are regulated by AS in some ways. AS product of CTBP1 is correlated with cell proliferation and migration in melanoma, and full length of CTBP1 do not show difference [35]. AS of CTBP1 plays essential roles in chemoresistance in breast cancer [36]. Collectively, these results suggest that CLK2 may involve in Notch signaling pathway by regulating the AS of CTBP1.

Up to date, several CLK2 inhibitors have been investigated in experiment or clinical traits in various cancers [11, 14, 15, 37, 38]. For example, CLK2 inhibitor T-025 inhibits tumor cell proliferation via inducing exon skipping in MYC-driven cancers [11, 14]. However, several shortcomings limit their clinical application. First, specificity of the inhibitors is not very high. Second, the poor application exists in different cancers, which suggests that CLK2 plays different roles in different tumors. Therefore, it is essential to explore the potential molecular mechanisms of CLK2 in different cancers. Our study provides a novel research direction to explore the roles of CLK2 in CRC.

## 5. Conclusions

Our study demonstrates that the elevated expression of CLK2 significantly correlates with local invasion, distal metastasis, and prognosis of patients with colorectal cancer. The high expression of CLK2 significantly inhibits plasma cells and eosinophil infiltration. CLK2 may involve in Notch signaling pathway by regulation of CTBP1 AS. Our study suggests that CLK2 may be a potential prognostic biomarker and therapeutic target for colorectal cancer.

## Figures and Tables

**Table 1:** Association with CLK2 expression and clinicopathologic characteristics in TCGA and our validation cohort.

	Total	CLK2 expression		*Χ* ^2^	P
		Low expression	High expression		
TCGA cohort					
Age					
<65	217	119	98	3.423	0.064
≥65	317	148	169		
Gender					
Female	247	112	135	3.985	**0.046**
Male	287	132	155		
Radiation therapy					
No	493	245	248	0.238	0.626
Yes	41	22	19		
Chemotherapy					
No	322	164	158	0.282	0.596
Yes	212	103	109		
Local invasion					
T1	19	12	7	12.799	**0.005**
T2	94	60	34		
T3	365	174	191		
T4	56	21	35		
LN metastasis					
N0	303	159	144	5.766	0.056
N1	132	70	62		
N2	97	38	59		
Distant metastasis					
Yes	80	37	43	0.511	0.540
No	399	202	197		
TNM stage					
Stage I	94	34	60	8.795	**0.032**
Stage II	197	94	103		
Stage III	163	76	87		
Stage IV	80	43	37		
Validation cohort					
Age					
<65	15	10	5	0.321	0.571
≥65	35	20	15		
Gender					
Female	26	14	12	2.381	0.123
Male	24	11	13		
Tumor size					
<5 cm	26	15	11	1.282	0.258
≥5 cm	24	10	14		
Tumor grade					
G1 + G2	23	15	8	3.945	**0.047**
G3	27	10	17		
Local invasion					
T1 + T2 + T3	23	17	6	9.742	**0.002**
T4	27	8	19		
LN metastasis					
Yes	14	10	4	3.571	0.059
No	36	15	21		
TNM stage					
Stage I/II	14	10	4	3.571	0.059
Stage III	36	15	21		

Bold, P < 0.05, demonstrated by chi-square test. Abbreviations: LN: lymph node metastasis.

**Table 2:** Logistic regression of ARMCX1 expression and clinicopathologic parameters in TCGA and our validation cohort.

Clinical features		TN	OR	95% CI	P value
TCGA cohort					
Age	≥65 vs. <65	534	1.387	0.981-1.963	0.064
Gender	Female vs. male	534	0.707	0.502-0.994	0.046
Radiation therapy	Yes vs. no	534	0.853	0.447-1.616	0.626
Chemotherapy	Yes vs. no	534	1.098	0.777-1.555	0.596
Local invasion	T2 vs. T1	113	1.435	0.533-4.011	0.478
	T3 vs. T1	384	1.762	0.693-4.828	0.245
	T4 vs. T1	75	8.242	2.407-38.475	0.002
LN metastasis	Yes vs. no	532	0.965	0.640-1.453	0.865
Distant metastasis	Yes vs. no	479	1.204	0.744-1.954	0.450
TNM stage	Stage II vs. stage I	291	1.446	0.883-2.378	0.144
	Stage III vs. stage I	257	1.696	1.018-2.846	0.044
	Stage IV vs. stage I	174	1.592	0.875-2.915	0.129
Validation cohort					
Age	≥65 vs. <65	50	1.379	0.453-4.264	0.571
Gender	Female vs. male	50	0.375	0.098-1.286	0.129
Grade	G3 vs. G1 + G2	50	3.188	1.023-10.602	0.781
Tumor size	≥5 cm vs. <5 cm	50	1.909	0.626-6.023	0.050
Local invasion	T4 vs. T1 + T2 + T3	50	6.729	2.035-25.083	0.003
LN metastasis	Yes vs. no	50	3.500	0.971-14.781	0.066
TNM stage	Stage III vs. stage I/II	50	3.500	0.971-14.781	0.066

Abbreviations: OR: odds ratio; TN: total number; CI: confidence interval; LN: lymph node.

**Table 3:** Univariate and multivariate analysis of clinical features associated with the expression level of CLK2 in colorectal cancer.

Clinical features	Univariate analysis		Multivariate analysis		
	HR	P value	HR	95% CI	P value
Age (≥65 vs. <65)	2.09	0.002	2.70	1.68-4.35	<0.001
Gender (female vs. male)	1.12	0.597	—	—	—
TNM stage (stages III and IV vs. stage III and IV)	3.76	<0.001	1.33	1.13-12.59	0.030
Local invasion (T4 vs. T1-T3)	3.88	<0.001	2.30	1.32-4.02	0.003
Lymph metastasis (yes vs. no)	3.23	<0.001	0.75	0.26-2.19	0.598
Distant metastasis (yes vs. no)	4.35	<0.001	2.30	1.34-3.85	0.002
CLK2 expression (high vs. low)	2.01	0.002	1.71	1.09-2.69	0.019

Note: HR: hazard ratio.

## Data Availability

All analyzed data are included in this published article and its supplementary information file. The original data are available upon reasonable request to the corresponding author.

## Abbreviations

CRC: Colorectal cancer
TCGA: The Cancer Genome Atlas
GO: Gene Oncology
GSEA: Gene set enrichment analysis
AS: Alternative splicing
COAD: Colon adenocarcinoma
READ: Rectum adenocarcinoma
PSI: Percent-Spliced-In
DEGs: Differentially expressed genes
AA: Alternate acceptor site
AD: Alternate donor site
AP: Alternate promoter
AT: Alternate terminator
ES: Exon skip
ME: Mutually exclusive exons
RI: Retained intron.

## Supplementary Materials

Supplementary 1 Table S1: the top 5 signaling pathways enriched in the high and low CLK2 expression group.

Supplementary 2 Table S2: the differential analysis of AS event prevalence between normal and tumor samples.

Supplementary 3 Table S3: the relationship between CLK2 expression and PSI value of differential splicing genes.
