# Supplementary material for: CLK2 Expression Is Associated with the Progression of Colorectal Cancer and Is a Prognostic Biomarker
Source: Biomed Res Int. 2022 Jul 7;2022:7250127. doi: 10.1155/2022/7250127 (PMC9289758; doi:10.1155/2022/7250127)
Supplement: Supplementary 1 — Table S1: the top 5 signaling pathways enriched in the high and low CLK2 expression group. [file 7250127.f1.pdf]

Supporting Information Table S1 The top 5 signaling pathways enriched in high and low CLK2 expression group

Signaling pathway	NES	NOM p_value	FDR q_value
KEGG_NOTCH_SIGNALING_PATHWAY	2.09	0.000	0.022
KEGG_PHOSPHATIDYLINOSITOL_SIGNALING_SYSTEM	1.95	0.002	0.079
KEGG_INOSITOL_PHOSPHATE_METABOLISM	1.95	0.002	0.056
KEGG_GNRH_SIGNALING_PATHWAY	1.86	0.000	0.115
KEGG_GLYCEROPHOSPHOLIPID_METABOLISM	1.82	0.000	0.125
KEGG_OXIDATIVE_PHOSPHORYLATION	-1.63	0.070	1.000
KEGG_PARKINSONS_DISEASE	-1.49	0.139	1.000
KEGG_PENTOSE_AND_GLUCURONATE_INTERCONVERSIONS	-1.34	0.147	1.000
KEGG_PRIMARY_BILE_ACID_BIOSYNTHESIS	-1.29	0.138	1.000
KEGG_PEROXISOME	-1.24	0.195	1.000

Note: NOM p_value <0.05 and FDR q_value <0.05 were considered as significantly enriched. NES, normalized enrichment score; NOM, nominal; FDR, false discovery
