# Supplementary material for: CLK2 Expression Is Associated with the Progression of Colorectal Cancer and Is a Prognostic Biomarker
Source: Biomed Res Int. 2022 Jul 7;2022:7250127. doi: 10.1155/2022/7250127 (PMC9289758; doi:10.1155/2022/7250127)
Supplement: Supplementary 2 — Table S2: the differential analysis of AS event prevalence between normal and tumor samples. [file 7250127.f2.pdf]

Supporting Information Table S2 The differential analysis of AS ev

AS event	normalMean	TumorMean	logFC
ABCC3_RI	0.150049095	0.36118366	1.267298005
ABCC3_RI	0.212256259	0.431761643	1.02442799
ABCC3_AA	0.047651769	0.108545369	1.187696489
ABCD1_AT	0.052199088	0.124097286	1.249375049
ABCD4_AA	0.035765307	0.11015116	1.622851937
ABCD4_AD	0.031655058	0.081929343	1.371944215
ABCE1_ES	0.383403571	0.184965605	-1.051606754
ABHD3_AD	0.049441183	0.143985752	1.542140888
ABI1_ES	0.059354092	0.132816194	1.162011666
ABLIM2_AT	0.137921205	0.383058408	1.473720109
ABTB1_RI	0.023885006	0.058669499	1.296505419
ACAA1_RI	0.089896487	0.227860711	1.341815539
ACADVL_RI	0.047309664	0.118714866	1.327293775
ACAN_AT	0.523277769	0.075107754	-2.800543212
ACOT7_AP	0.173920583	0.415424362	1.256157142
ACOT8_ES	0.022958806	0.061642672	1.424881783
ACP1_RI	0.058334124	0.126201281	1.11331458
ACP5_AP	0.2360567	0.110584094	-1.093989541
ACSL5_AP	0.307332696	0.116794705	-1.395826385
ACSS2_AP	0.561537746	0.224874779	-1.320261141
ACTA2_AP	0.022958454	0.056709824	1.30457319
ACTR6_AD	0.107955629	0.051495183	-1.067929066
ACTR8_AP	0.028031253	0.057307595	1.031690134
ADAM15_AD	0.041711932	0.160079993	1.940260986
ADAMTS12_AT	0.269840038	0.044928199	-2.586411304
ADAMTS2_AT	0.397728911	0.135437648	-1.554156611
ADAMTSL1_AT	0.020278181	0.054596581	1.428882399
ADAP1_AP	0.14058422	0.060373431	-1.219448958
ADAP1_AP	0.035591808	0.106555194	1.581983773
ADCK5_RI	0.050517563	0.161736578	1.678789045
ADCK5_ES	0.044799579	0.132170333	1.560841309
ADCY7_AT	0.049679631	0.111173337	1.162084447
ADM_RI	0.031806575	0.066330923	1.06035656
AFMID_ES	0.224715032	0.112271573	-1.001103946
AFMID_ES	0.401380919	0.189881175	-1.079875148
AHCYL2_AP	0.013535704	0.1827665	3.755159864
AHNAK_AT	0.060735454	0.124615793	1.036876079
AKAP17A_ES	0.171977818	0.081572721	-1.076063822
AKR1A1_AT	0.021907699	0.062763006	1.518476496
ALDH18A1_AD	0.205086856	0.437905148	1.094383375
ALDH3A2_ES	0.030013009	0.060710939	1.016368519
ALS2CL_AA	0.060204574	0.243292602	2.014747454
ALS2CL_RI	0.125787876	0.272763439	1.116657412
ANAPC13_AD	0.028924725	0.058034711	1.004612821
ANAPC15_AP	0.0277189	0.061926964	1.159697726
ANGPT2_AT	0.1508219	0.072189248	-1.062990055
ANO7_AT	0.083689161	0.265423241	1.665182005
ANTXR1_AT	0.067996465	0.146938661	1.111682376
ANXA11_ES	0.093153727	0.214449245	1.202950839
AP1G2_AD	0.051531043	0.116786525	1.180360136

AP1S2_AT	0.224473437	0.506531558	1.174107421
APOC2_RI	0.581390624	0.236673823	-1.296607661
APTX_AP	0.024545962	0.053951073	1.136165963
AQP1_AP	0.048666947	0.219050047	2.170246335
ARAP1_AP	0.016076997	0.070614457	2.134965645
ARAP1_AP	0.089600053	0.196771605	1.134950548
ARHGAP27_AT	0.018774364	0.05792016	1.625301581
ARHGEF16_AP	0.049794392	0.101865458	1.03260975
ARL4A_ES	0.14458979	0.311801925	1.108664151
ARL6IP4_RI	0.020050594	0.063415085	1.661181113
ARMCX5_AD	0.078344978	0.17096105	1.125754976
ARPC1B_ES	0.128126268	0.062461496	-1.036527253
ARSE_AP	0.038127005	0.097385507	1.352893864
ATP6V0D1_AP	0.156482678	0.047841848	-1.709657935
AUP1_AA	0.036362485	0.072959652	1.004648058
AXL_AP	0.029094407	0.061023186	1.068615683
AZI2_AT	0.428279993	0.207807103	-1.043309317
B3GAT3_ES	0.018117621	0.052727697	1.541167437
B3GNT5_AP	0.025695802	0.05884164	1.195304784
B9D1_AP	0.101602189	0.049735533	-1.03058266
BBS1_RI	0.03960034	0.114503926	1.531812342
BBS1_ES	0.040204561	0.128239743	1.673412347
BCAM_AT	0.113377142	0.313290986	1.466373457
BCAR3_AP	0.019999582	0.070548569	1.81864695
BCAR3_AP	0.026428483	0.128456693	2.281116541
BCAT2_ES	0.041892798	0.096700312	1.2068183
BCL2L11_AP	0.019514723	0.065984635	1.75756717
BCS1L_AD	0.152678115	0.367602607	1.267653714
BCS1L_AD	0.043818494	0.149443552	1.76998885
BLCAP_AP	0.018105252	0.05401367	1.576916355
BLOC1S1_AP	0.021777275	0.067971161	1.642099338
BMP1_ES	0.33677073	0.148026433	-1.185911939
BNIP2_AP	0.024619496	0.055833825	1.181338145
BRD9_AP	0.02655904	0.087599674	1.721722492
BRD9_ES	0.026710793	0.054951895	1.040746403
BTNL9_AT	0.042493162	0.098332026	1.210430658
C10orf118_AT	0.025922802	0.063334842	1.28877775
C11orf54_ES	0.04210856	0.088772477	1.075998917
C16orf58_RI	0.07886809	0.167131126	1.083466821
C17orf62_AP	0.038464388	0.090444513	1.233509616
C1orf106_AP	0.017416279	0.054443169	1.644314648
C1orf131_RI	0.039585144	0.08669206	1.130940771
C1orf198_AP	0.022188591	0.054209003	1.288714389
C1orf54_AP	0.257112933	0.121482646	-1.081651944
C1RL_AT	0.036418286	0.101203847	1.474529182
C1S_AP	0.007577411	0.063248922	3.061263959
C20orf96_AP	0.030788856	0.061830811	1.005917663
C2CD5_ES	0.121229645	0.055513919	-1.126821094
C2orf54_AP	0.332956878	0.032986564	-3.335384946
C4orf19_AT	0.040193726	0.081071813	1.012230086
C5orf30_AP	0.046482516	0.125695396	1.435171725
C5orf30_AP	0.049987655	0.120354521	1.267646595
CAB39_AP	0.040470963	0.083666358	1.04776045

CABIN1_AP	0.247928204	0.111469288	-1.153276122
CALD1_ES	0.800814046	0.249778387	-1.680818632
CALD1_ES	0.133520765	0.058501567	-1.190516947
CAMKK2_ES	0.156890312	0.058122343	-1.432591505
CAPN12_AT	0.417468816	0.194521788	-1.101736675
CAPN13_AT	0.019572064	0.115873173	2.565678776
CAPN2_AP	0.026376063	0.053460108	1.0192335
CARKD_RI	0.095175551	0.211260681	1.150361353
CAST_ES	0.148223575	0.067583185	-1.133038675
CBFA2T2_AP	0.117434759	0.247655358	1.076474343
CBR4_ES	0.033715135	0.093365503	1.46949324
CCAR2_AP	0.04277364	0.134871014	1.656786431
CCDC107_RI	0.075231455	0.162863491	1.114255336
CCDC64B_AP	0.263649644	0.540286043	1.035101362
CCDC7_AT	0.034675612	0.073302242	1.079935969
CCL14_AP	0.509355037	0.221194774	-1.203354313
CCL24_AP	0.522371832	0.109566713	-2.253267536
CCL28_AP	0.018358555	0.131710469	2.842845607
CCNDBP1_AA	0.023128164	0.056711604	1.293993252
CCS_AA	0.019509137	0.061633503	1.659564785
CCSER2_ES	0.359489103	0.122929693	-1.548114611
CD276_AP	0.024558638	0.058140153	1.243304339
CD44_ES	0.016107363	0.108176261	2.747591738
CD44_ES	0.025212659	0.255356393	3.340291998
CD44_ES	0.01223551	0.089557731	2.871743744
CD44_ES	0.020616115	0.21690526	3.39522066
CD44_ES	0.147448874	0.528977202	1.842990744
CD44_ES	0.11506954	0.296038331	1.363278004
CD44_ES	0.349992506	0.77175611	1.140820968
CD55_AT	0.279547539	0.10855665	-1.364645542
CDIP1_AP	0.054279358	0.242585764	2.160019333
CDIP1_AP	0.131639684	0.063768637	-1.045675513
CDK16_AP	0.031530534	0.064641776	1.035717254
CDK18_RI	0.085341633	0.204000998	1.257254582
CDK5RAP3_RI	0.115265147	0.272057461	1.238955043
CDKN2A_AP	0.131880405	0.390165487	1.564855943
CENPM_AP	0.042968019	0.116741894	1.441987209
CERS5_ES	0.030299167	0.068170787	1.169875526
CES2_AD	0.01255284	0.084887696	2.757541696
CES3_AP	0.065720011	0.213069948	1.696922497
CGREF1_AP	0.161087253	0.577232605	1.841310454
CGREF1_AP	0.833217834	0.381233545	-1.128018651
CHD6_AT	0.025509642	0.052742152	1.047913792
CHMP2A_AP	0.065086049	0.131379939	1.013324761
CKMT1A_AA	0.025331849	0.0635718	1.327434554
CLASP2_AP	0.236898081	0.046636133	-2.344746446
CLDN11_AT	0.534082788	0.264198589	-1.015440629
CLDN7_AP	0.495849401	0.240098006	-1.046278588
CLEC16A_AT	0.036287084	0.079664108	1.134473757
CLEC5A_AT	0.041473786	0.104404167	1.331907623
CLIP4_AT	0.064241466	0.134036576	1.061050009
CLK3_AP	0.247947259	0.101591492	-1.287253692
CLN3_AP	0.028203273	0.123588282	2.13160744

COASY_AA	0.083111373	0.182007347	1.130878874
COL12A1_ES	0.464711073	0.990616025	1.091991938
COL18A1_AP	0.118563124	0.45448174	1.93856696
COL6A3_ES	0.146963581	0.616909838	2.069600962
COL6A3_ES	0.056677791	0.15297161	1.432408477
COMT_AA	0.023565574	0.057876309	1.296292105
COQ10A_AP	0.429519553	0.160416684	-1.420899615
COQ4_AT	0.098891828	0.203062558	1.038001047
CORIN_AT	0.11310578	0.319210927	1.49683738
CPED1_AT	0.052060836	0.134505951	1.369399624
CPSF3L_AA	0.024775553	0.070238799	1.503350936
CRAT_AT	0.030436815	0.07044482	1.210676235
CRAT_ES	0.214873607	0.095993381	-1.162481451
CRB3_AP	0.635198539	0.248864615	-1.351846478
CRB3_AP	0.358460413	0.743747271	1.052998669
CREB3L4_RI	0.061065651	0.125916867	1.044038528
CRTC1_ES	0.17601595	0.082787883	-1.088214642
CRYAB_AP	0.185301463	0.077836785	-1.251350247
CSTF3_RI	0.093419103	0.203756623	1.125057457
CTNNBIP1_AP	0.007322071	0.091389091	3.641698274
CTTN_ES	0.032274213	0.103921028	1.687033803
CUX1_AP	0.023840536	0.06081741	1.351067721
CXCL12_AT	0.153865667	0.468554209	1.606544622
CXCL12_AT	0.034780775	0.122338591	1.814517572
CYB561A3_RI	0.025501476	0.071967068	1.496756139
CYB561A3_RI	0.024770211	0.079277409	1.678303684
CYGB_AP	0.020903974	0.0672171	1.685051064
CYP3A5_AA	0.023246823	0.089069137	1.93789208
CYP3A5_ES	0.038845954	0.089242539	1.199967233
CYP4F12_RI	0.169940883	0.374910233	1.141512238
CYP4F12_RI	0.036557832	0.093633545	1.356844949
DAPK2_ES	0.079828675	0.222922912	1.481565943
DBN1_AP	0.019160413	0.054340295	1.503893694
DBN1_ES	0.20313682	0.042318532	-2.263090287
DBNDD1_AP	0.109160139	0.431413622	1.9826256
DBNDD2_AP	0.07264375	0.171047033	1.235482496
DCST1_AT	0.042850097	0.093211713	1.121212784
DCTD_ES	0.044412504	0.089597053	1.01248536
DCTPP1_AP	0.193580275	0.060586841	-1.675855577
DCUN1D4_AD	0.079559854	0.187830798	1.239321094
DDB1_AP	0.022127751	0.061784527	1.481388758
DDRGK1_AT	0.023929231	0.059304472	1.309366865
DECR2_ES	0.107041683	0.226173188	1.079255211
DENND2A_AT	0.095299203	0.225726045	1.244036835
DENND5B_AT	0.037292375	0.104518925	1.48681162
DGAT1_RI	0.02229823	0.079399037	1.832192348
DGKZ_AP	0.162974305	0.077839954	-1.066061767
DHDDS_AA	0.022852165	0.052963746	1.212674309
DHRS1_RI	0.113032584	0.286484372	1.341717725
DHRSX_AP	0.203796052	0.072289071	-1.495276652
DIXDC1_AT	0.03958697	0.142422769	1.847082251
DMKN_AP	0.434196787	0.092004336	-2.238575298
DMPK_AP	0.137083687	0.307110088	1.163698999

DNAH2_AT	0.281332265	0.665244759	1.241610222
DNAH2_AT	0.719178974	0.305502498	-1.235166668
DNAJA4_AP	0.035214304	0.098708392	1.487011159
DNAJA4_AP	0.021421714	0.062059048	1.534567654
DNAJC2_AT	0.173012799	0.077094672	-1.166175706
DNMT3A_AT	0.194679943	0.059792465	-1.703068676
DOK1_AP	0.04268013	0.176968456	2.051855773
DPP8_AP	0.020283664	0.061807079	1.607453795
DPYSL2_AP	0.046506112	0.159971499	1.78232264
DTNA_AT	0.083103033	0.226938963	1.449331288
DTNA_AT	0.027926287	0.068067246	1.285336987
DUSP10_AP	0.042159507	0.105261915	1.320053629
DUSP15_AT	0.088393201	0.323870401	1.873409319
DYNC1I2_ES	0.440681171	0.201632433	-1.128007538
DYX1C1_AT	0.1807156	0.080084079	-1.174133693
DYX1C1_AT	0.072845859	0.206708915	1.504681736
E2F5_AP	0.364655512	0.060025966	-2.602875582
ECE1_AP	0.048513357	0.098477098	1.021406233
ECHDC2_ES	0.100364839	0.201154037	1.00304675
ECHDC2_ES	0.040704428	0.101306175	1.315464454
ECHDC2_ES	0.154563484	0.338961378	1.132921382
EEF1A1_ES	0.117541608	0.332884671	1.501850898
EEF1A1_RI	0.106674899	0.292921845	1.457295042
EEF1D_AP	0.033016613	0.104243908	1.658699037
EEF1D_ES	0.222122998	0.444547435	1.000978594
EGFL7_AP	0.093198018	0.20890561	1.164480063
EGLN3_AT	0.027132413	0.094745291	1.80403687
ELF3_RI	0.023854652	0.075709354	1.666200913
ELK4_AT	0.07901095	0.163016136	1.044890259
EP400NL_AT	0.036485418	0.309185537	3.08308096
EP400NL_AT	0.016258029	0.086005318	2.403273517
EPB41L1_AP	0.016329061	0.065558227	2.005335036
EPB41L2_ES	0.481213175	0.159945316	-1.589097401
EPS8_AP	0.297612137	0.135605054	-1.134022409
EPS8L1_ES	0.070863044	0.145898613	1.041860824
EPS8L2_RI	0.029102516	0.079103255	1.442593203
ERBB3_AP	0.014756726	0.056146016	1.927810997
ERN2_RI	0.244701781	0.508807946	1.056096601
ETS1_AP	0.018908953	0.079737653	2.076191625
EXOC7_ES	0.089595228	0.214600342	1.260158581
FAM102A_AP	0.025209103	0.0526492	1.062466793
FAM134B_AP	0.097203975	0.255668801	1.395188896
FAM213A_AP	0.016701847	0.055570155	1.734302577
FAM3D_AP	0.014646283	0.127269446	3.11927963
FAM60A_AP	0.477387443	0.145099322	-1.718119843
FAM69B_AP	0.365897469	0.175037143	-1.063778336
FAM72A_AP	0.240716764	0.098789809	-1.284902486
FAM72A_AT	0.216336895	0.080540929	-1.425485713
FAM73B_AA	0.047149468	0.118017007	1.323681398
FANCD2_AT	0.130740696	0.05333982	-1.293423419
FARS2_AP	0.054752591	0.120510453	1.138159153
FASTK_RI	0.046875369	0.100997668	1.107420012
FASTK_RI	0.085005413	0.187394832	1.140454542

FASTK_RI	0.058247245	0.15080289	1.372402356
FASTK_RI	0.039048538	0.115480674	1.564310984
FBXO3_AP	0.024860605	0.055115162	1.148587834
FDPS_ES	0.038224554	0.077435794	1.018500911
FDPS_ES	0.028269306	0.073031292	1.369278268
FGFR1_AP	0.156459072	0.067364998	-1.215714223
FKBP10_AD	0.017977445	0.071557714	1.992919281
FLCN_AT	0.067979287	0.161838049	1.251383709
FLNA_ES	0.225991516	0.574419671	1.345836538
FLNB_ES	0.432645856	0.213209856	-1.020912456
FMO5_AT	0.020224696	0.064527503	1.673796209
FOXRED1_AD	0.036765129	0.085677668	1.220581168
FUK_AT	0.026898221	0.05646954	1.069962129
FXYD3_RI	0.082657012	0.199037178	1.267828822
FXYD3_RI	0.049506593	0.110991042	1.164750677
FYN_AP	0.395632955	0.165604995	-1.256416412
GABRE_AT	0.143774368	0.067131178	-1.098751633
GATA6_AP	0.055621936	0.114650159	1.043512496
GCDH_AD	0.016280354	0.056069316	1.784079416
GFER_AP	0.416620514	0.195055933	-1.094845993
GGA3_RI	0.120635085	0.247982931	1.039591262
GGT1_AP	0.034445578	0.095360243	1.469069132
GGT1_AP	0.067953382	0.148366531	1.126548409
GGT1_AA	0.042217015	0.100351327	1.249163196
GHDC_AA	0.069104814	0.14451322	1.064343355
GINS4_AT	0.278142353	0.127137682	-1.129431749
GJB1_AP	0.017833787	0.064704831	1.859260316
GK_ES	0.144093732	0.435033831	1.594120013
GLS_AP	0.028569836	0.080510402	1.49468243
GLUL_AD	0.126904704	0.295019419	1.217064368
GPS1_RI	0.18657853	0.392229275	1.071914236
GPS1_AD	0.017708991	0.06048343	1.772057951
GPS2_RI	0.091786188	0.250160645	1.446505872
GRB2_AP	0.175138625	0.087217897	-1.005801176
GRB7_AP	0.10533166	0.281108834	1.416189656
GREB1_AT	0.035064451	0.093624254	1.416873186
GREB1_AT	0.367622454	0.182147255	-1.013119631
GRK6_AT	0.019127391	0.062604155	1.710618291
GRSF1_AD	0.12770699	0.061098344	-1.063632307
GSDMB_AP	0.064718017	0.211202054	1.70638456
GSKIP_AD	0.054578116	0.110275883	1.014722809
GTF2IRD1_AP	0.397598476	0.172532711	-1.204442311
HDAC10_RI	0.071894708	0.146452712	1.026477424
HDAC4_AT	0.030238233	0.100662727	1.735083864
HELZ2_AP	0.018275675	0.083857736	2.198019161
HEMK1_RI	0.024335202	0.055628055	1.192767937
HES6_RI	0.085489591	0.17249992	1.012775011
HEXB_AP	0.063931973	0.131089695	1.03594477
HIST1H2BD_AT	0.012554006	0.067732441	2.431699152
HIST1H2BN_AT	0.042479921	0.108291711	1.350069832
HM13_ES	0.480711446	0.225148044	-1.094297208
HMG20B_AA	0.026666274	0.054513839	1.031606256
HMGA1_AP	0.036975723	0.075732031	1.03432529

HNF4A_AP	0.490496905	0.101918421	-2.266829204
HNMT_AT	0.098065611	0.212380659	1.114833176
HNRNPA1_ES	0.121179845	0.254244054	1.069064275
HNRNPA1_RI	0.062517213	0.129339119	1.048833328
HOOK2_ES	0.038082437	0.078669108	1.046671403
HP1BP3_AT	0.029639831	0.071186777	1.26407204
HTRA3_AT	0.140642434	0.068073527	-1.046866184
IAH1_AP	0.030354036	0.074259237	1.29068213
ICAM3_RI	0.179219038	0.471843773	1.396585367
IDS_AP	0.075478531	0.234898229	1.637897578
IFNLR1_AT	0.042987451	0.093435975	1.120062569
IL11RA_AP	0.667782404	0.328351346	-1.024137709
IL11RA_AP	0.202508888	0.550447041	1.442618541
IL1RAP_AT	0.122545206	0.050291128	-1.284938224
IL1RL1_AT	0.322900188	0.146916751	-1.136089382
IL4R_ES	0.054518089	0.128632521	1.238448537
IMMP2L_AP	0.067859466	0.202588515	1.577930396
INADL_AT	0.053571056	0.130768239	1.287486531
INPPL1_AP	0.054332173	0.12635448	1.217598162
INTS3_RI	0.043031971	0.103095544	1.260501159
IP6K2_ES	0.118608386	0.053530004	-1.147786344
IPO11_AT	0.13751944	0.06323992	-1.120728132
IPO13_AP	0.04420887	0.099646153	1.172478256
ISCU_ES	0.048963698	0.106802532	1.125161412
ISLR_AP	0.140986189	0.637778	2.177500489
ISLR_AP	0.857471371	0.360772036	-1.249000987
ITGB3BP_ES	0.300018687	0.145620887	-1.042835063
JTB_RI	0.029115464	0.070258474	1.270886649
KALRN_AP	0.170045983	0.461792225	1.441318956
KCND1_AP	0.012047016	0.055656601	2.207876966
KCTD10_AP	0.10410845	0.216012984	1.053030856
KIF16B_AT	0.32393552	0.707029111	1.126062948
KIF16B_AT	0.67585998	0.292257306	-1.209485301
KIF4A_AT	0.26241194	0.078979057	-1.732291319
KIF9_ES	0.037399565	0.075030668	1.004458893
KLF3_AT	0.017417069	0.102636361	2.558968146
KLHDC2_RI	0.063666833	0.131773489	1.049446239
KLHL12_AP	0.026514676	0.059606578	1.168680433
KLK10_AP	0.184018407	0.537070618	1.545261711
LAMA3_AP	0.028079596	0.072957908	1.377542155
LARP6_AT	0.226351195	0.507024993	1.163493943
LAS1L_ES	0.210864848	0.057878315	-1.865223792
LBX2_AP	0.017190435	0.098346391	2.516266075
LEF1_AP	0.222890345	0.079035741	-1.495757008
LGALS3BP_ES	0.085662304	0.195139291	1.18777191
LGALS4_ES	0.031545064	0.240402966	2.929968537
LGALS4_ES	0.089216346	0.219456224	1.298553213
LIMA1_AP	0.048401801	0.122947948	1.344915006
LIMK2_AP	0.053006231	0.166699448	1.653015472
LIMS1_AP	0.145369866	0.067157342	-1.114111198
LINC00908_AT	0.247183175	0.099524942	-1.312450513
LMO7_AA	0.041871143	0.126334851	1.593224482
LMO7_ES	0.018581424	0.144842054	2.9625476

LPCAT3_RI	0.044162304	0.110947885	1.32899482
LPCAT3_RI	0.063333758	0.153302789	1.275337363
LRCH3_AT	0.026442051	0.056628714	1.098699666
LRP11_AP	0.261434428	0.119040443	-1.134997343
LRRC32_AP	0.113809457	0.247760378	1.122325044
LRRC36_AT	0.426106411	0.209662968	-1.023141692
LRRFIP1_ES	0.235510613	0.058955447	-1.998095059
LTBP3_AP	0.111567053	0.246541479	1.143919342
LTBR_AP	0.192441279	0.05930118	-1.698285584
LTBR_ES	0.023825279	0.051097153	1.100749832
LYNX1_AT	0.234232112	0.116791763	-1.004000342
LYRM1_AP	0.069164267	0.164217951	1.247513054
LYRM1_ES	0.080661506	0.161500644	1.001587677
MAF1_AP	0.034620934	0.069249055	1.000149739
MAN2C1_AA	0.114653836	0.23153978	1.013975453
MAP2K5_AP	0.204485256	0.048372221	-2.079746132
MAPKAP1_AT	0.062916003	0.129082945	1.036799468
MAPRE2_AP	0.064975896	0.132110328	1.023766737
MARK3_ES	0.24705561	0.09413788	-1.391988554
MAST1_AT	0.018554641	0.107648275	2.536473207
MATR3_AP	0.03967138	0.117503358	1.566531511
MBOAT7_AT	0.023097802	0.051558379	1.158451354
MCF2L_AP	0.038129117	0.152278684	1.997748978
MCF2L_AP	0.262421085	0.112630327	-1.220288304
MCF2L_AP	0.0434143	0.08841309	1.026089656
MCMDC2_AT	0.293467999	0.031603838	-3.215031536
MED11_AA	0.085257055	0.205986647	1.272659681
MEIS3_AA	0.024405743	0.054901282	1.16961915
MELK_ES	0.530918719	0.201466492	-1.397951102
MESDC2_AT	0.073167826	0.160448481	1.132828831
METRNL_AP	0.023754016	0.0769448	1.6956524
MFSD10_AP	0.048953754	0.100752601	1.041325677
MGAT4B_AP	0.051883765	0.171523996	1.72505535
MID1_AP	0.020358483	0.064366255	1.660674457
MKL1_AP	0.1407987	0.064941252	-1.116426903
MLLT3_AP	0.530551493	0.181850899	-1.544736725
MLPH_ES	0.074880574	0.151344021	1.015168277
MLTK_AT	0.185022432	0.467748166	1.3380318
MLXIP_AP	0.02580801	0.085143894	1.722084201
MMP23B_RI	0.096784566	0.26937811	1.476783708
MOGAT3_RI	0.071497827	0.190533635	1.414074406
MON2_AP	0.030346237	0.075227688	1.309746109
MORF4L2_AP	0.022367505	0.056079682	1.326073862
MPI_AT	0.037521489	0.081930661	1.126686389
MROH1_RI	0.029360063	0.070200923	1.25763496
MRPL21_RI	0.016130439	0.054122425	1.746440796
MRPL52_RI	0.075303941	0.164229141	1.124912864
MRRF_AP	0.055242036	0.112953599	1.031891858
MSL3_AP	0.010512231	0.055083105	2.389540963
MYB_ES	0.136450904	0.064674828	-1.07710573
MYH11_ES	0.687293708	0.281523726	-1.28767024
MYH14_AP	0.006079528	0.059875017	3.299922838
MYO10_AP	0.061655316	0.146150165	1.245154257

NADK_AP	0.024161675	0.061606123	1.350353249
NADSYN1_ES	0.070000575	0.141588396	1.016264358
NAPRT1_RI	0.099020168	0.210725554	1.089570972
NARFL_AP	0.046000618	0.094838333	1.043817055
NCAM1_AT	0.386258105	0.128218447	-1.590961366
NCAPH2_AT	0.037450959	0.077667496	1.052308299
NDRG2_AP	0.036307128	0.092011368	1.34155933
NDRG2_AD	0.085239225	0.209198189	1.295280975
NDRG2_RI	0.027528953	0.075724881	1.459817657
NDUFA3_AT	0.115999275	0.240249798	1.050419425
NDUFS2_RI	0.017238725	0.063365783	1.878050904
NDUFS7_RI	0.025010254	0.053087521	1.085853046
NECAB3_RI	0.110646478	0.24718831	1.159652994
NEDD4L_AP	0.162971444	0.327321705	1.00609008
NEDD4L_AP	0.371384792	0.086441522	-2.103118359
NEDD9_ES	0.022663722	0.072816276	1.683876173
NEK3_AP	0.052667335	0.118666658	1.171934266
NET1_AP	0.014165693	0.067695341	2.256655368
NFASC_AT	0.245923275	0.531862258	1.11284438
NFIA_AP	0.002775797	0.067317097	4.599998937
NFYA_ES	0.219745367	0.102193334	-1.104531663
NGDN_RI	0.022659633	0.059561268	1.394249967
NHEJ1_ES	0.244608532	0.071776185	-1.768897572
NMT2_RI	0.027987254	0.060772188	1.118641312
NPIPB3_AP	0.101589571	0.21029665	1.049673564
NPLOC4_AP	0.017914542	0.09491306	2.405475456
NPRL2_RI	0.036660919	0.073512745	1.003751424
NR1H3_ES	0.037862817	0.08229032	1.119940985
NR1H3_RI	0.089188011	0.22185219	1.314677106
NR2F2_AP	0.167738778	0.061549389	-1.446399806
NRBP2_RI	0.145918089	0.327766211	1.16750839
NRG4_AT	0.18288154	0.045305585	-2.013148656
NSFL1C_ES	0.115607398	0.047075291	-1.296191818
NT5C3A_AP	0.498874857	0.177750912	-1.488820995
NT5C3A_ES	0.278924639	0.099826354	-1.482382741
NT5C3A_ES	0.114273466	0.050280942	-1.184406875
NT5C3A_ES	0.133301022	0.051022402	-1.385485115
NTMT1_AP	0.168513214	0.574216157	1.768732196
NTPCR_AT	0.070980575	0.160161848	1.174034372
OBSCN_AT	0.25670325	0.597461002	1.218742983
OCEL1_AP	0.061453112	0.163989766	1.416047805
OCIAD1_AD	0.052423984	0.105622285	1.010615345
ODF2_AP	0.02286399	0.104796079	2.196435618
OGFR_AP	0.041836014	0.083945815	1.004713024
OGFR_RI	0.027057232	0.069730933	1.365784543
OS9_ES	0.239995482	0.486106523	1.018265248
OSBPL1A_AP	0.345867467	0.733936374	1.085435683
OSBPL1A_AP	0.653734645	0.249116925	-1.391882115
OSBPL1A_AT	0.015684928	0.079146795	2.33515199
OSBPL5_AP	0.041476629	0.110543067	1.414237979
OSER1_AP	0.025701253	0.053600668	1.06041228
OSGEP_AD	0.040790194	0.113096309	1.471257583
P4HA3_AT	0.419332137	0.203815864	-1.040827051

PACS1_AP	0.040655615	0.082907585	1.028049452
PANK1_AP	0.266882078	0.126356818	-1.078698912
PAPSS2_ES	0.624623548	0.168608971	-1.889305658
PARD3B_AT	0.022410481	0.059556437	1.410083855
PCK2_AD	0.028546098	0.060459278	1.082670224
PCLO_AT	0.113050284	0.236482209	1.064767033
PCSK5_AT	0.022899959	0.131378917	2.520316828
PCSK5_AT	0.734731679	0.287146979	-1.355428096
PCSK5_AT	0.240253752	0.578046336	1.26662618
PCSK7_RI	0.035921013	0.091836351	1.354237295
PCYT1A_AT	0.203295301	0.049300871	-2.043891838
PCYT2_AA	0.043280181	0.097941548	1.178214456
PDDC1_AA	0.026442896	0.072395567	1.453021146
PDE4D_AP	0.476928966	0.201872084	-1.240332987
PELP1_ES	0.02755712	0.058178776	1.078067799
PEX26_AT	0.094390103	0.217863921	1.206719789
PGAP2_ES	0.032001937	0.090065502	1.492815367
PHLPP2_AT	0.112799292	0.282360379	1.323779656
PHYKPL_ES	0.0950736	0.192477722	1.017574785
PI4K2A_AP	0.041476352	0.168260377	2.020334555
PIGQ_AP	0.123139574	0.059936246	-1.03879386
PIK3R2_AT	0.031396795	0.070437367	1.165723688
PKN1_AP	0.324965691	0.15982777	-1.023769315
PLCD1_AP	0.47075305	0.163944003	-1.521767311
PLEC_AP	0.044601335	0.129602568	1.538935521
PLEKHA1_ES	0.203737061	0.077033954	-1.403142056
PLEKHJ1_RI	0.045871515	0.113485921	1.306842857
PLS1_AP	0.337391052	0.040066546	-3.07395168
PLSCR1_ES	0.3775572	0.180505229	-1.064654596
PLXNC1_AT	0.028927106	0.090319955	1.642622764
PMPCB_AT	0.097564536	0.048752726	-1.000873952
PODNL1_AP	0.100971849	0.321220505	1.669610858
POLR3H_ES	0.222207777	0.088452814	-1.328929366
POMGNT1_AP	0.251022904	0.098915639	-1.343548468
PPFIA1_AT	0.020159572	0.057322142	1.507627507
PPFIBP2_AP	0.052115932	0.207725133	1.9948794
PPFIBP2_AP	0.022615289	0.05645225	1.319732663
PPIE_AT	0.251213072	0.120378122	-1.061338317
PPM1M_RI	0.07292755	0.174831435	1.261428782
PPP2R4_AP	0.019798829	0.056398841	1.510250378
PRKAG1_AD	0.009011815	0.141735554	3.975240193
PRKAG2_AP	0.174490475	0.392834538	1.170773492
PRKD2_AP	0.098591143	0.207661402	1.074703138
PRMT2_RI	0.087764816	0.216893693	1.305273506
PRRG1_AT	0.045856735	0.101913632	1.152141518
PTCH1_AP	0.244876036	0.098249518	-1.317529352
PTK2_ES	0.314991739	0.143780183	-1.13144915
PTK2B_RI	0.02055243	0.096244618	2.227396893
PTK7_AP	0.066318853	0.153235943	1.208263783
PTPN3_AP	0.074725427	0.157690948	1.077428701
PTPRC_AT	0.022659991	0.056479912	1.317590576
PTPRJ_AT	0.055663756	0.120399684	1.113021434
PTTG1_AP	0.043148247	0.091790186	1.089037946

PUS7_ES	0.137173101	0.050235215	-1.449226636
R3HDM4_ES	0.028989578	0.066440502	1.196528656
RABEP2_AP	0.163747866	0.34671168	1.082260329
RABEPK_AP	0.180025507	0.451065468	1.325135517
RAC1_ES	0.101457687	0.219257233	1.111746253
RAD21_AP	0.028173015	0.05767972	1.033750227
RAD51B_AT	0.275324922	0.107019627	-1.363259795
RALGDS_AP	0.094390476	0.22288415	1.239580826
RAMP1_AP	0.019695211	0.067546021	1.7780259
RAN_AD	0.037239722	0.087682577	1.235447877
RARRES2_RI	0.035335526	0.081174197	1.199901806
RASAL2_AP	0.133776462	0.051786958	-1.369163577
RASSF5_AP	0.057899476	0.177347637	1.614957914
RASSF7_ES	0.2579302	0.126239487	-1.030817448
RBM26_ES	0.223247916	0.069761969	-1.678134054
RBM47_AP	0.04611139	0.122867139	1.413904047
RBM47_AP	0.123887233	0.287250651	1.213282636
RELA_RI	0.021166097	0.069725161	1.719924108
RFNG_AP	0.034681615	0.101413454	1.548006087
RHBDF1_AT	0.030658457	0.066829823	1.124206945
RHBDF1_RI	0.037339394	0.105358088	1.496530661
RHBDL2_AP	0.071894443	0.169496765	1.23730558
RHOF_AT	0.069212792	0.199072552	1.524183715
RIC8B_AP	0.091106235	0.202103323	1.149471354
RIPK3_AD	0.038295328	0.137613831	1.845385195
RNF8_ES	0.024192886	0.056049388	1.212115746
ROGDI_AD	0.106363631	0.227624933	1.097653668
RPL13_AD	0.041425796	0.186653281	2.171759549
RPL14_AD	0.029739501	0.092612543	1.638827176
RPL26L1_AD	0.032372993	0.070800547	1.128969773
RPL32_RI	0.288567679	0.727753565	1.334540281
RPL32_RI	0.118593176	0.433655253	1.870527582
RPL37A_AT	0.024160276	0.056608907	1.228392107
RPL38_RI	0.02440788	0.056563861	1.212533606
RPL39L_AP	0.47329281	0.22189616	-1.092848301
RPL6_AD	0.086310324	0.217225832	1.331590631
RPP40_AP	0.024702689	0.052788521	1.095556154
RPS2_ES	0.032613066	0.067041596	1.039606392
RPS21_AD	0.211880781	0.500149346	1.239106222
RPS27A_RI	0.051871002	0.130126398	1.326913523
RPS3A_ES	0.112597357	0.353820818	1.651845966
RPS3A_ES	0.203095998	0.453838561	1.160017381
RPS6_AA	0.058093563	0.120795831	1.056120457
RPS6_AD	0.011876742	0.06359784	2.420838607
RSAD1_AD	0.053461605	0.113623742	1.08768926
RTN4_AP	0.604280555	0.223620133	-1.434168437
S100A2_AP	0.375222421	0.129584257	-1.533855584
S100A4_AP	0.049061117	0.23333068	2.249724019
SAA2_AT	0.528578544	0.210579934	-1.327749891
SCO2_AP	0.041859997	0.097082575	1.21364016
SCRN2_AA	0.046288285	0.150271524	1.698852631
SDC3_RI	0.038362998	0.08490141	1.146073046
SEMA4B_AP	0.060213853	0.165522046	1.458856042

SEPT9_AP	0.064640053	0.133434842	1.045635135
SEPT9_AP	0.032049897	0.065712973	1.03585849
SERPINA1_RI	0.444741422	0.894101961	1.007472584
SERPINA1_AA	0.173632379	0.699520639	2.010330618
SERPINA1_AA	0.132253117	0.64471907	2.285368935
SERPINA1_ES	0.007004646	0.070030392	3.321597013
SERPINA1_ES	0.033437565	0.326447498	3.28730927
SERPINB5_AT	0.248309155	0.06742495	-1.880783005
SERPINB6_AP	0.05989002	0.212594438	1.827716344
SESN1_AP	0.708708237	0.339501924	-1.061772064
SESN1_AP	0.287878318	0.646839169	1.167947908
SETD4_AP	0.027224301	0.061848719	1.183848738
SFSWAP_ES	0.037033096	0.076056407	1.038254633
SGK1_AP	0.01848625	0.077228019	2.062671753
SGK1_AP	0.168579264	0.068147158	-1.306701698
SH3BP2_AP	0.523998743	0.228102976	-1.199878084
SH3BP2_AP	0.09984421	0.205609131	1.042153657
SH3BP4_AP	0.213775692	0.435517171	1.026631786
SH3KBP1_AP	0.0838362	0.275543195	1.716633278
SH3YL1_AP	0.051968589	0.105071324	1.015657208
SH3YL1_AP	0.059373374	0.141312952	1.251005707
SHF_AP	0.478605329	0.220573763	-1.117575267
SHROOM1_AA	0.113542938	0.292092218	1.363185945
SIDT2_RI	0.03011186	0.068651382	1.188956945
SIDT2_ES	0.203473734	0.068180742	-1.577406366
SLC12A9_AA	0.023866667	0.088077387	1.883774553
SLC13A3_AT	0.054830326	0.372250241	2.763226839
SLC16A5_AP	0.399009776	0.192505798	-1.051522193
SLC19A1_AP	0.438866456	0.033386364	-3.716451121
SLC22A18_AP	0.015993157	0.131152919	3.035723301
SLC22A18AS_ES	0.038759663	0.202094411	2.38240149
SLC26A6_AP	0.141699266	0.345403727	1.285451362
SLC2A13_AT	0.041169263	0.118874225	1.529796396
SLC37A3_AA	0.038143308	0.089429884	1.229327019
SLC38A10_AT	0.09427245	0.194724056	1.046523
SLC39A13_RI	0.036831708	0.089874383	1.286961655
SLC39A14_ME	0.584757067	0.119120195	-2.295419375
SLC39A14_AP	0.517194959	0.117946969	-2.132569871
SLC3A2_AP	0.093588717	0.210828741	1.171665042
SLC5A6_AP	0.021932627	0.103195516	2.234229755
SLC6A8_AP	0.037418418	0.126105609	1.75281196
SLC8B1_AP	0.053150761	0.152124428	1.517089598
SLCO2B1_AP	0.078158815	0.200604412	1.359872835
SMCO4_AP	0.053033151	0.122814164	1.21151058
SMIM7_ES	0.063009595	0.155300415	1.301418237
SMTN_AP	0.215828385	0.04244987	-2.346052567
SNX5_AT	0.039395286	0.086199766	1.129660947
SOD2_AT	0.013573959	0.080048595	2.560034517
SPATA20_RI	0.047239848	0.119715059	1.341528404
SPIN2A_AP	0.205252754	0.079890933	-1.361297904
SPINK5_AT	0.007322108	0.056012948	2.935429367
SPOP_AP	0.37160768	0.168959391	-1.137103783
SPSB3_AP	0.038829654	0.10511385	1.436722002

SPTB_AT	0.04228102	0.141865455	1.746441241
SRI_AP	0.192391844	0.727654658	1.919206273
SRI_AP	0.807550755	0.272151009	-1.569145551
SRP68_AP	0.025926663	0.093471465	1.850089458
SRPK2_AT	0.204890506	0.096501132	-1.086235358
SRRM1_ES	0.050250075	0.119659756	1.251740362
SSB_ES	0.173121835	0.080789041	-1.099556178
SSBP4_AP	0.13456002	0.051614887	-1.382390695
SSH1_AT	0.035903756	0.085183793	1.246444195
SSH3_RI	0.048060734	0.120787547	1.329541125
ST6GALNAC1_AT	0.025554797	0.088234925	1.787755657
ST6GALNAC1_ES	0.036833664	0.101102107	1.45671624
STAG1_AT	0.031429998	0.076812587	1.289200567
STK25_AP	0.045132302	0.091426119	1.018446026
STK32C_AP	0.032815651	0.076621133	1.223358297
STRIP2_AT	0.075743961	0.198093575	1.386979318
STXBP2_AP	0.050982836	0.143941756	1.497401637
SULT1A1_ES	0.024795699	0.078000764	1.653398379
SULT1A2_RI	0.181779024	0.770388326	2.083400111
SULT1A3_AP	0.756183558	0.376544283	-1.005916938
SULT1A3_AP	0.062174906	0.214134998	1.784116289
SULT1A3_AP	0.175996665	0.399415721	1.182343029
SULT1A3_AA	0.200171413	0.057985254	-1.787477977
SULT1A4_AP	0.03831417	0.112940157	1.559608578
SULT1A4_AP	0.456719757	0.215032212	-1.086756404
SULT2B1_AP	0.545138616	0.146812827	-1.892645103
SUPT4H1_AP	0.10230672	0.306111477	1.581156224
SVIL_ES	0.534783712	0.22731797	-1.23424379
SYNJ2_AT	0.050895434	0.104371021	1.036113047
SYP_AT	0.046913936	0.174005277	1.891042611
SYTL1_AP	0.075120656	0.165369544	1.138411982
SYTL2_ES	0.097697417	0.212287492	1.119627052
TACC1_AP	0.21809343	0.056000428	-1.961436547
TACC2_ES	0.057917479	0.136202311	1.233680475
TADA2A_AD	0.037595469	0.076972176	1.033778255
TAGAP_AT	0.02593339	0.073009312	1.493269663
TAOK2_AP	0.019269316	0.056058501	1.54062776
TAOK3_AP	0.070639753	0.150964117	1.095653483
TAOK3_AP	0.057215448	0.155408436	1.441588187
TBC1D1_ES	0.250494574	0.0423146	-2.565551917
TBC1D14_AP	0.241829385	0.11927132	-1.019742383
TBC1D15_ES	0.382957561	0.120162431	-1.672198619
TBC1D16_AT	0.036432264	0.089102844	1.290254821
TCEB1_AP	0.043624995	0.087528076	1.004590898
TCF12_AP	0.018485321	0.235379025	3.670533738
TCF25_ES	0.028359829	0.067515475	1.251369373
TCF7_ES	0.506690763	0.199817732	-1.342420918
TDP2_AD	0.019637871	0.055428036	1.496977381
TECPR2_AT	0.051306095	0.103157257	1.007643192
TET2_AT	0.051283861	0.111801653	1.124364728
THAP4_AP	0.283071255	0.624321269	1.14112336
THOP1_AP	0.066226174	0.167701298	1.340420444
TINAGL1_RI	0.052514393	0.144638143	1.461663268

TMCC1_AP	0.003524749	0.071316586	4.338645088
TMCO1_AP	0.02758531	0.06075448	1.139090636
TMCO6_RI	0.101798436	0.218941944	1.104832971
TMEM175_ES	0.196841157	0.406019201	1.044516051
TMEM2_AP	0.031209064	0.070282668	1.171203859
TMEM205_ES	0.105934204	0.2199887	1.054260941
TNC_ES	0.06042226	0.166030237	1.458293954
TNC_ES	0.115768372	0.445397639	1.943852746
TNC_ES	0.023063197	0.166829844	2.854712997
TNFAIP8_AP	0.274789167	0.064720489	-2.08603071
TNFRSF10C_AT	0.069820679	0.364887348	2.38572483
TNIP1_AP	0.183569259	0.429384933	1.225947091
TNPO2_AP	0.114728669	0.049798353	-1.204056016
TOR2A_RI	0.05180904	0.104283595	1.009236461
TP53I3_RI	0.141261816	0.340404519	1.268878643
TPM1_ES	0.014194318	0.098925357	2.80102687
TPM1_AA	0.032967076	0.1678445	2.348027447
TRABD_AP	0.41842322	0.165590647	-1.337341728
TRIM11_AT	0.023863995	0.056701144	1.248542256
TRNT1_AD	0.026630675	0.071981148	1.434530108
TRPM6_AT	0.197000378	0.41489655	1.074553261
TSC22D1_AP	0.019627634	0.056941914	1.536604704
TSPAN17_RI	0.031469981	0.087706887	1.478713828
TSPAN31_AT	0.025991175	0.068019051	1.387916991
TSPAN8_AP	0.185172187	0.427759346	1.207931958
TTC31_RI	0.102507483	0.209727254	1.032785127
TTC39C_AP	0.074811493	0.150414959	1.007616225
TTC7A_AP	0.159347444	0.331404147	1.05641578
TTLL12_AP	0.800619632	0.194579162	-2.040759775
TTLL12_AP	0.198578106	0.804725992	2.01879106
TUBB3_AP	0.149420364	0.070486761	-1.083952557
TUBB3_ES	0.164947078	0.056600296	-1.543121705
TUBGCP3_AT	0.016419211	0.054799573	1.738779866
TXNDC9_AT	0.043794076	0.09547809	1.12443398
UBA1_AP	0.105673728	0.213866267	1.017092197
UBAP2_AP	0.13721893	0.393190587	1.518749268
UBE2D3_AP	0.018068706	0.056714364	1.650220961
UBE2Z_AP	0.065960453	0.141342163	1.099518688
UBE3D_AT	0.106529835	0.05031936	-1.082072043
UBR4_AP	0.2999855	0.135429648	-1.147349167
UBR4_AP	0.169828106	0.456272464	1.42582035
UGDH_AP	0.025362238	0.062182234	1.293820364
UGP2_AP	0.354505265	0.772033429	1.122856263
UGP2_AP	0.643769569	0.224309291	-1.521055004
UGT1A1_AT	0.072007021	0.15543042	1.110059407
UGT1A10_AT	0.036265155	0.080843803	1.156553171
URGCP_RI	0.03523947	0.085657467	1.281386794
USP1_AP	0.201287732	0.08613457	-1.22459497
VAMP2_RI	0.074038185	0.173084424	1.225134468
VCL_ES	0.512015159	0.138217139	-1.889250006
VPS16_AP	0.018405834	0.067471052	1.874105574
VPS16_RI	0.045637431	0.103956361	1.187688538
VPS28_RI	0.042822208	0.119842936	1.484713773

VPS28_RI	0.015428278	0.054652555	1.824711894
VPS28_RI	0.019499694	0.061443816	1.655816328
VPS9D1_AP	0.327405023	0.711316507	1.119415186
VPS9D1_AP	0.672392984	0.288284907	-1.221809365
VSNL1_AP	0.149595985	0.055449677	-1.431820489
VWA2_AT	0.252601818	0.089304914	-1.500053562
WAC_ES	0.020473678	0.051099153	1.319529101
WDR6_AA	0.127432509	0.256263457	1.007894403
WDR90_AP	0.207489375	0.095893508	-1.1135324
WSB2_AP	0.496315718	0.133670813	-1.892573661
XPA_ES	0.035239523	0.079533762	1.174373018
XPNPEP2_AT	0.028029224	0.096498866	1.783580181
YME1L1_AA	0.020421588	0.066354748	1.700104613
ZBTB45_AD	0.041559145	0.108135811	1.379606486
ZDHHC4_ES	0.083927376	0.193784534	1.207240046
ZFAND2B_RI	0.052294654	0.109564816	1.067049216
ZFP30_AT	0.239060055	0.086972356	-1.45874427
ZFYVE28_AT	0.026737123	0.099009397	1.888721241
ZMYND11_AP	0.07077539	0.147444988	1.058857083
ZMYND11_AP	0.021669101	0.054230268	1.323459031
ZNF283_AT	0.062482169	0.13808017	1.143989715
ZNF283_AT	0.168822633	0.338323565	1.002895337
ZNF493_AT	0.071968524	0.148637698	1.046362089
ZNF66_AT	0.12719206	0.292372295	1.200797996
ZNF670_AT	0.09997103	0.243059932	1.281730093
ZNF76_RI	0.056510533	0.124243401	1.136577528
ZNF76_RI	0.041200949	0.106199048	1.366021359
ZNF771_AT	0.299230736	0.097865403	-1.612387532
ZNF814_AT	0.136472629	0.281476475	1.044402719
ZNRF1_AT	0.0252812	0.07064611	1.482545175
ZWINT_RI	0.059744786	0.134445975	1.170141856

ent prevalence between normal and tumor samples

pValue
3.40042E-21
7.91613E-18
2.3671E-16
1.20787E-17
9.84363E-15
5.1072E-14
5.05406E-20
1.1788E-22
2.21254E-08
2.40321E-27
7.11291E-09
3.40164E-23
1.68838E-13
1.51424E-29
5.47893E-19
1.37032E-09
3.5829E-07
1.44136E-14
5.05225E-22
1.62888E-20
0.009498616
2.45559E-15
0.000217178
4.52033E-24
1.42961E-10
1.82606E-29
0.00246076
1.13256E-15
8.33267E-12
5.18456E-19
9.1023E-17
0.01208274
4.76572E-07
1.16445E-12
7.32934E-17
7.6954E-28
4.49703E-07
2.15293E-11
6.22889E-25
1.02845E-19
1.25868E-07
6.40883E-20
2.93975E-13
0.007427759
9.62217E-07
2.08364E-16
1.67127E-16
4.26445E-23
0.000123439
5.01666E-10

5.0021E-25
1.05763E-17
5.79949E-06
3.36273E-23
1.15767E-24
1.4235E-09
3.03118E-09
7.89175E-06
0.000921228
7.13975E-23
7.0118E-13
9.41757E-20
7.83406E-08
5.05903E-26
2.38788E-22
0.004997281
1.09539E-26
7.47549E-16
1.02405E-06
3.30862E-18
3.07319E-15
1.78493E-17
3.53266E-26
6.90974E-12
1.25157E-11
3.16319E-10
8.95742E-06
3.20141E-13
2.39311E-17
7.44775E-12
2.11059E-24
1.87356E-19
0.000871394
2.44718E-11
2.87653E-06
8.66796E-09
5.46043E-11
2.42813E-11
1.03919E-14
5.79305E-07
2.09306E-14
2.57835E-11
2.78689E-09
1.56905E-09
9.3516E-20
1.07745E-15
6.94899E-05
5.94247E-11
8.47594E-26
2.47936E-14
1.86729E-12
7.14829E-14
5.62333E-06

6.25023E-14
1.36462E-27
2.24226E-16
7.27158E-13
3.13984E-15
1.28343E-21
6.00356E-07
1.96329E-22
4.12894E-06
0.000242364
2.35374E-13
6.56956E-20
6.07726E-20
6.32461E-24
7.0246E-05
9.12101E-26
1.5393E-24
3.51566E-13
1.67722E-12
2.26286E-17
2.76524E-17
1.32421E-06
4.91614E-14
6.01195E-24
7.46754E-14
2.87339E-21
3.76603E-25
3.26753E-15
1.80018E-27
1.01652E-21
8.02903E-21
6.65077E-11
1.13113E-07
5.76517E-17
1.22184E-19
4.78484E-13
3.20074E-22
4.29933E-06
9.13246E-27
1.37169E-21
2.12737E-24
2.06531E-26
2.07067E-08
4.03641E-18
1.51033E-20
3.27335E-22
5.06201E-17
2.24958E-29
1.99231E-09
7.19403E-09
0.000572533
7.46579E-14
1.20907E-15

1.97363E-09
3.09339E-31
8.69604E-10
7.06885E-32
2.71994E-18
1.99015E-11
4.16014E-22
1.89742E-21
3.75241E-13
0.000165687
7.19239E-19
8.62516E-11
4.83102E-15
9.13483E-31
9.38179E-31
7.88586E-13
4.23255E-12
1.82066E-15
3.25771E-17
1.0201E-14
5.03174E-16
3.15401E-07
1.16788E-31
1.492E-13
7.42791E-12
1.6543E-12
2.60967E-08
5.03167E-16
2.13573E-08
1.88012E-19
1.12631E-12
7.96968E-15
0.00013686
8.0871E-21
7.81952E-17
3.76272E-20
1.15267E-06
1.21215E-07
2.06254E-25
4.34704E-13
1.34859E-12
5.74228E-20
2.4908E-09
4.08066E-09
1.18767E-09
1.13052E-24
4.75803E-15
1.20975E-06
3.64451E-24
1.68002E-18
2.94306E-17
4.15676E-20
1.8796E-18

1.58405E-21
1.98557E-22
1.29729E-09
5.95595E-07
1.02955E-26
1.24324E-24
8.66033E-16
4.01382E-14
7.24571E-05
5.97757E-12
5.50826E-13
8.17836E-12
2.0037E-15
1.11927E-21
5.11157E-17
0.000172415
9.26366E-22
1.37361E-09
4.53815E-09
5.29578E-06
6.85717E-18
1.46553E-16
6.57426E-13
8.4749E-30
1.25598E-07
3.78759E-05
4.96498E-24
6.89461E-21
5.3368E-14
1.5424E-26
5.50805E-27
2.01923E-10
2.45193E-25
2.81727E-19
3.41048E-17
2.48546E-19
1.01246E-16
1.4083E-22
5.18706E-11
3.33382E-12
3.73132E-05
8.09772E-09
3.34432E-11
3.37564E-21
5.76447E-32
2.67422E-11
4.60646E-16
1.01049E-19
8.67297E-15
3.07387E-26
1.49793E-12
9.7518E-16
2.14413E-24

1.32834E-23
1.65927E-24
1.78196E-12
1.27693E-06
2.3213E-10
1.42908E-12
1.03294E-14
4.82539E-25
1.04078E-29
5.30715E-21
6.74339E-18
3.67577E-10
7.08285E-15
1.96731E-16
2.1784E-20
1.32313E-15
0.000440178
6.6543E-07
3.0431E-16
5.36839E-20
2.74323E-15
8.47291E-16
0.000607547
4.63956E-10
2.89058E-18
1.60165E-21
2.86143E-08
9.82813E-28
3.49142E-12
1.33342E-13
7.17733E-16
4.15567E-10
1.44455E-07
7.33456E-08
1.3029E-23
2.07004E-06
2.78558E-15
3.22046E-18
7.54063E-20
5.74273E-18
1.00683E-11
1.195E-24
6.232E-14
1.99898E-16
1.37915E-17
2.94753E-09
2.02854E-13
8.62871E-19
2.80881E-13
4.24428E-05
3.89912E-20
9.06961E-09
2.81647E-10

2.04736E-30
1.07752E-15
6.89525E-08
5.17214E-07
0.000177764
2.74712E-17
1.12755E-21
1.11406E-10
4.44671E-20
4.37685E-19
1.5581E-05
5.74272E-18
1.62929E-20
1.10935E-22
4.52791E-14
7.33655E-16
8.06677E-15
7.25322E-14
6.07876E-08
1.40849E-13
1.40956E-12
4.4998E-22
6.53714E-09
1.59003E-17
1.17314E-31
9.88109E-32
3.64852E-18
3.12463E-26
5.95316E-22
2.42688E-08
1.44683E-16
2.01229E-28
1.6393E-28
1.20789E-27
3.88953E-05
1.51228E-25
1.56729E-14
0.001357778
2.46825E-19
0.002371851
4.11255E-25
2.02905E-24
2.83853E-22
3.90226E-12
1.87373E-10
3.55839E-27
5.85042E-12
3.83335E-09
5.14463E-20
1.79831E-15
2.64637E-25
1.09673E-18
8.15707E-23

5.84995E-19
6.94469E-24
0.000329482
7.54277E-10
1.38641E-19
4.01402E-19
3.46385E-14
1.79832E-15
1.49322E-21
1.91175E-18
1.14792E-10
6.91402E-10
2.54702E-07
1.6865E-12
5.1524E-14
1.55656E-23
2.75617E-17
6.27109E-07
3.50987E-16
6.466E-07
8.26357E-16
1.55254E-16
4.4715E-13
5.20251E-19
1.05261E-05
8.17148E-31
1.78232E-21
1.0086E-08
5.38387E-14
3.56164E-26
6.17199E-17
3.55708E-14
1.16536E-26
7.13546E-12
2.98317E-11
4.93154E-23
1.21639E-09
2.39317E-27
1.38772E-16
1.33099E-16
4.53365E-22
0.000236619
5.33429E-18
1.0613E-14
1.01453E-10
3.80589E-13
1.76733E-17
0.000313405
2.58777E-16
0.002799695
6.68859E-31
1.874E-05
7.18924E-06

1.41943E-06
3.54404E-08
9.6779E-17
2.95565E-07
3.42733E-27
8.44453E-22
1.71936E-14
6.19218E-17
3.17061E-19
5.2919E-05
2.1653E-22
2.05307E-18
2.42559E-19
1.08902E-10
4.49951E-24
7.27074E-08
1.85263E-07
1.34114E-20
5.93836E-18
1.57316E-07
1.95123E-12
2.79289E-13
2.49812E-22
1.80934E-06
2.97367E-12
9.78207E-16
1.53957E-09
1.33491E-09
3.31149E-17
2.01115E-11
1.63586E-13
1.9907E-08
1.03669E-11
8.99553E-28
2.39677E-28
2.96153E-14
1.08036E-19
2.52088E-23
2.64144E-24
2.35211E-16
9.1682E-13
4.18637E-10
7.10718E-11
1.12009E-12
6.62862E-17
2.03887E-12
4.55042E-26
3.28195E-26
6.55576E-13
9.02703E-12
2.02537E-08
7.61959E-17
3.90422E-13

5.01965E-07
5.94871E-10
9.97097E-32
1.04212E-06
3.55837E-12
9.92491E-10
6.47905E-12
2.21034E-28
1.00908E-22
2.14375E-10
9.43097E-17
2.22302E-13
1.03918E-14
5.92369E-23
1.51419E-09
2.13123E-18
2.5572E-10
1.8034E-26
3.55543E-05
4.22762E-10
8.64214E-07
1.49708E-05
3.72073E-13
5.9856E-25
2.66474E-19
8.47307E-11
8.7584E-26
4.05541E-31
2.73839E-18
2.15469E-08
6.26721E-19
1.36455E-13
1.52673E-06
3.76202E-17
1.08013E-20
1.14853E-20
3.32569E-05
3.67085E-29
2.18969E-17
2.72508E-12
3.25195E-29
5.45514E-12
2.42477E-17
4.02916E-17
1.09453E-10
3.40538E-20
1.39724E-21
1.09184E-19
4.60671E-08
3.3271E-08
5.08027E-09
1.62373E-15
1.9211E-10

1.22766E-21
1.34072E-11
2.57146E-13
6.34302E-27
2.0802E-21
0.001399894
6.32086E-13
2.33438E-19
7.94554E-15
0.000588965
4.40021E-17
0.001787047
1.51391E-11
9.46572E-15
2.13844E-18
7.09746E-12
9.16171E-25
3.18275E-25
8.76168E-07
2.75343E-13
2.45575E-15
3.46764E-08
3.7349E-29
4.20974E-12
6.8697E-21
3.65112E-09
2.06003E-10
1.46421E-25
2.95302E-15
8.43013E-11
3.24104E-28
6.21556E-29
1.73035E-08
6.20138E-06
3.4958E-13
5.2495E-21
4.32894E-07
0.000160915
2.32586E-22
4.79256E-21
2.4256E-19
5.47948E-18
2.02091E-08
7.47266E-17
3.15873E-11
1.37802E-24
7.9791E-20
6.20256E-24
9.29601E-15
0.003277402
4.04191E-19
1.79274E-15
5.63952E-08

1.60853E-11
2.90298E-05
1.03109E-28
6.7462E-28
2.05838E-27
2.03596E-07
5.33198E-25
7.83963E-22
6.83434E-18
3.07885E-24
4.0887E-23
4.38881E-05
3.05963E-09
3.10375E-20
7.57529E-11
7.30542E-24
3.97647E-08
8.01203E-11
1.00296E-15
2.08353E-09
1.08337E-16
9.86313E-19
9.3369E-19
9.48237E-10
1.37799E-24
4.67435E-20
4.48809E-21
3.12693E-19
2.68352E-29
3.33121E-10
1.67566E-22
5.46391E-21
4.20345E-16
1.18727E-12
1.6858E-18
1.45803E-13
1.30736E-31
7.91675E-32
2.41504E-22
1.93863E-11
9.77225E-09
2.72227E-13
2.18441E-07
4.21331E-06
1.79578E-09
1.14798E-16
1.26287E-15
1.35218E-15
1.16147E-14
9.01611E-13
2.50363E-12
1.64405E-14
1.17517E-06

1.93334E-08
3.9677E-30
2.92788E-30
0.000277596
1.70578E-24
3.64298E-11
1.36008E-18
7.34234E-19
2.86612E-09
3.70401E-16
9.41389E-18
1.82572E-09
8.2672E-06
1.2121E-06
3.55563E-06
7.21149E-17
2.35145E-18
7.21102E-14
1.58629E-24
9.03368E-28
3.25634E-28
5.69277E-22
1.32055E-22
2.08756E-25
1.27083E-26
3.93264E-27
5.69277E-22
7.99167E-11
1.83494E-13
7.6318E-21
1.32579E-10
1.27279E-09
3.26016E-25
1.31812E-13
1.83726E-08
0.003447311
1.99894E-16
1.55921E-06
1.29924E-13
1.89929E-16
2.64101E-17
5.88208E-26
7.57544E-11
5.29583E-06
3.24715E-13
5.85125E-15
1.51838E-25
5.60753E-15
4.28365E-09
5.07223E-15
1.74993E-18
9.93314E-15
1.16897E-23

1.21501E-22
4.33103E-06
1.5833E-11
9.72109E-20
0.000885863
2.2623E-09
5.16896E-12
5.02674E-23
2.0049E-18
1.90085E-27
1.14441E-20
1.5015E-18
1.44594E-19
2.07928E-15
3.76059E-24
2.84617E-15
7.57668E-21
1.82416E-24
2.96297E-20
2.18517E-13
2.33343E-05
1.62721E-17
9.89694E-19
4.15569E-10
1.23913E-19
5.56015E-14
5.04147E-06
2.69911E-13
1.48331E-31
2.61327E-31
3.5308E-19
2.6383E-18
2.46985E-12
2.17098E-07
2.71967E-21
2.33715E-16
2.73777E-13
6.64968E-08
6.89912E-23
3.02118E-17
1.09065E-21
3.78282E-08
4.75737E-29
3.63928E-29
6.07474E-12
7.76888E-17
1.77769E-11
7.71883E-17
9.29989E-22
3.31298E-25
3.84928E-13
2.86232E-12
4.15139E-27

5.86731E-25
1.93524E-24
1.15897E-28
3.71483E-28
4.95295E-16
9.48423E-20
1.36938E-18
4.78938E-17
0.000157502
6.72218E-27
1.33375E-12
2.84265E-08
2.70967E-21
1.79182E-12
1.34501E-13
7.88453E-16
1.44543E-14
1.92659E-13
6.71875E-09
1.19646E-11
7.42228E-05
0.00493237
1.01024E-13
0.002332975
3.53219E-16
2.71809E-14
3.14126E-16
1.06917E-27
1.77407E-18
6.57902E-08
3.37311E-14
