# Supplementary material for: CLK2 Expression Is Associated with the Progression of Colorectal Cancer and Is a Prognostic Biomarker
Source: Biomed Res Int. 2022 Jul 7;2022:7250127. doi: 10.1155/2022/7250127 (PMC9289758; doi:10.1155/2022/7250127)
Supplement: Supplementary 3 — Table S3: the relationship between CLK2 expression and PSI value of differential splicing genes. [file 7250127.f3.pdf]

Supporting Information Table S3 The relationship bet

AS event	Gene	P_value
AACS_AP	CLK2	0
AACS_RI	CLK2	8.10834E-11
AAK1_AT	CLK2	3.56233E-16
AAMDC_AT	CLK2	3.74618E-05
AAR2_AD	CLK2	0.008020468
ABCB8_AD	CLK2	1.10402E-15
ABCB8_RI	CLK2	4.19418E-15
ABCB8_RI	CLK2	1.0974E-17
ABCB9_AP	CLK2	9.36896E-05
ABCB9_AT	CLK2	0.000147759
ABCC3_RI	CLK2	0
ABCC3_AA	CLK2	1.37717E-18
ABCC5_AT	CLK2	0.0001873
ABCC5_RI	CLK2	1.95207E-17
ABCC5_RI	CLK2	2.43508E-11
ABCC6_AT	CLK2	3.16129E-14
ABCD1_AT	CLK2	0.001163425
ABCD3_AT	CLK2	2.93223E-05
ABCD4_RI	CLK2	6.48727E-05
ABCD4_AA	CLK2	0
ABCD4_AD	CLK2	0
ABHD11_ES	CLK2	0.003782062
ABHD11_ES	CLK2	0.001021039
ABHD14B_AA	CLK2	0.000299453
ABHD17A_AP	CLK2	5.89093E-15
ABHD17A_ES	CLK2	2.78108E-08
ABHD17A_AD	CLK2	0
ABHD3_AD	CLK2	0
ABHD3_ES	CLK2	4.19647E-09
ABI1_ES	CLK2	8.88671E-05
ABTB1_AP	CLK2	0
ABTB1_RI	CLK2	0
ACAA1_AD	CLK2	0
ACAA1_RI	CLK2	0
ACACA_AT	CLK2	0.000765331
ACAD10_AT	CLK2	2.50857E-13
ACAD8_AT	CLK2	0.001975118
ACADVL_RI	CLK2	1.11783E-06
ACAT2_AP	CLK2	5.50268E-06
ACBD5_RI	CLK2	0.011463781
ACCS_AT	CLK2	4.87049E-07
ACHE_RI	CLK2	5.5845E-16
ACIN1_AP	CLK2	7.56701E-07
ACIN1_AD	CLK2	0.000153679
ACIN1_RI	CLK2	0.000467553
ACLY_ES	CLK2	5.28505E-06
ACOT11_AT	CLK2	5.088E-13
ACOT2_AP	CLK2	1.36419E-05
ACOT7_AP	CLK2	1.42602E-12
ACOT8_AA	CLK2	0

ACP1_RI	CLK2	0
ACP1_AA	CLK2	2.86823E-13
ACP5_AP	CLK2	0.000128436
ACPL2_AP	CLK2	2.28551E-12
ACPL2_AP	CLK2	1.45836E-06
ACSF2_ES	CLK2	0
ACSL5_AP	CLK2	2.78839E-07
ACSM3_AT	CLK2	0.001278208
ACSS2_AP	CLK2	1.83685E-07
ACTG1_RI	CLK2	2.19978E-10
ACTG1_RI	CLK2	0
ACTR10_ES	CLK2	2.3488E-07
ACYP2_AT	CLK2	4.9236E-07
ADAM15_AD	CLK2	0
ADAM15_ES	CLK2	2.1013E-17
ADAM15_ES	CLK2	0
ADAM15_ES	CLK2	0
ADAM15_ES	CLK2	0
ADAM15_ES	CLK2	0.008378584
ADAM15_ES	CLK2	9.08682E-07
ADAM17_AT	CLK2	0.006592161
ADAMTS12_AT	CLK2	0.000476885
ADAMTS2_AT	CLK2	0.000339092
ADAP1_AP	CLK2	3.58102E-06
ADAP1_AP	CLK2	5.82314E-13
ADARB1_RI	CLK2	0.002567428
ADAT2_AP	CLK2	1.02938E-15
ADCK3_AP	CLK2	2.54307E-05
ADCK5_RI	CLK2	0
ADCK5_RI	CLK2	0
ADCK5_ES	CLK2	1.56304E-16
ADCY6_AP	CLK2	1.39487E-07
ADD1_ES	CLK2	0.000256841
ADD3_ES	CLK2	0.003745034
ADK_AP	CLK2	3.37572E-07
ADM_RI	CLK2	2.33766E-16
ADM_RI	CLK2	1.34072E-14
ADNP_AP	CLK2	7.05036E-09
ADNP_AP	CLK2	2.34417E-13
ADNP_ES	CLK2	0.001600048
ADPRM_AD	CLK2	0.000212784
AFF4_AT	CLK2	0.001033728
AFMID_AT	CLK2	0.000151037
AFMID_ES	CLK2	3.0952E-07
AFTPH_ES	CLK2	1.64063E-05
AGAP3_AP	CLK2	0.008589272
AGAP3_AT	CLK2	1.89499E-06
AGAP3_ES	CLK2	0.002084154
AGAP8_AP	CLK2	8.2614E-17
AGO3_AT	CLK2	0.013184853
AHCYL1_AP	CLK2	0.001255214
AHCYL2_AP	CLK2	3.07916E-12
AHI1_AT	CLK2	1.4913E-10

AHNAK_AT	CLK2	0.000175986
AIDA_AP	CLK2	0.004545802
AIFM1_AP	CLK2	0.000225805
AIG1_AT	CLK2	0.011175282
AIG1_AT	CLK2	1.34427E-07
AIMP1_AP	CLK2	1.33215E-05
AK7_AT	CLK2	2.47507E-07
AKAP1_ES	CLK2	1.15843E-16
AKAP17A_AT	CLK2	0
AKAP17A_RI	CLK2	7.31168E-15
AKIP1_RI	CLK2	1.15607E-05
AKNA_AT	CLK2	0.003309125
AKR1A1_AT	CLK2	0
AKR1C2_AT	CLK2	8.78528E-05
AKT1_AP	CLK2	0
AKT1_RI	CLK2	2.07739E-16
AKT1_AD	CLK2	0.001967341
AKT1S1_AP	CLK2	0
AKT1S1_AP	CLK2	2.33454E-08
AKT3_AT	CLK2	2.21366E-08
ALAS1_ES	CLK2	0.004033285
ALDH18A1_AD	CLK2	1.6263E-10
ALDH3A2_ES	CLK2	0
ALG13_AT	CLK2	0
ALG2_ES	CLK2	8.87123E-06
ALKBH6_AD	CLK2	1.66451E-08
ALS2_AT	CLK2	1.7112E-08
ALS2CL_RI	CLK2	0
ALS2CL_RI	CLK2	0
ALS2CL_AA	CLK2	0
ALS2CL_RI	CLK2	0
ALS2CL_RI	CLK2	0
AMACR_RI	CLK2	0.000115411
AMZ2_RI	CLK2	0.002389408
ANAPC10_ES	CLK2	0.000450569
ANAPC11_ES	CLK2	0.014132572
ANAPC11_AD	CLK2	0.005790662
ANAPC11_ES	CLK2	0.015234496
ANAPC13_AD	CLK2	0.000772436
ANAPC15_AT	CLK2	1.2091E-06
ANAPC7_AT	CLK2	6.24443E-05
ANGEL1_AP	CLK2	3.70018E-11
ANGEL2_ES	CLK2	0.048861653
ANKDD1A_ES	CLK2	0.00021472
ANKH_AT	CLK2	0.004266673
ANKHD1_ES	CLK2	0.003208125
ANKLE2_AP	CLK2	2.55092E-17
ANKMY1_AT	CLK2	0.000196012
ANKRD10_AT	CLK2	8.08328E-05
ANKRD10_ES	CLK2	1.48509E-10
ANKRD10_ES	CLK2	6.4157E-11
ANKRD11_AT	CLK2	1.77692E-07
ANKRD13D_AP	CLK2	2.69823E-12

ANKRD13D_ES	CLK2	9.95129E-06
ANKRD17_ES	CLK2	0.010770369
ANKRD29_AT	CLK2	5.34991E-05
ANKRD39_AT	CLK2	0
ANKRD42_AT	CLK2	0.00026017
ANKRD46_AD	CLK2	5.2323E-17
ANKRD49_RI	CLK2	6.54255E-16
ANKRD49_AD	CLK2	0.000160303
ANKRD9_RI	CLK2	1.28081E-12
ANKRD9_AA	CLK2	2.13459E-15
ANKRD9_ES	CLK2	0.001941061
ANKS3_ES	CLK2	8.77249E-09
ANKS6_RI	CLK2	0.008353488
ANO6_AT	CLK2	0.0019168
ANO7_AT	CLK2	1.18931E-05
ANTXR1_AT	CLK2	3.36512E-06
ANXA13_ES	CLK2	1.3398E-08
AOAH_ES	CLK2	4.12806E-13
AP1G1_AP	CLK2	6.12976E-05
AP1G2_RI	CLK2	9.02965E-11
AP1G2_AD	CLK2	4.6442E-09
AP1S2_AA	CLK2	3.49162E-05
AP1S3_AT	CLK2	0.010469506
AP4B1_AT	CLK2	0
AP4M1_AP	CLK2	3.41214E-05
APBB2_ES	CLK2	0.001005701
APBB3_RI	CLK2	2.89605E-16
APEH_ES	CLK2	3.92182E-05
APEX1_AD	CLK2	4.91215E-05
APIP_AT	CLK2	3.87913E-08
APLP2_ES	CLK2	2.48985E-12
APLP2_ES	CLK2	0.012875369
APMAP_ES	CLK2	0.014726889
APOBEC3B_RI	CLK2	0.002718381
APOBEC3F_AT	CLK2	0.003015721
APOC1_ES	CLK2	0.001096236
APOL1_AA	CLK2	0.006198817
APOL2_ES	CLK2	9.24433E-06
APPL2_AP	CLK2	6.40523E-15
APRT_AA	CLK2	0.002126593
APTX_AP	CLK2	2.48468E-09
APTX_AA	CLK2	3.39704E-05
AQP1_AP	CLK2	1.61881E-05
ARAP1_AP	CLK2	0
ARAP1_AP	CLK2	1.31968E-05
ARFGAP1_ES	CLK2	0.005948225
ARFGAP1_AA	CLK2	0.013155932
ARFGAP1_ES	CLK2	0
ARFGAP2_AA	CLK2	2.93488E-05
ARFIP1_AD	CLK2	1.34847E-05
ARFIP2_ES	CLK2	0
ARFRP1_AP	CLK2	1.32432E-13
ARFRP1_RI	CLK2	1.87077E-15

ARHGAP11A_AT	CLK2	0.002419345
ARHGAP26_AD	CLK2	0.002954539
ARHGAP32_RI	CLK2	4.35622E-11
ARHGAP32_RI	CLK2	0.000640308
ARHGAP8_ES	CLK2	1.15729E-06
ARHGEF1_AP	CLK2	0.001281233
ARHGEF1_ES	CLK2	1.48534E-16
ARHGEF16_AP	CLK2	3.24631E-11
ARHGEF16_AP	CLK2	1.15933E-13
ARID5A_ES	CLK2	3.72815E-08
ARIH2_AP	CLK2	0
ARIH2_ES	CLK2	2.60808E-06
ARL16_AP	CLK2	0.010647004
ARL16_ES	CLK2	0
ARL16_ES	CLK2	2.06668E-12
ARL17B_AT	CLK2	9.91744E-06
ARL6IP4_RI	CLK2	0
ARL6IP4_AD	CLK2	4.00033E-05
ARL6IP4_AD	CLK2	2.50296E-05
ARL6IP4_AD	CLK2	4.27477E-05
ARMC6_AD	CLK2	2.05439E-05
ARMCX3_AP	CLK2	0.000216089
ARMCX5_RI	CLK2	3.18007E-15
ARMCX5_AD	CLK2	1.44837E-15
ARPC1A_AT	CLK2	0.00275573
ARRB2_AP	CLK2	0
ARRB2_ES	CLK2	9.95943E-13
ARRB2_ES	CLK2	1.86921E-05
ARRDC1_RI	CLK2	0
ARRDC2_AP	CLK2	2.85506E-10
ARSA_RI	CLK2	2.6201E-07
ARSA_RI	CLK2	6.99433E-07
ARSE_AP	CLK2	0.004850083
ARSK_AT	CLK2	0.000193737
ASAH1_AP	CLK2	2.41724E-07
ASCC1_AP	CLK2	1.04083E-15
ASCC2_ES	CLK2	0.000159335
ASCC3_AT	CLK2	0.00468024
ASGR1_AT	CLK2	0.031620403
ASL_ES	CLK2	0.004149135
ASNS_AP	CLK2	1.50308E-08
ASNS_AA	CLK2	0
ASNS_ES	CLK2	0.003493879
ASPH_AT	CLK2	0.001030315
ASPSCR1_AT	CLK2	4.88004E-10
ASPSCR1_ES	CLK2	0
ASS1_ME	CLK2	2.7714E-07
ASTN2_AT	CLK2	3.34781E-13
ASXL1_AP	CLK2	2.64446E-08
ATAD3A_AP	CLK2	3.66177E-06
ATAD3A_AA	CLK2	0
ATE1_AP	CLK2	1.78876E-05
ATF3_AA	CLK2	0

ATF5_AP	CLK2	0.008468324
ATF5_AT	CLK2	2.49103E-05
ATF7_AP	CLK2	0.000342344
ATF7_AT	CLK2	3.77591E-09
ATG10_ES	CLK2	0.016290449
ATG12_AD	CLK2	7.6645E-13
ATG16L2_AT	CLK2	0.008509248
ATG16L2_AD	CLK2	0
ATG16L2_RI	CLK2	0
ATG16L2_AA	CLK2	0.000262069
ATG16L2_AA	CLK2	0
ATG4B_AD	CLK2	0
ATG5_AP	CLK2	0.000382644
ATG9A_AP	CLK2	1.12265E-05
ATHL1_AA	CLK2	5.39505E-12
ATMIN_AP	CLK2	2.9898E-07
ATMIN_AP	CLK2	4.17094E-13
ATP13A2_ES	CLK2	0.000387413
ATP2A2_RI	CLK2	0.002388559
ATP5G3_RI	CLK2	0.000364673
ATP5SL_AP	CLK2	0.000112273
ATP5SL_AT	CLK2	0.000208873
ATP6V0A2_AP	CLK2	5.69606E-16
ATP6V0B_ES	CLK2	0
ATP6V0B_AD	CLK2	8.37182E-13
ATP6V0D1_AP	CLK2	1.65276E-08
ATP6V1D_AT	CLK2	1.24344E-11
ATP6V1E2_AP	CLK2	0.003169151
ATP8B2_AT	CLK2	3.54195E-05
ATP9B_AT	CLK2	1.28664E-17
ATPAF2_AT	CLK2	0.000391564
ATRN_AT	CLK2	0.004839055
ATXN2L_RI	CLK2	0.001274104
ATXN2L_RI	CLK2	1.13682E-13
ATXN2L_RI	CLK2	1.7648E-08
ATXN2L_RI	CLK2	3.8983E-13
ATXN2L_ES	CLK2	0.007221631
ATXN2L_RI	CLK2	3.20729E-17
ATXN2L_AD	CLK2	1.54613E-06
ATXN7L1_AT	CLK2	4.84475E-14
AUH_AT	CLK2	2.7251E-14
AUP1_AA	CLK2	0
AURKA_ES	CLK2	0.000355305
AURKA_ES	CLK2	0.001357532
AURKA_AD	CLK2	0.006143728
AURKAIP1_RI	CLK2	4.57949E-05
AURKAIP1_AD	CLK2	0.013444288
AXIN2_ES	CLK2	2.27846E-05
AXL_AP	CLK2	0.000191943
AZGP1_RI	CLK2	0
B2M_AT	CLK2	1.35938E-09
B3GAT3_ES	CLK2	0
B3GNT5_AP	CLK2	1.79214E-06

B4GALT2_AP	CLK2	4.28372E-06
B4GALT3_AA	CLK2	5.68041E-05
B4GALT4_AP	CLK2	0.000853743
B4GALT4_ES	CLK2	1.85472E-05
B4GALT4_ES	CLK2	0.000976115
B9D1_RI	CLK2	0
BABAM1_AA	CLK2	1.5386E-09
BAD_RI	CLK2	1.72042E-06
BAG1_ES	CLK2	5.17943E-07
BAIAP2_AT	CLK2	0.008617785
BAIAP2_ES	CLK2	0.015306874
BBC3_AP	CLK2	0.009317333
BBS1_RI	CLK2	0
BBS1_ES	CLK2	0
BBS5_AT	CLK2	3.87042E-05
BCAM_AT	CLK2	7.58819E-07
BCAR3_AP	CLK2	1.02849E-07
BCAR3_AP	CLK2	2.00501E-08
BCAS1_ES	CLK2	2.32091E-14
BCAT2_ES	CLK2	0.000505421
BCAT2_ES	CLK2	7.1316E-16
BCKDHB_RI	CLK2	1.35934E-08
BCKDK_RI	CLK2	0.004558321
BCL11A_AT	CLK2	0.001075802
BCL2_AT	CLK2	0.013650737
BCL2L11_AP	CLK2	0.002218205
BCL2L14_AA	CLK2	4.01528E-10
BCLAF1_ES	CLK2	8.04306E-17
BCLAF1_ES	CLK2	1.28645E-12
BCLAF1_AA	CLK2	0.000490998
BCMO1_AT	CLK2	1.32285E-06
BCS1L_AD	CLK2	1.19655E-08
BCS1L_RI	CLK2	1.8713E-15
BCS1L_AD	CLK2	0
BCS1L_ES	CLK2	3.6538E-17
BCS1L_AD	CLK2	5.76998E-14
BDKRB2_AA	CLK2	1.03555E-06
BDKRB2_AA	CLK2	7.05712E-07
BECN1_AD	CLK2	5.00557E-06
BET1_AT	CLK2	2.65401E-07
BET1L_AP	CLK2	0.00052885
BET1L_AT	CLK2	5.18041E-17
BET1L_RI	CLK2	7.34259E-12
BFAR_ES	CLK2	0.006507953
BIN1_ES	CLK2	7.03862E-05
BIN1_ES	CLK2	0.004093423
BIN1_ES	CLK2	5.05445E-06
BIN3_AT	CLK2	0
BIRC5_ES	CLK2	4.03885E-06
BLCAP_AP	CLK2	0
BLOC1S1_AP	CLK2	0.000177864
BLOC1S6_AT	CLK2	0
BLOC1S6_ES	CLK2	7.19412E-07

BLZF1_AT	CLK2	0.000412255
BMP1_AA	CLK2	0.000263811
BMP1_AA	CLK2	0
BMP2K_AT	CLK2	0.003691559
BMP4_AD	CLK2	0.000328219
BMP8B_AT	CLK2	1.14825E-07
BNC2_AT	CLK2	1.83011E-13
BNIP2_AP	CLK2	0.000123
BOD1_ES	CLK2	0.007143351
BOP1_RI	CLK2	1.69636E-17
BRD1_ES	CLK2	0.012746359
BRD4_AT	CLK2	0.008294853
BRD8_ES	CLK2	2.78516E-06
BRD9_AP	CLK2	1.12051E-08
BRD9_ES	CLK2	8.98941E-07
BRD9_ES	CLK2	0.000526219
BRD9_ES	CLK2	1.43826E-09
BRD9_ES	CLK2	4.54304E-20
BRD9_AA	CLK2	2.65788E-16
BRF1_AP	CLK2	0
BRF1_AA	CLK2	3.45472E-10
BRI3_AT	CLK2	0.016393779
BROX_AP	CLK2	0.0069258
BRPF1_ES	CLK2	0.000944941
BRWD1_AT	CLK2	5.88471E-07
BSDC1_RI	CLK2	0
BTBD6_RI	CLK2	0.002691369
BTBD7_AT	CLK2	3.37247E-07
BTF3_AD	CLK2	1.72948E-05
BTN2A1_AA	CLK2	6.88911E-18
BTN2A1_ES	CLK2	0.002038068
BTN2A2_ES	CLK2	0.000507496
BTN3A1_AA	CLK2	2.15484E-09
BTNL9_AT	CLK2	0.000312861
BUB3_AA	CLK2	0.014544813
BUD31_AP	CLK2	5.57059E-15
C10orf118_AT	CLK2	0.003765719
C11orf24_AD	CLK2	1.49797E-09
C11orf30_AT	CLK2	0
C11orf49_AT	CLK2	4.57042E-05
C11orf49_RI	CLK2	1.41694E-08
C11orf49_RI	CLK2	0
C11orf54_ES	CLK2	2.35379E-16
C11orf57_AD	CLK2	1.02173E-08
C11orf68_AP	CLK2	3.31295E-09
C11orf80_ES	CLK2	2.19919E-06
C12orf23_AP	CLK2	0.011557769
C12orf36_AT	CLK2	7.60555E-06
C12orf57_AD	CLK2	3.63107E-07
C12orf65_AP	CLK2	7.74327E-06
C12orf66_AT	CLK2	0.000602916
C12orf73_AP	CLK2	0.001844774
C12orf73_RI	CLK2	0.004483934

C12orf76_AT	CLK2	5.84255E-05
C14orf159_AP	CLK2	1.06695E-05
C14orf79_AP	CLK2	4.51921E-07
C14orf79_RI	CLK2	0.00037046
C14orf79_AD	CLK2	8.03104E-06
C14orf80_AP	CLK2	0.000509257
C14orf80_ES	CLK2	0.000974751
C15orf27_AT	CLK2	8.92468E-05
C15orf38_AP	CLK2	0.000126098
C15orf57_AT	CLK2	1.08912E-11
C16orf13_ES	CLK2	0.001705241
C16orf58_RI	CLK2	0
C16orf59_RI	CLK2	3.14249E-06
C17orf58_AD	CLK2	0.000239633
C17orf62_AP	CLK2	5.77536E-13
C17orf62_AP	CLK2	1.12444E-13
C17orf62_AD	CLK2	1.66894E-07
C17orf70_AP	CLK2	1.92844E-14
C17orf97_AT	CLK2	5.45666E-05
C18orf32_AD	CLK2	0.014211716
C19orf25_AA	CLK2	0
C19orf25_AD	CLK2	4.49044E-17
C19orf43_AA	CLK2	0
C19orf44_AT	CLK2	4.18285E-07
C19orf48_AP	CLK2	0
C19orf48_RI	CLK2	1.74676E-14
C19orf55_AT	CLK2	5.9796E-05
C19orf66_ES	CLK2	7.02865E-17
C19orf66_ES	CLK2	0.00403178
C19orf70_AT	CLK2	9.82849E-08
C19orf82_AT	CLK2	2.72027E-10
C1orf106_AP	CLK2	1.81433E-13
C1orf131_RI	CLK2	0
C1orf159_AD	CLK2	6.71564E-16
C1orf198_AP	CLK2	1.98701E-05
C1orf213_RI	CLK2	4.6122E-07
C1orf213_RI	CLK2	1.46931E-07
C1orf54_AP	CLK2	7.03088E-09
C1orf63_AA	CLK2	1.45258E-06
C1orf85_AT	CLK2	0.000207707
C1QTNF1_AP	CLK2	0.000767782
C1RL_AT	CLK2	6.43399E-18
C1RL_AA	CLK2	4.49417E-11
C1S_AP	CLK2	1.76343E-11
C20orf96_AT	CLK2	0
C20orf96_ES	CLK2	0.006316011
C21orf58_AP	CLK2	1.6432E-15
C21orf58_AP	CLK2	1.28763E-06
C21orf58_RI	CLK2	0.000364231
C22orf39_AT	CLK2	5.58522E-05
C2ORF15_AP	CLK2	1.15259E-05
C2ORF15_AT	CLK2	3.06702E-05
C2orf68_AT	CLK2	0

C2orf68_RI	CLK2	0.00058497
C2orf76_AP	CLK2	0
C3orf17_AT	CLK2	5.91889E-06
C3orf52_AT	CLK2	1.7786E-08
C4BPB_AT	CLK2	0.001299998
C4orf19_AT	CLK2	5.42494E-12
C4orf29_AT	CLK2	1.2402E-05
C4orf33_AP	CLK2	1.08762E-08
C4orf33_AT	CLK2	2.3324E-05
C4orf36_AT	CLK2	0.0019969
C5orf28_AP	CLK2	1.77332E-09
C5orf30_AP	CLK2	1.82508E-05
C5orf30_AP	CLK2	0.00677936
C5orf45_RI	CLK2	0
C5orf56_AT	CLK2	2.89361E-11
C5orf56_AT	CLK2	6.34302E-09
C5orf63_AT	CLK2	0.000225174
C5orf63_ES	CLK2	4.44494E-06
C6orf1_AD	CLK2	0.000384227
C6orf132_AT	CLK2	0
C6orf203_ES	CLK2	4.49227E-05
C6orf203_AD	CLK2	1.87124E-06
C7orf43_AP	CLK2	0.000115212
C7orf43_AA	CLK2	0
C8orf59_AT	CLK2	1.35957E-07
C8orf59_AD	CLK2	0.000647941
C8orf59_ES	CLK2	0.000260381
C9orf156_AP	CLK2	8.16393E-14
C9orf72_AT	CLK2	7.82126E-05
C9orf85_AT	CLK2	0.003187684
C9orf89_RI	CLK2	0
C9orf89_RI	CLK2	0
C9orf89_RI	CLK2	0
CACNB3_AP	CLK2	6.62573E-09
CALCOCO1_RI	CLK2	0
CALCOCO2_ES	CLK2	1.43529E-08
CALHM2_AP	CLK2	0.000467544
CALHM2_AT	CLK2	0.001564674
CALHM2_AA	CLK2	0.009167267
CALML4_ES	CLK2	1.39946E-06
CAMK2D_AT	CLK2	3.77938E-06
CAMKK2_AT	CLK2	7.50574E-07
CAMKMT_AT	CLK2	0.006124351
CAMLG_ES	CLK2	2.81237E-06
CAMLG_ES	CLK2	1.26987E-15
CAMTA1_AT	CLK2	5.72446E-08
CAMTA1_ES	CLK2	3.87937E-05
CANT1_ES	CLK2	0.002866058
CAPN10_AT	CLK2	2.92449E-11
CAPN10_ES	CLK2	0
CAPN13_AT	CLK2	5.29857E-14
CAPN15_ES	CLK2	2.74737E-05
CAPN2_AP	CLK2	5.32716E-05

CAPRIN1_AP	CLK2	0.005083242
CARD14_AT	CLK2	7.63748E-08
CARD6_AT	CLK2	0.019767121
CARD8_AT	CLK2	0.00011056
CARD8_ES	CLK2	1.50813E-05
CARKD_AA	CLK2	3.48839E-17
CARKD_RI	CLK2	0
CARKD_RI	CLK2	2.85322E-17
CARM1_RI	CLK2	0
CARS_ES	CLK2	0.000579345
CASC4_ES	CLK2	9.88009E-08
CASP7_AP	CLK2	6.43578E-07
CBFB_AD	CLK2	0.000165261
CBR4_ES	CLK2	0
CBWD1_AT	CLK2	0.002555049
CBWD2_ES	CLK2	4.78318E-13
CBWD3_AT	CLK2	0.004130961
CBWD5_AT	CLK2	9.14503E-05
CBWD6_AT	CLK2	0.015644095
CBY1_ES	CLK2	1.10872E-08
CC2D1B_AT	CLK2	0.007697613
CC2D1B_RI	CLK2	3.16501E-12
CCAR1_AD	CLK2	0.007076595
CCAR2_AP	CLK2	0.000648415
CCAR2_RI	CLK2	0
CCDC107_RI	CLK2	0.000273082
CCDC107_RI	CLK2	1.93781E-17
CCDC107_RI	CLK2	4.83472E-14
CCDC115_ES	CLK2	2.05906E-17
CCDC122_AT	CLK2	5.78425E-05
CCDC124_AP	CLK2	3.86162E-08
CCDC130_AP	CLK2	0.014298096
CCDC132_AT	CLK2	1.07348E-09
CCDC14_AP	CLK2	7.13808E-05
CCDC14_ES	CLK2	0.001457333
CCDC14_RI	CLK2	2.09109E-16
CCDC14_RI	CLK2	0
CCDC144A_AT	CLK2	8.5438E-07
CCDC157_AT	CLK2	7.56757E-05
CCDC24_RI	CLK2	0.000256391
CCDC24_ES	CLK2	0.001719476
CCDC28B_AT	CLK2	4.276E-09
CCDC28B_RI	CLK2	0
CCDC34_AT	CLK2	0.005210651
CCDC36_AT	CLK2	0.002728216
CCDC41_AT	CLK2	2.74154E-06
CCDC43_ES	CLK2	0.000932982
CCDC51_ES	CLK2	0.00441319
CCDC57_AT	CLK2	0
CCDC64B_AP	CLK2	1.65998E-11
CCDC64B_AP	CLK2	0
CCDC7_AT	CLK2	8.16069E-13
CCDC74A_RI	CLK2	0

CCDC86_AP	CLK2	0.046190912
CCDC88C_AT	CLK2	5.91377E-08
CCDC90B_AT	CLK2	0.002722685
CCDC90B_AD	CLK2	8.81249E-06
CCDC90B_AD	CLK2	5.76333E-08
CCDC92_AP	CLK2	0
CCDC92_RI	CLK2	0
CCL14_AP	CLK2	3.13413E-17
CCL24_AP	CLK2	0.004180366
CCL28_AP	CLK2	5.35512E-07
CCL28_AT	CLK2	0
CCNDBP1_AA	CLK2	8.33183E-13
CCNG2_AT	CLK2	1.38086E-06
CCNJL_AT	CLK2	1.57267E-06
CCNL1_AD	CLK2	2.56213E-07
CCNL1_ES	CLK2	1.12578E-06
CCNL2_AT	CLK2	0.004014559
CCNL2_ES	CLK2	3.89808E-09
CCNT1_ES	CLK2	0.000581199
CCNT2_AD	CLK2	3.18201E-08
CCPG1_RI	CLK2	1.88677E-08
CCS_AA	CLK2	0
CD151_AD	CLK2	0.006726134
CD276_AP	CLK2	4.25967E-06
CD276_AT	CLK2	9.76795E-07
CD33_AT	CLK2	0.00147132
CD34_AA	CLK2	5.27741E-05
CD37_AT	CLK2	0
CD44_ES	CLK2	0.000921466
CD44_ES	CLK2	0.000274257
CD44_ES	CLK2	3.08441E-06
CD44_ES	CLK2	0.000122296
CD44_ES	CLK2	1.84071E-05
CD44_ES	CLK2	0.000272645
CD44_ES	CLK2	4.24585E-07
CD44_ES	CLK2	0.00202531
CD44_ES	CLK2	0.00713681
CD55_AT	CLK2	0.005936799
CD58_AT	CLK2	0
CD58_AT	CLK2	0.006624167
CD58_AA	CLK2	0.005607076
CD79B_ES	CLK2	0.000178432
CD99L2_ES	CLK2	0.000130471
CDADC1_ES	CLK2	1.81813E-07
CDC14B_AT	CLK2	6.55909E-14
CDC27_AA	CLK2	0.001549244
CDC37_RI	CLK2	0
CDC37L1_AT	CLK2	0.04565773
CDCA3_AT	CLK2	0
CDCA7_ES	CLK2	1.6404E-18
CDH13_AT	CLK2	1.97199E-05
CDHR5_ES	CLK2	1.44848E-05
CDIP1_AP	CLK2	5.7694E-14

CDIPT_AP	CLK2	4.28596E-14
CDK1_ES	CLK2	0.002268672
CDK10_RI	CLK2	0
CDK10_ES	CLK2	5.64917E-12
CDK10_ES	CLK2	1.94757E-06
CDK10_ES	CLK2	0.002215315
CDK10_ES	CLK2	1.75497E-11
CDK16_AP	CLK2	0
CDK17_AT	CLK2	6.34181E-05
CDK18_AP	CLK2	2.77687E-15
CDK18_AD	CLK2	0.000268173
CDK18_RI	CLK2	0
CDK2_RI	CLK2	0
CDK2AP1_AP	CLK2	0.000167497
CDK5RAP3_RI	CLK2	0
CDK7_AP	CLK2	5.3466E-05
CDKL1_AT	CLK2	2.8E-14
CDKN1C_RI	CLK2	0.00053981
CDKN2A_AP	CLK2	0.00450057
CDKN2AIPNL_AT	CLK2	2.95748E-11
CDKN3_ES	CLK2	0.000805445
CEACAM1_ES	CLK2	8.88263E-07
CEACAM1_ES	CLK2	1.98983E-16
CEACAM1_ES	CLK2	1.26725E-08
CENPK_AT	CLK2	2.23965E-05
CENPM_AP	CLK2	6.28172E-14
CENPP_AP	CLK2	0
CENPT_AT	CLK2	0
CENPT_AA	CLK2	1.14041E-09
CENPV_AA	CLK2	2.6331E-05
CENPW_AD	CLK2	0.000143492
CEP57_AT	CLK2	9.85198E-05
CEP57L1_AT	CLK2	0.014126934
CEP63_AP	CLK2	4.21659E-05
CEP63_ES	CLK2	0.00466218
CEP63_ES	CLK2	0.000852307
CEP68_AT	CLK2	3.22739E-06
CEP68_AD	CLK2	1.06166E-17
CEP70_AT	CLK2	2.16709E-10
CEP76_AT	CLK2	1.17949E-08
CEP95_AT	CLK2	0.000303021
CERS2_AT	CLK2	0
CERS5_ES	CLK2	8.33043E-05
CES2_AD	CLK2	2.48777E-15
CES3_AP	CLK2	0
CETN3_AT	CLK2	9.21573E-09
CFH_AT	CLK2	2.70803E-05
CFL1_AP	CLK2	0.00348918
CFLAR_AP	CLK2	0.007109593
CFLAR_AT	CLK2	2.84944E-10
CFLAR_AT	CLK2	0
CFLAR_ES	CLK2	0.00314956
CGGBP1_RI	CLK2	1.03636E-13

CGGBP1_AA	CLK2	4.72714E-12
CHCHD10_AA	CLK2	0.002265907
CHCHD3_AT	CLK2	0.00237708
CHCHD7_AP	CLK2	0.014050117
CHD2_AT	CLK2	1.00569E-13
CHEK1_AP	CLK2	2.92881E-05
CHEK2_ME	CLK2	0.000317171
CHID1_AP	CLK2	0.011087493
CHM_AT	CLK2	6.59907E-17
CHMP1A_ES	CLK2	0.000839358
CHMP2A_AP	CLK2	0.004376535
CHN2_AP	CLK2	0.000176781
CHN2_AP	CLK2	0.00301209
CHORDC1_AP	CLK2	6.49004E-10
CHORDC1_AT	CLK2	0.025461332
CHORDC1_RI	CLK2	3.10721E-16
CHRAC1_ES	CLK2	0.000205188
CHST5_RI	CLK2	1.03014E-09
CHTF18_AD	CLK2	9.47531E-09
CHTF8_RI	CLK2	6.30137E-06
CHTOP_AP	CLK2	1.54638E-11
CHTOP_AT	CLK2	4.74725E-13
CHTOP_AD	CLK2	5.3716E-18
CHTOP_ES	CLK2	1.08578E-07
CIDEB_AP	CLK2	0.000151364
CIRBP_RI	CLK2	0.000369397
CIRBP_RI	CLK2	6.19365E-06
CIRBP_RI	CLK2	0
CIRBP_RI	CLK2	4.41693E-10
CIRBP_ES	CLK2	5.71082E-07
CIRBP_ES	CLK2	1.77614E-06
CIZ1_RI	CLK2	0.011543713
CIZ1_AA	CLK2	0.005576643
CKLF_AT	CLK2	1.11409E-06
CKLF_ES	CLK2	3.33193E-09
CKLF_AA	CLK2	1.09376E-12
CKLF-CMTM1_AT	CLK2	1.607E-08
CKMT1A_AA	CLK2	0
CKMT1B_AT	CLK2	0
CKMT1B_AA	CLK2	0
CKMT1B_AA	CLK2	4.88546E-16
CLASRP_AD	CLK2	0.000291391
CLCN3_ES	CLK2	0.002352452
CLCN6_AT	CLK2	0.002721209
CLDN11_AT	CLK2	9.51054E-06
CLDN7_AP	CLK2	0
CLDND1_AP	CLK2	5.40204E-16
CLDND1_AT	CLK2	4.89385E-05
CLEC16A_AT	CLK2	0.007619752
CLEC5A_AT	CLK2	8.95896E-05
CLINT1_AA	CLK2	6.7867E-07
CLK1_AP	CLK2	3.01121E-07
CLK2_AP	CLK2	0.000625627

CLK4_ES	CLK2	0.005213387
CLN3_AP	CLK2	0.00279485
CLNS1A_AT	CLK2	0.001590331
CLPTM1_AP	CLK2	2.20106E-07
CLSTN3_AP	CLK2	0
CLTB_ES	CLK2	3.77199E-06
CLTC_ES	CLK2	0.0138463
CMC4_AP	CLK2	1.79171E-10
CNDP2_AA	CLK2	0.000159015
CNIH4_AA	CLK2	0.001613856
CNKSR1_RI	CLK2	1.32094E-12
CNKSR1_AD	CLK2	1.28096E-05
CNN1_AP	CLK2	0
CNNM2_AT	CLK2	1.62667E-05
CNOT10_AT	CLK2	2.52218E-12
CNOT2_AT	CLK2	1.97624E-06
CNOT3_AP	CLK2	0
CNOT4_AT	CLK2	4.2899E-12
CNTROB_AD	CLK2	0
COA1_AT	CLK2	1.2296E-11
COA3_AT	CLK2	3.65705E-09
COA4_ES	CLK2	0.006723003
COA5_AT	CLK2	3.8653E-07
COA6_AP	CLK2	0.000845359
COASY_RI	CLK2	0.002141757
COASY_AA	CLK2	8.56345E-05
COASY_AD	CLK2	0.001831711
COG4_ES	CLK2	0
COG4_ES	CLK2	3.42189E-18
COL12A1_ES	CLK2	0
COL14A1_AT	CLK2	0.011980292
COL1A1_ES	CLK2	0.004072157
COL1A1_ES	CLK2	0.001416921
COL1A1_ES	CLK2	0.001647768
COL1A1_ES	CLK2	0.004653118
COL3A1_ES	CLK2	0.005027165
COL6A2_AT	CLK2	0.002238672
COL6A2_AA	CLK2	8.31089E-05
COL6A3_ES	CLK2	8.18172E-05
COL6A3_ES	CLK2	5.67689E-07
COMMD4_AT	CLK2	1.90183E-05
COMMD7_RI	CLK2	0
COMT_AP	CLK2	0.000112338
COMT_AA	CLK2	0.002121416
COPB1_AD	CLK2	1.47311E-08
COPS7A_AD	CLK2	0.001838146
COQ3_ES	CLK2	7.09257E-05
COQ4_AT	CLK2	1.23525E-05
COQ6_RI	CLK2	0.005847843
CORIN_AT	CLK2	6.55327E-05
CORO7_AD	CLK2	3.23456E-06
COX10_AT	CLK2	1.84293E-05
COX19_AT	CLK2	0.000136011

COX20_ES	CLK2	8.30039E-05
COX20_ES	CLK2	6.31739E-08
COX4I1_AA	CLK2	0
COX4I1_ES	CLK2	0
COX4I1_RI	CLK2	1.34082E-17
COX6C_AT	CLK2	3.7806E-06
COX7C_RI	CLK2	3.73816E-14
CPNE1_AD	CLK2	7.61658E-06
CPNE1_AA	CLK2	3.14662E-18
CPSF3L_AA	CLK2	0
CPSF3L_AA	CLK2	0
CPSF7_AD	CLK2	1.02598E-05
CRB3_AP	CLK2	7.05206E-11
CREB3L2_AP	CLK2	0.001920167
CREB3L2_AT	CLK2	0.004665033
CREB3L4_RI	CLK2	2.06515E-18
CREBRF_AT	CLK2	4.73594E-05
CREBZF_RI	CLK2	3.36104E-12
CRELD1_ES	CLK2	2.39035E-18
CRELD1_RI	CLK2	7.71691E-09
CRELD1_RI	CLK2	4.20325E-09
CRIP1_AT	CLK2	1.95373E-06
CRK_AD	CLK2	2.74568E-06
CRYZL1_AT	CLK2	0
CRYZL1_AA	CLK2	1.67338E-10
CSF1_AT	CLK2	7.6426E-07
CSNK1E_AA	CLK2	4.33454E-13
CSTF3_RI	CLK2	0
CTAGE5_AP	CLK2	0.00126552
CTAGE5_AP	CLK2	1.15901E-10
CTBP1_AP	CLK2	0
CTBP2_AP	CLK2	0.000335509
CTBP2_AP	CLK2	2.70353E-06
CTBS_AD	CLK2	5.93468E-07
CTNNBIP1_AP	CLK2	0.000289758
CTNND1_RI	CLK2	0.004700407
CTNND1_ES	CLK2	0.00015686
CTNS_AT	CLK2	7.74383E-06
CTPS2_AP	CLK2	0.004373358
CTSB_ES	CLK2	0.000339781
CTTN_ME	CLK2	1.09209E-10
CTTN_ES	CLK2	0
CTTN_ES	CLK2	0
CUL4A_AP	CLK2	0.000480267
CUL7_AD	CLK2	3.36434E-07
CUX1_AT	CLK2	3.16358E-12
CUZD1_AP	CLK2	4.2058E-08
CWC25_AD	CLK2	0
CX3CL1_AP	CLK2	0.012052712
CXCL12_AT	CLK2	4.23089E-10
CXCL12_AT	CLK2	0.000938144
CXCL12_ES	CLK2	1.41706E-16
CXorf36_AT	CLK2	0.000137065

CXorf38_RI	CLK2	7.14921E-17
CXorf38_AA	CLK2	0
CXorf40A_AP	CLK2	3.37418E-16
CXorf40A_RI	CLK2	0
CYB561A3_RI	CLK2	0
CYB561A3_RI	CLK2	0
CYB561A3_RI	CLK2	0
CYB561D2_RI	CLK2	2.42714E-05
CYB561D2_RI	CLK2	1.2947E-07
CYB5R2_ES	CLK2	0.003310155
CYGB_AP	CLK2	6.50345E-06
CYHR1_AT	CLK2	0.006832084
CYHR1_RI	CLK2	2.84981E-11
CYHR1_RI	CLK2	1.13883E-07
CYLD_AT	CLK2	0.003258181
CYP20A1_ES	CLK2	0.000170413
CYP3A5_AT	CLK2	0.009214105
CYP3A5_RI	CLK2	0
CYP3A5_ES	CLK2	4.14899E-07
CYP3A5_AA	CLK2	7.03903E-11
CYP3A5_ES	CLK2	0.005639642
CYP4F12_RI	CLK2	0
CYP4F12_RI	CLK2	0
CYP4F12_ES	CLK2	0
D2HGDH_ES	CLK2	0
D2HGDH_ES	CLK2	2.43054E-06
D2HGDH_ES	CLK2	0
D2HGDH_ES	CLK2	8.22155E-08
DAB2_ES	CLK2	1.07749E-06
DACT3_AT	CLK2	8.45037E-07
DAGLB_AP	CLK2	0.007700377
DALRD3_AA	CLK2	0.004099588
DAPK2_ES	CLK2	3.52203E-13
DAPK2_ES	CLK2	0.000159856
DARS2_ES	CLK2	0.002847212
DAZAP1_AT	CLK2	9.68207E-06
DAZAP1_ES	CLK2	4.65553E-16
DBF4_AD	CLK2	0.001234408
DBNDD2_AP	CLK2	0
DBNDD2_AP	CLK2	0
DBNDD2_AD	CLK2	7.62396E-07
DBNDD2_AD	CLK2	1.5212E-05
DCAF11_AP	CLK2	0.001065188
DCAF13_AT	CLK2	2.46705E-12
DCAF4_AT	CLK2	4.74499E-07
DCAF6_ES	CLK2	7.87355E-08
DCAF6_ES	CLK2	3.06065E-07
DCAF8_AP	CLK2	7.98957E-08
DCAF8_ES	CLK2	0
DCAF8_AD	CLK2	2.82865E-06
DCAF8_ES	CLK2	0.000248901
DCAF8_AD	CLK2	0.000177393
DCP1A_ES	CLK2	0.001782771

DCST1_AT	CLK2	1.75837E-15
DCTD_ES	CLK2	2.07659E-08
DCTD_ES	CLK2	8.66603E-08
DCTD_ES	CLK2	0.000197729
DCTN1_AP	CLK2	0.000480405
DCTN2_ES	CLK2	0.002667715
DCTN5_ES	CLK2	2.81299E-07
DCTPP1_AP	CLK2	0.001003216
DCUN1D2_ES	CLK2	1.28373E-08
DCUN1D4_AD	CLK2	1.02973E-17
DDB1_AP	CLK2	3.11593E-12
DDB2_ES	CLK2	0.000274584
DDC_AP	CLK2	0
DDIT3_RI	CLK2	0.000794821
DDIT3_RI	CLK2	7.97041E-06
DDRGK1_AT	CLK2	0
DDX10_AT	CLK2	0.000688526
DDX11_AT	CLK2	6.2844E-13
DDX11_RI	CLK2	0
DDX11_AA	CLK2	6.23017E-17
DDX11_AA	CLK2	0
DDX11_ES	CLK2	0.001485878
DDX17_AP	CLK2	2.29946E-05
DDX19B_AT	CLK2	3.53083E-07
DDX20_RI	CLK2	5.8751E-11
DDX20_RI	CLK2	8.06683E-14
DDX39A_AA	CLK2	0
DDX41_AA	CLK2	2.51668E-18
DDX55_AA	CLK2	0.000826887
DDX59_AT	CLK2	0.002428545
DECR1_ES	CLK2	1.79502E-09
DECR2_ES	CLK2	5.92524E-18
DECR2_ES	CLK2	9.05055E-09
DECR2_ES	CLK2	0
DEF8_AT	CLK2	6.3067E-05
DENND1B_AT	CLK2	0.00879545
DENND2A_AT	CLK2	2.70562E-15
DENND3_AP	CLK2	1.44158E-13
DENND3_AT	CLK2	1.55834E-08
DENND5B_AT	CLK2	1.72489E-10
DERL2_AD	CLK2	5.22759E-12
DERL3_RI	CLK2	8.90741E-11
DERL3_RI	CLK2	3.82921E-07
DFNB31_AP	CLK2	1.36238E-12
DGAT1_RI	CLK2	0
DGCR14_AA	CLK2	4.62509E-07
DGCR6_AT	CLK2	1.33816E-06
DGKD_ES	CLK2	1.11808E-10
DGUOK_ES	CLK2	0.000166286
DHDDS_AT	CLK2	0.002044343
DHDDS_AT	CLK2	5.91946E-08
DHDDS_AA	CLK2	1.06158E-15
DHPS_AD	CLK2	1.79864E-12

DHRS1_RI	CLK2	0
DHRS12_AT	CLK2	1.77609E-05
DHRS12_RI	CLK2	3.80251E-16
DHRS4L2_AP	CLK2	4.10971E-09
DHRSX_AP	CLK2	0.000198855
DHX30_AP	CLK2	8.77603E-08
DIAPH1_ES	CLK2	2.67063E-05
DIAPH2_AT	CLK2	0.000235738
DIDO1_AP	CLK2	0.013525219
DIDO1_AT	CLK2	0
DIMT1_AT	CLK2	7.24305E-10
DIS3L2_AT	CLK2	3.79067E-10
DIS3L2_AT	CLK2	9.14731E-17
DIS3L2_RI	CLK2	2.81957E-15
DIS3L2_RI	CLK2	9.56428E-17
DIS3L2_ES	CLK2	8.6345E-15
DIXDC1_AT	CLK2	0.004917338
DLD_AT	CLK2	2.34839E-10
DLG1_AT	CLK2	0.041946413
DLG1_ES	CLK2	4.27077E-09
DLG3_AP	CLK2	0.00209043
DLGAP5_ES	CLK2	9.99348E-06
DMAP1_RI	CLK2	0.009187689
DMKN_AP	CLK2	1.30141E-08
DMKN_AT	CLK2	0.000267874
DMPK_AP	CLK2	0
DMPK_ES	CLK2	3.32526E-06
DMPK_ES	CLK2	1.09941E-06
DMPK_RI	CLK2	9.22514E-07
DNAH2_AT	CLK2	0.001460553
DNAJA4_AP	CLK2	3.08035E-12
DNAJA4_AP	CLK2	4.51748E-05
DNAJB2_RI	CLK2	1.63196E-08
DNAJC10_AT	CLK2	2.96443E-08
DNAJC11_ES	CLK2	9.04649E-05
DNAJC12_AT	CLK2	5.08191E-05
DNAJC14_AP	CLK2	0.002051237
DNAJC16_AT	CLK2	0.007172522
DNAJC17_AA	CLK2	2.13135E-11
DNAJC19_AD	CLK2	0.000376851
DNAJC19_ES	CLK2	1.02844E-07
DNAJC2_ES	CLK2	2.6351E-12
DNAJC21_ES	CLK2	0.000582255
DNAJC24_AT	CLK2	0.008587575
DNAJC4_ES	CLK2	4.40828E-13
DNAJC7_AT	CLK2	3.27296E-05
DNASE1_AT	CLK2	0.00697948
DNASE1_AT	CLK2	2.03483E-05
DNASE1_RI	CLK2	0.001577828
DNASE1L1_AP	CLK2	1.29058E-11
DNASE1L1_AD	CLK2	9.76634E-12
DNM2_ES	CLK2	8.1735E-14
DNMT3A_AT	CLK2	1.578E-14

DNPEP_AD	CLK2	5.45551E-07
DOCK8_AT	CLK2	0.000124116
DOK1_AP	CLK2	3.76899E-07
DOK4_AP	CLK2	2.47719E-12
DOK4_RI	CLK2	3.91386E-09
DPAGT1_RI	CLK2	0
DPH2_AA	CLK2	4.06677E-18
DPH6_AT	CLK2	0.011839017
DPP8_AP	CLK2	2.60009E-11
DPY30_AD	CLK2	0.001050082
DPYSL3_AP	CLK2	0.006205475
DRG2_RI	CLK2	0
DRG2_RI	CLK2	8.29571E-05
DSN1_ES	CLK2	0.011100572
DTD2_RI	CLK2	1.9291E-05
DTWD1_AT	CLK2	7.46968E-06
DTX2_ES	CLK2	0.002388268
DUS4L_ES	CLK2	0.003343044
DUSP10_AP	CLK2	1.4748E-13
DUSP11_AT	CLK2	2.89745E-18
DUSP14_AP	CLK2	0.0004887
DUSP18_AT	CLK2	7.09431E-14
DUSP6_AP	CLK2	2.4062E-05
DUSP6_ES	CLK2	0.006081174
DUT_AP	CLK2	0.010209601
DYNC2LI1_AT	CLK2	6.83219E-06
DYNLL1_RI	CLK2	7.94535E-08
DYNLT1_AP	CLK2	2.44641E-11
DYNLT3_AT	CLK2	1.18115E-14
DYRK1B_AA	CLK2	7.16238E-06
E2F5_AP	CLK2	1.09912E-05
ECE1_AP	CLK2	2.19574E-06
ECE1_AP	CLK2	1.17751E-05
ECE2_AP	CLK2	0
ECE2_AT	CLK2	7.52454E-17
ECHDC1_AP	CLK2	2.25492E-07
ECHDC2_AT	CLK2	0
ECHDC2_AT	CLK2	0
ECHDC2_AT	CLK2	0
ECHDC2_AA	CLK2	5.0255E-12
ECHDC2_ES	CLK2	0
ECHDC2_ES	CLK2	1.75239E-09
ECHDC2_ES	CLK2	0.000262873
ECHDC2_ES	CLK2	7.89358E-06
ECHDC2_ES	CLK2	2.69703E-05
ECM2_AT	CLK2	0.001213001
ECT2_AT	CLK2	2.91924E-14
EDEM1_AT	CLK2	0.019282804
EDF1_RI	CLK2	0
EED_RI	CLK2	3.31502E-08
EEF1A1_ES	CLK2	1.30893E-11
EEF1A1_RI	CLK2	0
EEF1D_ME	CLK2	0.002078056

EEF1D_AP	CLK2	1.11525E-12
EEF1D_ES	CLK2	0.002147662
EEF1D_ES	CLK2	3.05462E-05
EEF1D_ES	CLK2	0.013934988
EEF1G_AP	CLK2	9.20881E-06
EFCAB2_AT	CLK2	0.014897576
EFNA3_AP	CLK2	3.74713E-06
EGFL7_AP	CLK2	0.001424948
EGLN3_AT	CLK2	0
EHBP1_ES	CLK2	2.20779E-05
EHBP1L1_AP	CLK2	0
EHF_AP	CLK2	0.00331308
EIF1AD_AD	CLK2	0.011339023
EIF2B1_AT	CLK2	0
EIF2B1_RI	CLK2	0
EIF2B3_AT	CLK2	0.009795867
EIF2B4_AA	CLK2	0.000997658
EIF2B4_RI	CLK2	0.005379859
EIF4A1_RI	CLK2	4.29014E-09
EIF4A1_AD	CLK2	1.23837E-05
EIF4A2_ES	CLK2	1.06161E-10
EIF4A2_ES	CLK2	2.46886E-07
EIF4B_AD	CLK2	2.52971E-07
EIF4E2_AT	CLK2	0.016017466
EIF4G1_AP	CLK2	0.005161925
EIF4H_ES	CLK2	7.20172E-07
EIF5_AP	CLK2	3.96585E-12
EIF6_AP	CLK2	1.48915E-06
EIF6_RI	CLK2	5.06361E-05
ELAC1_AT	CLK2	2.03428E-06
ELF2_AP	CLK2	0.005296608
ELF2_AA	CLK2	0.000289474
ELF3_RI	CLK2	0
ELF3_RI	CLK2	0
ELMO2_AP	CLK2	4.05237E-18
ELMOD3_RI	CLK2	0
ELMOD3_ES	CLK2	2.11978E-17
ELMOD3_RI	CLK2	9.9854E-07
ELMOD3_RI	CLK2	5.64331E-10
EMC4_AT	CLK2	7.41779E-11
EMC4_ES	CLK2	0.011730989
EMC9_AA	CLK2	2.97317E-13
EMC9_AA	CLK2	0
EML2_AP	CLK2	2.97086E-15
EML3_AP	CLK2	2.79332E-05
ENGASE_AT	CLK2	0
ENO2_AP	CLK2	0
ENO3_AT	CLK2	0.000125153
ENOSF1_AP	CLK2	9.97512E-11
ENOSF1_AT	CLK2	0
ENOSF1_ES	CLK2	0
ENSA_AT	CLK2	1.13024E-06
ENTHD2_AD	CLK2	0

ENTPD5_AT	CLK2	1.90921E-11
ENTPD6_ES	CLK2	4.54614E-05
ENY2_AA	CLK2	5.33463E-11
ENY2_AD	CLK2	0.000703251
EP400NL_AT	CLK2	0.005906194
EP400NL_AT	CLK2	0
EPB41L5_AT	CLK2	4.02031E-11
EPHA3_AT	CLK2	0.00058692
EPN3_AD	CLK2	0.008895207
EPS8L1_ES	CLK2	0
EPS8L2_RI	CLK2	0
EPS8L3_AA	CLK2	3.41531E-05
ERBB3_AP	CLK2	0
ERBB3_AP	CLK2	0.00043078
ERBB3_AT	CLK2	0.013003969
ERCC1_AT	CLK2	3.6641E-15
ERCC1_ES	CLK2	5.37357E-07
ERCC4_AT	CLK2	6.95759E-10
ERCC5_RI	CLK2	0
ERF_AP	CLK2	1.83127E-05
ERICH1_AT	CLK2	0.002334183
ERMP1_AT	CLK2	0.007038114
ERN2_RI	CLK2	0
ERN2_AA	CLK2	0
ERN2_ES	CLK2	0
ERO1LB_AT	CLK2	0.006868511
ERRFI1_RI	CLK2	0
ERRFI1_AA	CLK2	2.03208E-06
ESAM_AT	CLK2	7.49501E-05
ESCO2_AT	CLK2	7.63571E-06
ESRP1_AD	CLK2	7.46761E-14
ESRP2_AA	CLK2	1.65525E-14
ESRRA_AP	CLK2	0
ESYT2_AP	CLK2	0.00114593
ETFA_ES	CLK2	0.000131643
ETFB_AP	CLK2	1.83643E-15
ETS1_AP	CLK2	1.23763E-06
ETV4_AP	CLK2	0
ETV7_AT	CLK2	0.001299068
EVA1A_AP	CLK2	0.005008803
EVL_AT	CLK2	1.4559E-19
EVPL_AD	CLK2	2.19277E-13
EWSR1_AT	CLK2	0
EXD3_AT	CLK2	0.002723055
EXOC3_RI	CLK2	0
EXOC3_AD	CLK2	0
EXOC6B_AT	CLK2	0.00535718
EXOC7_ES	CLK2	0
EXOC7_ES	CLK2	0
EXOC7_ES	CLK2	0.000594193
EXOSC10_RI	CLK2	0
EXOSC10_RI	CLK2	0
EXOSC3_AT	CLK2	0

EXOSC3_ES	CLK2	6.79591E-07
EXOSC9_RI	CLK2	2.43149E-08
EXOSC9_AA	CLK2	0.004839147
EZH2_ES	CLK2	3.47016E-11
EZH2_ES	CLK2	3.77278E-05
EZR_AP	CLK2	1.40899E-11
FADS3_RI	CLK2	0
FAH_AP	CLK2	2.67344E-09
FAHD1_AT	CLK2	0.004403317
FAM101A_AP	CLK2	2.6511E-05
FAM102A_AP	CLK2	0
FAM102B_AT	CLK2	0.000633891
FAM104B_AT	CLK2	0.002328853
FAM104B_AD	CLK2	0.008018218
FAM109A_AP	CLK2	4.30785E-14
FAM111A_RI	CLK2	2.71889E-09
FAM111A_AA	CLK2	1.02951E-12
FAM111A_ES	CLK2	0.000359897
FAM115A_AD	CLK2	5.42942E-06
FAM115C_AT	CLK2	0.003670705
FAM118A_AP	CLK2	0.005126221
FAM118A_AT	CLK2	0.000117947
FAM120AOS_AP	CLK2	2.00116E-05
FAM120C_AT	CLK2	2.62512E-05
FAM122B_AA	CLK2	0.010551506
FAM129C_AA	CLK2	5.92302E-10
FAM131A_AP	CLK2	1.05652E-12
FAM131A_RI	CLK2	0
FAM134B_AP	CLK2	7.92562E-11
FAM135A_AA	CLK2	1.50562E-06
FAM136A_ES	CLK2	1.30564E-18
FAM156B_AP	CLK2	1.99901E-05
FAM156B_RI	CLK2	2.49641E-13
FAM156B_AD	CLK2	0.004139939
FAM160A2_AT	CLK2	5.48932E-18
FAM160A2_AD	CLK2	8.30208E-08
FAM173A_RI	CLK2	1.4642E-16
FAM173A_AA	CLK2	7.35439E-12
FAM175A_AT	CLK2	1.79407E-07
FAM178A_AT	CLK2	0.011333591
FAM192A_AP	CLK2	2.36054E-11
FAM192A_AP	CLK2	0.002716688
FAM192A_AD	CLK2	3.68032E-05
FAM193B_ES	CLK2	8.19387E-05
FAM193B_ES	CLK2	0.003650869
FAM195B_AA	CLK2	0.005384324
FAM195B_RI	CLK2	2.51985E-06
FAM204A_ES	CLK2	0.000809637
FAM211B_ES	CLK2	2.14413E-07
FAM213A_AP	CLK2	0
FAM21A_ES	CLK2	0.00401271
FAM21A_ES	CLK2	6.88587E-05
FAM222B_AP	CLK2	0.000160869

FAM3D_AP	CLK2	1.87339E-15
FAM60A_AP	CLK2	0.000298257
FAM63A_AA	CLK2	0.000434513
FAM63A_AA	CLK2	4.53432E-07
FAM64A_AT	CLK2	2.87443E-07
FAM69B_AP	CLK2	4.94072E-06
FAM72A_AP	CLK2	0.014463733
FAM72A_AT	CLK2	0.007710425
FAM73B_AD	CLK2	0
FAM73B_AA	CLK2	0
FAM84A_ES	CLK2	0.000249523
FAM86A_ES	CLK2	0.000373861
FAM86C1_AT	CLK2	0
FAM91A1_AT	CLK2	4.26178E-05
FAM92A1_AP	CLK2	3.07027E-16
FAM92A1_AT	CLK2	9.17629E-10
FAM98B_AT	CLK2	7.29664E-10
FAN1_AT	CLK2	3.87986E-14
FANCA_AT	CLK2	0.000325738
FANCM_AT	CLK2	0.000649529
FAR1_AP	CLK2	7.89576E-11
FARP2_AT	CLK2	0.000859792
FARS2_AP	CLK2	2.13846E-07
FASTK_RI	CLK2	0
FASTK_RI	CLK2	0
FASTK_RI	CLK2	0
FASTK_RI	CLK2	0
FASTK_RI	CLK2	0
FASTK_AD	CLK2	5.55521E-05
FASTK_ES	CLK2	0.000592542
FASTK_AD	CLK2	0
FASTK_AA	CLK2	5.26593E-14
FASTKD2_AD	CLK2	0.00306665
FAU_AA	CLK2	0.002641961
FAU_AD	CLK2	0.001456106
FBLIM1_AT	CLK2	0
FBLN5_AP	CLK2	8.24912E-08
FBXL12_AP	CLK2	1.75656E-12
FBXL12_AT	CLK2	8.06148E-13
FBXL12_ES	CLK2	1.11083E-06
FBXL12_ES	CLK2	0.000486169
FBXL12_RI	CLK2	0.00084778
FBXL8_RI	CLK2	4.18628E-17
FBXO10_AT	CLK2	1.40456E-05
FBXO3_AP	CLK2	0
FBXO3_RI	CLK2	1.2619E-06
FBXO38_AD	CLK2	3.95971E-06
FBXO4_AT	CLK2	2.35764E-06
FBXO44_ES	CLK2	4.01171E-08
FBXW7_AP	CLK2	0.007124985
FCHO2_AT	CLK2	2.7612E-06
FCHSD1_AT	CLK2	1.65975E-09
FCHSD1_ES	CLK2	0.000127724

FCHSD2_AT	CLK2	7.00156E-06
FCRL5_AT	CLK2	0.002500646
FDFT1_AP	CLK2	3.69397E-05
FDPS_ES	CLK2	0.004659573
FDPS_ES	CLK2	1.62317E-07
FDPS_AD	CLK2	1.54573E-10
FDPS_ES	CLK2	2.53278E-10
FDPS_ES	CLK2	2.80114E-05
FDPS_ES	CLK2	5.91902E-14
FDPS_AA	CLK2	0.000625278
FDPS_AD	CLK2	0
FEZ2_AP	CLK2	8.69797E-05
FGD3_RI	CLK2	1.42204E-06
FGFR2_AT	CLK2	2.19376E-06
FGFR3_ME	CLK2	0.000171818
FGFR4_AD	CLK2	0.006029976
FGFR4_RI	CLK2	7.27343E-16
FGFR4_RI	CLK2	2.01842E-05
FGFR4_AD	CLK2	6.55357E-05
FHIT_AT	CLK2	0
FHL2_AD	CLK2	1.99107E-05
FHL2_AD	CLK2	0.001061881
FIBP_AD	CLK2	0.001081256
FILIP1L_AT	CLK2	5.93865E-05
FIP1L1_AT	CLK2	0.004750372
FIS1_AP	CLK2	0
FKBP10_AD	CLK2	0
FKBP11_AP	CLK2	1.46677E-12
FKBP11_AT	CLK2	8.74007E-06
FKBP2_AP	CLK2	1.00629E-06
FKBP5_AP	CLK2	0
FLAD1_AT	CLK2	0.003753614
FLAD1_RI	CLK2	0
FLAD1_RI	CLK2	6.24982E-08
FLCN_AT	CLK2	8.05256E-08
FLJ27365_AT	CLK2	0.000191498
FLNA_ES	CLK2	8.351E-10
FLNB_AD	CLK2	0.000278948
FLT1_AT	CLK2	9.79852E-05
FMNL1_ES	CLK2	1.70974E-11
FMNL3_ES	CLK2	0.005275268
FMO5_AT	CLK2	1.67004E-11
FN1_AA	CLK2	0.001445611
FN1_ES	CLK2	0.000137241
FN1_ES	CLK2	1.16299E-06
FNDC3A_AP	CLK2	0.002531967
FNDC3B_AT	CLK2	2.91644E-07
FNTA_AP	CLK2	1.3355E-05
FOLR2_AA	CLK2	2.32684E-14
FOS_AP	CLK2	1.22263E-15
FOS_RI	CLK2	5.60669E-13
FOS_RI	CLK2	3.97179E-14
FOSB_AP	CLK2	3.44196E-09

FOSB_AT	CLK2	6.87656E-06
FOXJ2_AT	CLK2	1.93237E-16
FOXJ3_AP	CLK2	0.000143766
FOXJ3_AP	CLK2	5.50634E-16
FOXRED1_AD	CLK2	0
FRG1_ES	CLK2	0.000141747
FSD1L_AT	CLK2	0.001294543
FUBP1_AT	CLK2	1.58165E-09
FUK_AT	CLK2	0
FUOM_AA	CLK2	0.000415836
FUT3_ES	CLK2	1.00046E-05
FUT3_AA	CLK2	5.93598E-11
FUT3_AA	CLK2	6.65494E-18
FUT8_AP	CLK2	7.18182E-05
FXYD3_RI	CLK2	0
FXYD3_AA	CLK2	3.60276E-16
FXYD3_RI	CLK2	0.006557264
FYN_AP	CLK2	7.1253E-10
FYN_ES	CLK2	9.40716E-11
G6PD_AP	CLK2	2.75698E-08
GABPA_AP	CLK2	0.007411721
GABPB1_AD	CLK2	0.001374403
GABRA2_AT	CLK2	3.07153E-05
GADD45G_RI	CLK2	0.000297218
GALK2_AP	CLK2	2.21198E-05
GALNT10_AT	CLK2	0.002067609
GALNT7_AA	CLK2	1.10343E-13
GALT_AA	CLK2	5.2121E-10
GANC_AT	CLK2	3.20616E-05
GAR1_AP	CLK2	0.001061253
GART_AT	CLK2	8.75135E-06
GAS2L1_RI	CLK2	1.53133E-13
GAS6_AP	CLK2	0.004153087
GAS6_AP	CLK2	1.13169E-09
GATA6_AP	CLK2	0.003718128
GBA_AP	CLK2	2.54565E-11
GBA2_AP	CLK2	1.17043E-05
GBA2_RI	CLK2	2.09441E-10
GBA2_RI	CLK2	2.57444E-08
GBA2_RI	CLK2	2.39779E-11
GBA2_AA	CLK2	2.47324E-13
GBP3_AD	CLK2	7.68654E-09
GBP3_ES	CLK2	0.002431824
GCAT_AT	CLK2	0.000324128
GCDH_ME	CLK2	1.75957E-06
GCDH_AD	CLK2	0
GCFC2_AT	CLK2	0.014232045
GCHFR_AT	CLK2	0.003374052
GDA_RI	CLK2	4.14134E-06
GDA_RI	CLK2	5.71824E-09
GDAP2_AT	CLK2	5.98375E-06
GDPD1_AT	CLK2	2.98608E-05
GDPD5_AP	CLK2	0

GEMIN4_AP	CLK2	2.19672E-11
GEMIN7_ES	CLK2	0.003027224
GEMIN7_ES	CLK2	1.82445E-05
GEMIN7_AD	CLK2	4.4958E-06
GET4_AP	CLK2	0
GFER_AP	CLK2	0.004673759
GFOD1_AT	CLK2	0.00202386
GGA1_AP	CLK2	4.7035E-08
GGA1_AT	CLK2	0
GGA2_AA	CLK2	0
GGA3_AA	CLK2	3.23744E-11
GGA3_RI	CLK2	0
GGACT_ES	CLK2	9.22412E-07
GGNBP2_AT	CLK2	0.01126953
GGPS1_AP	CLK2	0
GGT1_AP	CLK2	0
GGT1_AA	CLK2	4.95282E-15
GHDC_AP	CLK2	0.011171675
GHDC_AA	CLK2	6.55399E-07
GID4_AT	CLK2	0.006864595
GINS3_ES	CLK2	1.10254E-05
GIT2_AA	CLK2	0.000899386
GJB1_AP	CLK2	8.83251E-07
GJB3_AP	CLK2	0.000626189
GK_ES	CLK2	1.59837E-12
GK5_ES	CLK2	2.44389E-08
GLI4_AT	CLK2	1.14789E-16
GLI4_RI	CLK2	0.000679508
GLIPR1L2_AT	CLK2	1.82511E-05
GLOD4_ES	CLK2	1.04234E-05
GLRX_AA	CLK2	0
GLS_AP	CLK2	5.72816E-12
GLUL_AD	CLK2	2.17801E-18
GLYR1_RI	CLK2	0.006092652
GMFG_RI	CLK2	5.72517E-14
GMNN_AD	CLK2	6.06641E-06
GMPPA_RI	CLK2	8.06255E-07
GMPPA_AD	CLK2	0.007707448
GMPPA_AD	CLK2	0.001061357
GMPR2_RI	CLK2	0
GMPR2_AD	CLK2	4.62889E-10
GNAI2_AP	CLK2	9.58999E-06
GNAO1_AT	CLK2	2.27379E-05
GNB2L1_ES	CLK2	0
GNB2L1_AD	CLK2	2.9525E-07
GNPDA1_AP	CLK2	1.87812E-08
GNPDA2_AT	CLK2	2.18658E-08
GNPDA2_ES	CLK2	0.00019399
GOLGA2_AT	CLK2	0.002045975
GOLGA3_AD	CLK2	1.86752E-05
GOLGA4_ES	CLK2	0.000176164
GOLM1_AP	CLK2	9.60155E-06
GORASP1_AP	CLK2	2.30208E-13

GORASP1_AA	CLK2	0
GOSR1_AD	CLK2	0.009992729
GPATCH2L_AT	CLK2	0.001366885
GPATCH8_AT	CLK2	1.89406E-06
GPD2_AP	CLK2	0.004853641
GPN1_AP	CLK2	0.000730194
GPNMB_AT	CLK2	0.000535556
GPR35_AP	CLK2	0
GPR35_AP	CLK2	0
GPR56_AP	CLK2	0.000657601
GPR56_AP	CLK2	0.001281185
GPR56_ES	CLK2	0.01492487
GPR89A_AA	CLK2	4.15126E-14
GPS1_RI	CLK2	2.0332E-14
GPS1_AA	CLK2	0.000110648
GPS1_AD	CLK2	3.31396E-14
GPS1_ES	CLK2	0.000211083
GRAMD1A_AT	CLK2	0.002965282
GRB7_AP	CLK2	6.06403E-08
GREB1_AT	CLK2	9.61021E-05
GRIPAP1_AT	CLK2	0
GRTP1_AT	CLK2	6.88641E-20
GSDMB_AP	CLK2	0
GSK3A_AP	CLK2	2.68174E-07
GSK3B_ES	CLK2	0.005869366
GSKIP_AD	CLK2	6.39412E-10
GSN_AP	CLK2	0.000354705
GSS_RI	CLK2	0
GSTCD_AT	CLK2	0.009659066
GSTK1_AT	CLK2	1.11483E-05
GSTK1_RI	CLK2	0
GSTO2_AT	CLK2	4.09214E-05
GTF2H1_AP	CLK2	2.09836E-11
GTF2H1_AP	CLK2	1.10276E-06
GTF2H2C_AT	CLK2	0.004358363
GTF2IRD2_AT	CLK2	2.1026E-06
GTF2IRD2B_AT	CLK2	3.74436E-10
GTF3C2_AP	CLK2	1.48354E-06
GTPBP3_RI	CLK2	1.80726E-19
GTPBP8_AT	CLK2	0.001368901
GUK1_AP	CLK2	0
GUK1_ES	CLK2	5.98423E-14
GULP1_AT	CLK2	6.57655E-08
H2AFV_ES	CLK2	1.59511E-08
H2AFY_ME	CLK2	2.84262E-05
H3F3A_AP	CLK2	1.50692E-10
HAGHL_RI	CLK2	0
HAGHL_RI	CLK2	7.45359E-15
HARS_AP	CLK2	1.15701E-05
HARS2_RI	CLK2	0
HARS2_AA	CLK2	1.66306E-16
HAS3_AT	CLK2	0.001634996
HAT1_AT	CLK2	1.65586E-08

HAUS4_ES	CLK2	3.64793E-06
HAUS4_AD	CLK2	1.25444E-09
HAUS4_AD	CLK2	1.40493E-11
HAUS5_RI	CLK2	0
HAUS6_AT	CLK2	5.92467E-17
HBS1L_AP	CLK2	0.000602759
HCCS_AD	CLK2	0.000608652
HCLS1_AA	CLK2	8.97073E-11
HDAC10_RI	CLK2	0
HDAC4_AT	CLK2	8.01654E-17
HDAC6_RI	CLK2	0
HDAC9_AT	CLK2	0
HDHD3_AP	CLK2	0
HDLBP_AP	CLK2	0.000135984
HELZ2_AP	CLK2	0
HEMK1_RI	CLK2	0
HENMT1_ES	CLK2	0.000109783
HERC4_AT	CLK2	8.3491E-16
HES4_RI	CLK2	0.001084643
HES6_RI	CLK2	9.9163E-07
HES6_RI	CLK2	0.000615799
HEXB_AP	CLK2	8.72781E-16
HGSNAT_AT	CLK2	0.01510514
HIGD1A_AP	CLK2	0.014288099
HINFP_AA	CLK2	9.82718E-16
HIST1H2BD_AT	CLK2	2.57809E-07
HKR1_AP	CLK2	3.81339E-14
HM13_AD	CLK2	0.007374271
HMBS_AD	CLK2	9.11351E-07
HMBS_RI	CLK2	0
HMBS_AD	CLK2	1.23354E-06
HMG20B_AA	CLK2	0
HMGA1_AP	CLK2	5.33863E-05
HMGA1_AD	CLK2	3.10467E-05
HMGB1_RI	CLK2	0.000293395
HMGB1_RI	CLK2	5.11741E-13
HMGN1_AP	CLK2	6.47273E-14
HMGN3_AD	CLK2	0.000826769
HMHA1_AP	CLK2	0.000991966
HNF1A_AT	CLK2	2.11873E-06
HNF4A_AP	CLK2	8.07142E-07
HNF4A_AD	CLK2	3.22927E-10
HNMT_AT	CLK2	6.02423E-10
HNRNPA1_ES	CLK2	1.19405E-14
HNRNPA1_ES	CLK2	1.71775E-08
HNRNPA1_RI	CLK2	1.39522E-15
HNRNPA1_ES	CLK2	8.96422E-09
HNRNPA2B1_RI	CLK2	2.66423E-08
HNRNPA2B1_AA	CLK2	0
HNRNPA2B1_ES	CLK2	2.59977E-05
HNRNPA3_AD	CLK2	0.004510488
HNRNPD_AT	CLK2	6.81133E-08
HNRNPD_ES	CLK2	0.008929456

HNRNPDL_ES	CLK2	1.85761E-09
HNRNPH1_AD	CLK2	0.000179953
HNRNPK_AP	CLK2	0.012680566
HNRNPK_AA	CLK2	0.014790397
HNRNPLL_AT	CLK2	0.001421212
HNRNPU_AD	CLK2	0.003334087
HNRNPUL1_AA	CLK2	0.007958411
HNRNPUL1_AA	CLK2	5.82811E-05
HOOK2_AP	CLK2	0.000363463
HOOK2_ES	CLK2	0.004869541
HOOK2_AD	CLK2	0
HOXA10_AP	CLK2	4.04047E-05
HOXB3_AT	CLK2	2.58921E-15
HOXB6_AA	CLK2	5.75784E-09
HOXB9_AD	CLK2	2.88141E-06
HP1BP3_AP	CLK2	6.88349E-11
HP1BP3_AT	CLK2	2.63738E-16
HPS4_RI	CLK2	3.01111E-10
HPS4_RI	CLK2	0
HPS4_AA	CLK2	3.8258E-07
HPS4_AD	CLK2	2.17727E-06
HPS4_AA	CLK2	0.000392904
HPS4_ES	CLK2	0.000771303
HRAS_ES	CLK2	6.94264E-09
HS2ST1_AT	CLK2	7.68208E-05
HSD17B7_AA	CLK2	2.72257E-05
HSD3B7_AA	CLK2	1.9242E-09
HSF4_RI	CLK2	1.05328E-06
HSH2D_RI	CLK2	1.91596E-13
HSH2D_RI	CLK2	0
HSP90AA1_AT	CLK2	2.38365E-05
HSPB11_AT	CLK2	5.0828E-08
HTRA3_AT	CLK2	0.001189356
HYI_AT	CLK2	6.68573E-11
HYKK_AT	CLK2	1.07056E-05
HYKK_ES	CLK2	0.001354709
IAH1_AP	CLK2	1.39439E-05
IAH1_AT	CLK2	4.22772E-06
IAH1_ES	CLK2	0.000250386
IARS_AD	CLK2	0.001265412
ICA1_AP	CLK2	0.004213233
ICAM3_RI	CLK2	6.81524E-08
IDE_ES	CLK2	7.12257E-05
IDH3G_RI	CLK2	9.11669E-15
IDS_AP	CLK2	0
IDS_AT	CLK2	0.00051567
IDUA_AP	CLK2	0
IDUA_RI	CLK2	0.003289854
IDUA_RI	CLK2	2.66869E-09
IFI27L1_AD	CLK2	7.22829E-08
IFI27L1_ES	CLK2	0.002839756
IFI35_AD	CLK2	4.9452E-05
IFI44_AT	CLK2	1.30064E-12

IFI6_AD	CLK2	0.003711832
IFNAR2_ES	CLK2	0.004323473
IFNLR1_ES	CLK2	3.08516E-06
IFRD2_RI	CLK2	0
IFT172_AT	CLK2	1.93309E-07
IFT172_ES	CLK2	3.21236E-11
IFT20_AT	CLK2	0
IFT81_AD	CLK2	7.37593E-05
IFT88_ES	CLK2	2.68208E-06
IGF2_AP	CLK2	2.11903E-05
IGFLR1_ES	CLK2	0.000119876
IGFLR1_ES	CLK2	0.000419334
IGSF9_AA	CLK2	0.00317876
IKBIP_AT	CLK2	9.57699E-10
IKBKB_AT	CLK2	0.007968291
IL11RA_AP	CLK2	0
IL11RA_AT	CLK2	2.32392E-16
IL13RA1_AP	CLK2	9.5436E-15
IL15_AP	CLK2	1.65747E-10
IL15_ES	CLK2	0.003664035
IL17RC_AD	CLK2	0
IL17RC_AA	CLK2	5.03443E-05
IL17RE_AA	CLK2	1.58332E-12
IL17RE_AA	CLK2	3.63526E-16
IL18_ES	CLK2	2.67868E-05
IL18BP_RI	CLK2	1.39089E-15
IL18BP_RI	CLK2	0.000189796
IL32_RI	CLK2	0.016208115
IL32_AD	CLK2	0.000548345
IL4R_ES	CLK2	3.12583E-12
ILF3_AT	CLK2	5.67673E-06
ILF3_RI	CLK2	0.000260206
IMMP2L_AP	CLK2	1.88703E-14
IMPA1_ES	CLK2	0
IMPDH1_AP	CLK2	0.000389116
INADL_AT	CLK2	3.94517E-07
INADL_AT	CLK2	2.24756E-05
INADL_ES	CLK2	0.000469352
ING1_AP	CLK2	0.000698562
ING2_AP	CLK2	1.94655E-07
ING4_ES	CLK2	0.000333775
INO80B_AT	CLK2	2.18234E-08
INO80C_AT	CLK2	6.84943E-06
INO80E_AT	CLK2	4.67103E-10
INO80E_ES	CLK2	5.20094E-13
INO80E_ES	CLK2	2.01342E-14
INO80E_AA	CLK2	9.15578E-10
INPP4A_AT	CLK2	0.000174037
INPP4A_AD	CLK2	0.002819148
INPP4B_AT	CLK2	1.38598E-05
INPP5B_AT	CLK2	2.37384E-09
INPP5K_AT	CLK2	0.005018489
INPPL1_AP	CLK2	4.22278E-18

INTS12_AP	CLK2	0.014147457
INTS3_RI	CLK2	0
INTS6_AT	CLK2	0.000528542
INTS8_AA	CLK2	0.000853823
IP6K2_RI	CLK2	1.26719E-08
IP6K2_RI	CLK2	0.000144593
IP6K2_RI	CLK2	2.95494E-09
IP6K2_RI	CLK2	0
IP6K2_RI	CLK2	0
IP6K2_AA	CLK2	2.08852E-09
IP6K2_AD	CLK2	4.39847E-12
IP6K2_AD	CLK2	0
IP6K2_AD	CLK2	2.37966E-09
IPO11_AT	CLK2	0.003338217
IPO13_AP	CLK2	0
IQCG_AT	CLK2	0.006030987
IQCK_AT	CLK2	1.59196E-06
IREB2_RI	CLK2	0.000149805
IRF3_AA	CLK2	1.39721E-05
IRF3_AD	CLK2	0
IRF3_RI	CLK2	1.29275E-05
IRF3_RI	CLK2	0
IRF7_AP	CLK2	0.002591995
IRF7_RI	CLK2	0
IRF7_RI	CLK2	0.014082498
IRF8_AP	CLK2	0.000271951
ISCA1_AT	CLK2	7.06354E-13
ISCU_RI	CLK2	2.27575E-07
ISCU_ES	CLK2	3.18569E-17
ISG20_AT	CLK2	7.26285E-08
ISG20L2_RI	CLK2	0.000153244
ISOC2_AA	CLK2	0.007539779
IST1_AP	CLK2	0.016273939
IST1_ES	CLK2	4.35762E-07
IST1_ES	CLK2	0
IST1_ES	CLK2	0.00269973
ITFG2_AD	CLK2	1.21781E-10
ITGA6_ES	CLK2	9.07955E-06
ITGB1BP1_AP	CLK2	0.000565325
ITGB3_AT	CLK2	0.008635174
ITGB3BP_ES	CLK2	0.015707313
ITGBL1_AT	CLK2	0.010187081
ITIH5_AT	CLK2	7.42812E-07
ITPKB_AT	CLK2	0.008813592
ITSN1_ES	CLK2	0.000361903
IVNS1ABP_ES	CLK2	4.44574E-05
IYD_AT	CLK2	1.98668E-13
JAM2_AT	CLK2	4.90517E-07
JKAMP_AD	CLK2	2.06129E-07
JMJD1C_AP	CLK2	0.000172296
JMJD7_RI	CLK2	0
JTB_RI	CLK2	0
KALRN_AP	CLK2	4.67599E-06

KANSL2_AD	CLK2	0.002201229
KANSL2_AD	CLK2	0.005331846
KANSL3_ES	CLK2	2.33856E-07
KAT5_RI	CLK2	0.003163674
KAZN_AT	CLK2	1.56414E-13
KBTBD3_AT	CLK2	0.000130229
KBTBD3_ES	CLK2	0.000884548
KCNC4_AA	CLK2	0.002054692
KCND1_AP	CLK2	2.5609E-07
KCNE3_AP	CLK2	7.31916E-13
KCNH2_AT	CLK2	2.0836E-05
KCNMA1_AT	CLK2	2.96183E-06
KCNMB3_AP	CLK2	5.43717E-10
KCTD1_AP	CLK2	7.41889E-05
KCTD10_AP	CLK2	0
KCTD20_ES	CLK2	0.001125104
KDM2B_AT	CLK2	1.97103E-05
KDM4B_ES	CLK2	0.002307539
KDM6B_RI	CLK2	2.2779E-06
KHK_ME	CLK2	3.04094E-07
KIAA0040_AP	CLK2	4.99884E-05
KIAA0101_ES	CLK2	0.000323661
KIAA0141_RI	CLK2	1.29067E-07
KIAA0226L_AP	CLK2	0.006197031
KIAA0319L_AP	CLK2	5.65333E-05
KIAA0907_RI	CLK2	0
KIAA0930_AP	CLK2	0.007787524
KIAA1257_AT	CLK2	0.000109026
KIAA1328_AT	CLK2	1.28788E-09
KIF12_ES	CLK2	5.30179E-10
KIF12_ES	CLK2	0
KIF13A_AT	CLK2	0.002767034
KIF13A_AT	CLK2	6.38626E-07
KIF13A_ES	CLK2	0.005732346
KIF16B_AT	CLK2	6.72132E-09
KIF20B_AT	CLK2	0.004581586
KIF9_AT	CLK2	0.009683593
KIF9_AD	CLK2	0.000612255
KIN_AT	CLK2	0.011921569
KLC1_RI	CLK2	1.06011E-12
KLC2_AP	CLK2	0
KLC2_RI	CLK2	0
KLF10_AP	CLK2	0.002743215
KLF3_AT	CLK2	1.20534E-08
KLHDC2_RI	CLK2	0
KLHDC4_AT	CLK2	1.93686E-08
KLHDC4_AT	CLK2	4.59316E-08
KLHL12_AP	CLK2	1.71969E-08
KLHL21_RI	CLK2	2.7607E-06
KLHL24_AT	CLK2	3.69051E-06
KLHL26_AT	CLK2	2.74305E-07
KLHL42_ES	CLK2	1.97834E-07
KLHL5_AT	CLK2	3.23213E-07

KLK6_ES	CLK2	4.84744E-12
KPNA1_ES	CLK2	6.6478E-14
KRBOX4_AT	CLK2	8.30535E-09
KRTCAP3_AP	CLK2	0.000745053
KRTCAP3_AT	CLK2	7.15972E-07
KTN1_ES	CLK2	9.02352E-13
KXD1_AP	CLK2	9.43955E-16
KXD1_AP	CLK2	0
L3HYPDH_AP	CLK2	0.005862649
L3HYPDH_AT	CLK2	0.002015262
L3MBTL2_AT	CLK2	0.023746294
LACTB_AT	CLK2	0.00033917
LAD1_AD	CLK2	5.54326E-06
LAMB1_AP	CLK2	0
LAMC2_AT	CLK2	4.39096E-05
LAMP2_AT	CLK2	3.95956E-14
LARP1B_AT	CLK2	0.002100518
LARP1B_RI	CLK2	0.001711052
LARP6_AT	CLK2	0.000683238
LAS1L_ES	CLK2	0.004898709
LAT2_AT	CLK2	1.13336E-11
LBX2_AP	CLK2	1.32439E-09
LCLAT1_AT	CLK2	0.000404378
LCORL_AT	CLK2	2.01757E-10
LDB1_AP	CLK2	2.43262E-09
LEF1_AP	CLK2	3.56734E-07
LEMD2_AP	CLK2	0.000304196
LENG8_RI	CLK2	0
LEPR_AT	CLK2	4.22324E-09
LETMD1_ES	CLK2	3.96657E-12
LETMD1_AD	CLK2	0
LETMD1_ES	CLK2	8.7647E-13
LETMD1_ES	CLK2	8.01922E-11
LETMD1_ES	CLK2	0.005264913
LETMD1_ES	CLK2	0.000315321
LETMD1_AD	CLK2	7.74663E-07
LETMD1_AD	CLK2	2.82591E-06
LGALS3BP_ES	CLK2	8.86465E-12
LGALS3BP_ES	CLK2	0.00154029
LGALS3BP_ES	CLK2	3.79149E-07
LGALS4_ES	CLK2	0.000182658
LGALS4_ES	CLK2	0.008422646
LGALS8_AT	CLK2	1.90389E-07
LGALS9C_ES	CLK2	1.68475E-12
LGMN_ES	CLK2	4.48228E-06
LHPP_AT	CLK2	0.007657828
LIG1_AD	CLK2	4.57396E-19
LIG1_ES	CLK2	0.028685063
LILRA5_AT	CLK2	0.011718729
LIMA1_AP	CLK2	0.010355949
LIMA1_AP	CLK2	9.63984E-15
LIMD1_AT	CLK2	0.03353923
LIMK2_AP	CLK2	3.4522E-10

LIMK2_AT	CLK2	1.00554E-07
LIMS1_AP	CLK2	0.000668903
LIN37_RI	CLK2	0
LIN7B_ES	CLK2	1.65428E-06
LINC00908_AT	CLK2	0.000553421
LINC00998_AD	CLK2	3.79372E-14
LIPA_AP	CLK2	0.000187615
LMBR1_AT	CLK2	3.43632E-11
LMBR1L_ES	CLK2	0
LMF1_AP	CLK2	1.05133E-07
LMF1_AT	CLK2	0.015939144
LMF2_AA	CLK2	0
LMNA_AT	CLK2	1.98621E-06
LMO7_AA	CLK2	1.25061E-11
LMO7_ES	CLK2	0.000153671
LOH12CR1_AP	CLK2	0.008199871
LPAR2_AA	CLK2	7.03382E-06
LPAR2_ES	CLK2	2.63331E-14
LPAR5_AP	CLK2	5.74103E-05
LPCAT3_RI	CLK2	0
LPCAT3_RI	CLK2	0
LRCH3_AT	CLK2	7.31134E-10
LRP11_AP	CLK2	0.004209595
LRR1_ES	CLK2	0.002385187
LRRC27_AT	CLK2	0.01401536
LRRC32_AP	CLK2	2.89185E-07
LRRC32_RI	CLK2	0.012733986
LRRC37A_AP	CLK2	3.29014E-06
LRRC37A3_AP	CLK2	0.000718295
LRRFIP2_AD	CLK2	2.01808E-08
LRSAM1_RI	CLK2	0.000281529
LRTOMT_AT	CLK2	8.92017E-05
LSP1_AP	CLK2	4.87863E-08
LTA4H_ES	CLK2	2.6808E-09
LTB4R_AP	CLK2	2.46503E-10
LTBP3_AP	CLK2	0
LTBP3_AP	CLK2	0
LTBP4_ES	CLK2	0.003015256
LTBR_ES	CLK2	0
LTN1_AT	CLK2	0.027643389
LUC7L_AP	CLK2	7.82303E-08
LUC7L_RI	CLK2	0.000120118
LUC7L_ES	CLK2	0.002203968
LUC7L_RI	CLK2	0.000102512
LUC7L_AD	CLK2	6.05504E-13
LUC7L_ES	CLK2	0.000988805
LUC7L_AD	CLK2	5.5913E-07
LUC7L3_RI	CLK2	0
LUC7L3_RI	CLK2	8.58699E-11
LUC7L3_ES	CLK2	2.03781E-07
LYPLA2_RI	CLK2	6.69969E-12
LYPLA2_RI	CLK2	6.63872E-07
LYPLA2_RI	CLK2	2.49257E-15

LYRM1_AP	CLK2	0.003310481
LYRM1_AT	CLK2	2.14223E-09
LZTS2_AP	CLK2	1.27903E-16
MAFF_AP	CLK2	5.59171E-10
MAFK_AP	CLK2	1.32485E-14
MAGI1_ES	CLK2	0.001458197
MAGOHB_ES	CLK2	6.89756E-11
MAGT1_AT	CLK2	2.10172E-09
MAN1C1_AP	CLK2	0.011205838
MAN2A2_AA	CLK2	1.4631E-11
MAN2C1_AA	CLK2	0
MAP1LC3A_AP	CLK2	0
MAP2K5_AP	CLK2	7.53141E-11
MAP2K7_AA	CLK2	0.002333993
MAP3K13_AT	CLK2	1.12769E-06
MAP3K7_ES	CLK2	0.000183049
MAP3K8_AP	CLK2	6.92592E-08
MAP4_AP	CLK2	0.013554392
MAP4_AA	CLK2	0.004361866
MAP4_ES	CLK2	0.016392344
MAP6_AT	CLK2	0.003322527
MAP7D3_AT	CLK2	7.08306E-11
MAPK9_ES	CLK2	6.71329E-13
MAPKAPK5_AD	CLK2	0.005604313
MAPRE3_AD	CLK2	2.78305E-06
MARK2_AP	CLK2	0.000223855
MARS_RI	CLK2	0
MARVELD3_AT	CLK2	0.000409219
MAST1_AT	CLK2	1.67296E-11
MAT2A_AT	CLK2	0
MATR3_AP	CLK2	2.36492E-08
MAVS_ES	CLK2	0.004844912
MAX_RI	CLK2	1.48055E-07
MAX_RI	CLK2	1.36308E-10
MAX_RI	CLK2	2.5268E-14
MAZ_AP	CLK2	1.10206E-11
MB21D1_AT	CLK2	5.14949E-05
MBD1_RI	CLK2	8.0518E-05
MBD1_AA	CLK2	0.004994603
MBD1_AA	CLK2	1.08307E-05
MBD3_AP	CLK2	3.85898E-09
MBD3_RI	CLK2	0.000876135
MBD4_AT	CLK2	0
MBLAC2_AT	CLK2	4.66226E-06
MBNL1_ES	CLK2	0.000314003
MBNL1_ES	CLK2	0.000172704
MBNL1_ES	CLK2	0.000534103
MBNL2_ES	CLK2	0.000162487
MBNL2_ES	CLK2	5.71793E-10
MBNL2_ES	CLK2	0.000119484
MBOAT7_AT	CLK2	9.30876E-14
MBTD1_AT	CLK2	0.003997444
MBTPS2_AT	CLK2	0.000206182

MCCC1_ES	CLK2	1.29299E-18
MCF2L_AP	CLK2	5.86033E-06
MCF2L_RI	CLK2	1.1039E-12
MCM7_RI	CLK2	0
MCM9_AT	CLK2	2.28729E-10
MCMDC2_AT	CLK2	1.16411E-06
MCOLN3_AT	CLK2	4.89214E-05
MCPH1_AT	CLK2	0.000650426
MCRS1_ES	CLK2	0.012560904
MDFIC_AT	CLK2	5.6536E-06
MDK_AP	CLK2	0.000110771
MDK_AD	CLK2	0.007633316
ME3_AP	CLK2	0.010232436
ME3_ES	CLK2	0
MECR_AA	CLK2	2.83453E-09
MED11_AA	CLK2	1.02301E-14
MED15_RI	CLK2	9.26349E-12
MED17_AT	CLK2	1.09059E-08
MED20_AT	CLK2	0.000265419
MED24_AA	CLK2	0.001423755
MED4_AD	CLK2	1.0676E-06
MED8_RI	CLK2	0.007947031
MED8_AA	CLK2	1.07412E-09
MEF2BNB_ES	CLK2	0
MEIS1_RI	CLK2	4.46895E-07
MEIS3_AT	CLK2	0.000514343
MELK_ES	CLK2	2.061E-06
MEPCE_AP	CLK2	0.001673604
MESDC2_AT	CLK2	0
METRNL_AP	CLK2	0
METTL15_AA	CLK2	0.002871781
METTL17_RI	CLK2	1.53043E-06
METTL17_RI	CLK2	9.73133E-11
METTL17_RI	CLK2	0
METTL17_RI	CLK2	0
METTL17_AA	CLK2	0
METTL21A_AT	CLK2	0.001191565
METTL21A_AT	CLK2	2.98505E-06
METTL3_RI	CLK2	0
METTL3_RI	CLK2	0
METTL5_AT	CLK2	0.004633778
METTL5_AD	CLK2	0.007090493
METTL6_AT	CLK2	0.000191273
MFF_ES	CLK2	0.001341166
MFF_ES	CLK2	0.001518322
MFF_ES	CLK2	0.006201929
MFI2_AT	CLK2	0.005408379
MFSD10_AP	CLK2	0
MFSD10_RI	CLK2	5.0026E-05
MFSD11_ES	CLK2	2.68954E-17
MFSD5_AP	CLK2	0.004109816
MGAT1_AP	CLK2	3.15932E-06
MGAT1_AP	CLK2	0.000152369

MGAT4A_AA	CLK2	2.41475E-05
MGAT4B_AP	CLK2	0
MGEA5_AT	CLK2	1.9757E-05
MGLL_AD	CLK2	2.77303E-07
MGRN1_RI	CLK2	0
MGRN1_AA	CLK2	0
MGST1_AP	CLK2	1.58648E-07
MIA3_AP	CLK2	7.76511E-09
MIB2_AP	CLK2	0.014254032
MIB2_RI	CLK2	7.03358E-15
MICAL3_AT	CLK2	0.001110397
MICALL2_AP	CLK2	8.03552E-12
MID1_AP	CLK2	0.000368268
MIEF1_ES	CLK2	6.11597E-08
MIER1_ES	CLK2	2.00097E-16
MINA_AD	CLK2	3.61247E-05
MINK1_AA	CLK2	0.01420147
MIPOL1_AT	CLK2	3.07788E-14
MIS12_AP	CLK2	0.001766803
MITD1_AT	CLK2	0
MKI67_ES	CLK2	0.004048989
MKL1_AP	CLK2	4.27062E-05
MKL2_AT	CLK2	0.012008424
MKNK1_AT	CLK2	2.35846E-07
MKRN1_AP	CLK2	0.000569049
MKRN1_AT	CLK2	0.015024419
MLH1_AT	CLK2	0.01199788
MLK4_AT	CLK2	0.0119064
MLLT10_AT	CLK2	4.63563E-08
MLLT3_AP	CLK2	0.00032486
MLLT3_AP	CLK2	7.91753E-10
MLLT4_AD	CLK2	0.000127466
MLPH_AP	CLK2	1.18962E-05
MLPH_ES	CLK2	0
MLST8_AP	CLK2	3.12195E-06
MLTK_AT	CLK2	0.001030299
MLXIP_AP	CLK2	1.06594E-15
MLXIPL_RI	CLK2	1.15669E-14
MMADHC_AP	CLK2	0.00422329
MMP23B_RI	CLK2	0
MMP28_AT	CLK2	0.002698116
MOB3C_AP	CLK2	0
MOGAT3_RI	CLK2	1.13068E-10
MON2_AP	CLK2	3.06833E-06
MORF4L2_AP	CLK2	0
MORF4L2_ES	CLK2	0.000556872
MORF4L2_AA	CLK2	0.005849921
MORF4L2_ES	CLK2	0.000343155
MOV10_ES	CLK2	1.09404E-07
MOXD1_AT	CLK2	5.01208E-06
MPDU1_AA	CLK2	6.30172E-10
MPHOSPH10_AT	CLK2	3.73448E-15
MPI_AT	CLK2	1.18949E-10

MPI_AT	CLK2	0
MPI_AA	CLK2	4.43556E-11
MPPE1_ES	CLK2	1.0279E-09
MPRIP_ES	CLK2	0.000517706
MPV17_AP	CLK2	0.001640381
MPV17_AT	CLK2	1.02851E-15
MPV17_AA	CLK2	0
MROH1_AT	CLK2	0.01373054
MROH1_RI	CLK2	0
MRPL10_AP	CLK2	0.00054629
MRPL2_AT	CLK2	8.92694E-06
MRPL2_ES	CLK2	0.000507377
MRPL21_RI	CLK2	0
MRPL33_ES	CLK2	3.9068E-07
MRPL34_AP	CLK2	0.000321617
MRPL35_AA	CLK2	0.00061767
MRPL4_RI	CLK2	0
MRPL43_AT	CLK2	0.000208224
MRPL43_AT	CLK2	1.43538E-05
MRPL47_ES	CLK2	0.003142774
MRPL52_ES	CLK2	0.013679507
MRPL52_ES	CLK2	6.37258E-05
MRPL52_RI	CLK2	0.001732698
MRPL52_RI	CLK2	2.42844E-05
MRPL55_AP	CLK2	4.28439E-08
MRPL55_RI	CLK2	3.76886E-12
MRPL55_RI	CLK2	0.003050396
MRPL55_RI	CLK2	3.88579E-13
MRPL55_RI	CLK2	6.67861E-15
MRPL55_AD	CLK2	5.57345E-06
MRPL55_AA	CLK2	1.30946E-12
MRPL55_ES	CLK2	0.000498701
MRPL55_ES	CLK2	4.70003E-13
MRPL55_ES	CLK2	1.81247E-05
MRPL55_ES	CLK2	0.000436727
MRPS12_AD	CLK2	0.006330204
MRPS16_AT	CLK2	0.003048455
MRPS21_AP	CLK2	4.623E-06
MRPS33_AA	CLK2	9.16642E-05
MRPS7_RI	CLK2	0
MRRF_AP	CLK2	1.41473E-11
MRRF_ES	CLK2	0
MRS2_AT	CLK2	0.000396104
MRS2_AA	CLK2	1.01078E-05
MS4A6A_AD	CLK2	0.00430034
MSANTD2_AP	CLK2	4.02847E-06
MSL3_AP	CLK2	0.000926173
MSL3_AT	CLK2	0
MSR1_AT	CLK2	8.3701E-06
MSTO1_AT	CLK2	3.51687E-12
MTA1_ES	CLK2	0.003341973
MTA2_AP	CLK2	0.001643946
MTA3_AT	CLK2	3.00588E-08

MTAP_AD	CLK2	0.001184897
MTCH1_AD	CLK2	6.41497E-06
MTERFD1_AP	CLK2	1.38058E-11
MTERFD3_RI	CLK2	2.96354E-10
MTERFD3_RI	CLK2	0
MTF2_ES	CLK2	0.002386206
MTFR1L_AP	CLK2	2.7978E-13
MTFR1L_AP	CLK2	9.49429E-10
MTFR1L_AA	CLK2	0.010968292
MTFR1L_AA	CLK2	1.74386E-15
MTFR1L_ES	CLK2	5.31092E-06
MTHFD1L_AT	CLK2	0.005342974
MTHFD2L_AT	CLK2	0.000763023
MTL5_AT	CLK2	2.53776E-06
MTMR1_ES	CLK2	5.50366E-06
MTMR10_RI	CLK2	0.003668947
MTMR11_AP	CLK2	0.002262013
MTMR4_AT	CLK2	0.001262847
MTMR7_AT	CLK2	1.05479E-05
MUC20_AP	CLK2	0
MUM1_AT	CLK2	1.00383E-10
MVD_ES	CLK2	1.78149E-05
MVK_AT	CLK2	1.03459E-08
MXD1_AA	CLK2	0
MXRA7_AT	CLK2	0.014611151
MYCL_AT	CLK2	0.000116494
MYEOV_RI	CLK2	0.005161657
MYH14_AP	CLK2	4.18139E-08
MYL12A_ES	CLK2	0.009904654
MYL5_AP	CLK2	5.21533E-12
MYL5_AD	CLK2	6.94702E-18
MYL5_AD	CLK2	0.000236679
MYL5_ES	CLK2	4.60778E-12
MYL6_ES	CLK2	0.000197294
MYL6_RI	CLK2	3.28708E-17
MYL6_RI	CLK2	1.13556E-16
MYLK_RI	CLK2	2.85126E-05
MYNN_ES	CLK2	0.002672403
MYO19_ES	CLK2	0.011192406
MYO1G_AT	CLK2	3.39503E-05
MYO5B_AP	CLK2	0.000122567
MYO5C_AT	CLK2	3.99014E-05
MYO9A_AT	CLK2	4.53283E-05
MYO9B_ES	CLK2	0.000533828
MZF1_AP	CLK2	0
MZF1_AT	CLK2	7.53475E-07
MZF1_AD	CLK2	3.22732E-09
MZT2B_ES	CLK2	7.14875E-09
NAA15_AT	CLK2	2.89688E-05
NAA35_AP	CLK2	0.003485754
NAA35_AT	CLK2	1.64167E-05
NAA38_RI	CLK2	0
NAA40_AA	CLK2	7.02351E-17

NAB1_ES	CLK2	2.78661E-07
NACA_AD	CLK2	0.011402045
NADK_AP	CLK2	9.14532E-05
NADK_AP	CLK2	0.000106956
NADSYN1_AP	CLK2	0
NADSYN1_ES	CLK2	0
NAGK_AA	CLK2	0
NAIP_AT	CLK2	0.002122723
NAP1L4_ES	CLK2	8.42469E-05
NAPRT1_RI	CLK2	1.17238E-10
NAPRT1_RI	CLK2	2.0947E-05
NAPRT1_RI	CLK2	4.09352E-08
NARF_AT	CLK2	0
NARFL_AP	CLK2	0
NARG2_AT	CLK2	1.75573E-12
NASP_ES	CLK2	0
NAT14_RI	CLK2	1.38312E-12
NAT6_AA	CLK2	2.25683E-13
NAT9_RI	CLK2	9.687E-08
NAT9_RI	CLK2	3.5302E-14
NAT9_ES	CLK2	3.81312E-07
NAT9_RI	CLK2	0
NAT9_ES	CLK2	5.93985E-07
NBN_ES	CLK2	0.004248731
NBPF1_AP	CLK2	1.18963E-07
NCAM1_AT	CLK2	0.000243168
NCAPH2_AT	CLK2	0
NCBP2_AD	CLK2	3.2277E-05
NCBP2_AD	CLK2	1.68067E-06
NCF4_RI	CLK2	0
NCK1_AP	CLK2	4.87492E-11
NCOR2_AD	CLK2	0.000876121
NDEL1_AT	CLK2	0.002013912
NDRG1_ES	CLK2	0.002728545
NDRG2_AP	CLK2	0
NDRG2_AD	CLK2	3.00295E-09
NDRG2_AD	CLK2	0
NDRG2_RI	CLK2	0
NDUFA3_AT	CLK2	1.61836E-05
NDUFA3_RI	CLK2	2.11655E-14
NDUFA7_AT	CLK2	5.19194E-05
NDUFA7_ES	CLK2	2.69315E-05
NDUFAF3_AD	CLK2	0.002297927
NDUFAF7_ES	CLK2	0.000495843
NDUFB11_AD	CLK2	9.02047E-05
NDUFB5_ES	CLK2	0.000166036
NDUFC1_AP	CLK2	9.6455E-08
NDUFS2_AT	CLK2	4.34604E-06
NDUFS2_RI	CLK2	0
NDUFS7_RI	CLK2	2.34645E-13
NDUFS7_RI	CLK2	0
NDUFS8_ES	CLK2	0.000453858
NDUFV3_ES	CLK2	1.27571E-06

NECAB3_RI	CLK2	0
NEDD4L_AP	CLK2	0.003679905
NEDD4L_AP	CLK2	6.77547E-05
NEDD9_AT	CLK2	2.29826E-05
NEIL1_AP	CLK2	1.19374E-09
NEIL3_AT	CLK2	0.004084299
NEK2_AT	CLK2	0.006621813
NEK3_AP	CLK2	2.43076E-05
NEK3_AP	CLK2	0
NEK5_AT	CLK2	0.013570244
NET1_AP	CLK2	2.23385E-10
NEU4_AP	CLK2	0.000109563
NFATC1_AT	CLK2	1.4684E-05
NFATC2IP_AP	CLK2	0
NFATC3_AT	CLK2	0.00013292
NFATC4_RI	CLK2	7.55099E-11
NFE2L1_AP	CLK2	1.14605E-09
NFIX_AP	CLK2	8.92181E-10
NFIX_AP	CLK2	0.008536641
NFYC_AP	CLK2	0.003040219
NFYC_ES	CLK2	0.004779328
NGDN_RI	CLK2	0
NGLY1_AP	CLK2	8.22126E-08
NHLRC3_ES	CLK2	0.002912529
NIF3L1_AD	CLK2	0.003400647
NIN_AT	CLK2	0.000436909
NIPA1_AP	CLK2	0.006974739
NISCH_AT	CLK2	0.001683681
NISCH_RI	CLK2	0
NIT1_AT	CLK2	1.35914E-12
NKRF_AP	CLK2	1.603E-09
NLE1_AD	CLK2	2.35098E-11
NLK_AT	CLK2	0.015347722
NLRP1_AT	CLK2	2.35529E-11
NLRP1_AT	CLK2	5.9744E-05
NMD3_AT	CLK2	0.00030489
NME4_AP	CLK2	9.39847E-11
NME4_AP	CLK2	1.62139E-06
NME4_AT	CLK2	0.000238797
NME6_AA	CLK2	3.9066E-12
NMNAT1_AT	CLK2	4.68505E-15
NMRAL1_ES	CLK2	2.20269E-13
NMRAL1_RI	CLK2	3.86018E-08
NMRAL1_RI	CLK2	4.58403E-11
NMRAL1_RI	CLK2	3.70961E-15
NMRK1_ES	CLK2	0.000113884
NMT2_RI	CLK2	8.8758E-18
NNT_AP	CLK2	0.006849771
NOL12_RI	CLK2	5.09629E-16
NOL6_AP	CLK2	7.44595E-14
NOL8_AA	CLK2	9.16233E-16
NOP2_AP	CLK2	1.9932E-13
NOP2_RI	CLK2	2.66497E-16

NOP2_RI	CLK2	3.79935E-17
NOP2_AD	CLK2	0.006810894
NOP58_ES	CLK2	4.09447E-08
NOS1AP_AP	CLK2	1.61199E-06
NOS3_AT	CLK2	0.008551693
NOTCH2NL_AT	CLK2	0.006482537
NPDC1_AP	CLK2	0
NPEPPS_AP	CLK2	0.000730479
NPEPPS_AT	CLK2	2.24885E-13
NPEPPS_AD	CLK2	0
NPEPPS_ES	CLK2	9.75651E-15
NPHP1_AT	CLK2	0.001016845
NPIPA5_AT	CLK2	0.001591092
NPIPA5_ES	CLK2	3.73039E-18
NPIPA7_AT	CLK2	9.6481E-13
NPIPB3_AP	CLK2	1.24372E-08
NPIPB4_ES	CLK2	0.006387326
NPIPB5_AP	CLK2	0.00043783
NPIPB5_ES	CLK2	0.00017292
NPIPB5_ES	CLK2	0.001931287
NPIPB6_AP	CLK2	0.014405486
NPL_AD	CLK2	1.9332E-11
NPLOC4_AP	CLK2	0.001594281
NPLOC4_AT	CLK2	0
NPM1_AT	CLK2	1.64034E-08
NPNT_ES	CLK2	1.98098E-05
NPRL2_RI	CLK2	0
NPRL2_RI	CLK2	0
NQO2_AP	CLK2	5.21711E-07
NR1H3_RI	CLK2	5.5632E-11
NR1H3_AA	CLK2	0.002170134
NR1H3_ES	CLK2	0
NR1H3_RI	CLK2	1.00148E-09
NR1H3_RI	CLK2	0
NR2C1_AT	CLK2	3.54086E-07
NR2C2AP_AT	CLK2	0.000158699
NR4A1_AT	CLK2	5.55334E-12
NR4A1_ES	CLK2	0.014759454
NR4A2_AP	CLK2	0.00332379
NRBP2_RI	CLK2	0
NRG4_AT	CLK2	1.4087E-11
NSL1_AT	CLK2	6.21703E-19
NSMCE4A_AT	CLK2	4.28293E-10
NSMCE4A_AT	CLK2	4.21683E-18
NSMF_AP	CLK2	4.61954E-06
NSMF_AT	CLK2	3.69972E-14
NSMF_RI	CLK2	0
NSMF_AA	CLK2	0
NSMF_ES	CLK2	1.32988E-06
NSUN5_RI	CLK2	8.34671E-07
NSUN5_RI	CLK2	0
NT5C_RI	CLK2	0.001202514
NT5C_RI	CLK2	6.66912E-11

NT5C_AD	CLK2	3.14944E-07
NT5C3A_AP	CLK2	3.12559E-05
NTMT1_AP	CLK2	0.001082646
NTMT1_ES	CLK2	1.49208E-05
NTNG2_AT	CLK2	4.93858E-08
NTPCR_AT	CLK2	0
NUBP2_ES	CLK2	7.16812E-17
NUCB2_AP	CLK2	0.006053707
NUDT16_RI	CLK2	7.26659E-12
NUDT16L1_RI	CLK2	9.61685E-15
NUDT16L1_AD	CLK2	0.004460689
NUDT2_ES	CLK2	7.28778E-07
NUDT4_AP	CLK2	0.001360933
NUDT5_AT	CLK2	0
NUDT5_AT	CLK2	0
NUDT8_RI	CLK2	1.94271E-07
NUP43_AT	CLK2	0.014377872
NUP62_RI	CLK2	0.010047469
NUP62_RI	CLK2	8.25327E-10
NUP85_AP	CLK2	0
NUP85_RI	CLK2	0
NUP85_ES	CLK2	2.81827E-11
NUP98_AT	CLK2	2.87649E-08
NUTM2D_AT	CLK2	0.012672227
NXF1_RI	CLK2	2.76271E-13
NXPE1_AP	CLK2	0
NXPE2_AT	CLK2	2.60922E-07
OARD1_AD	CLK2	0.013830695
OAS1_AA	CLK2	0.009800682
OAZ3_AP	CLK2	1.10787E-07
OBSL1_AT	CLK2	0.000470343
OCEL1_AP	CLK2	0.000694633
OCIAD1_AD	CLK2	1.16992E-12
OCIAD1_ES	CLK2	0.001542532
OCLN_AP	CLK2	0.006196714
ODF2_AP	CLK2	0.000735658
ODF2_AT	CLK2	1.11369E-08
ODF2_ES	CLK2	2.92475E-05
ODF3B_AA	CLK2	6.33748E-12
OFD1_AT	CLK2	0
OGDH_AT	CLK2	1.75511E-12
OGFOD2_AP	CLK2	0.014677818
OGFOD2_ES	CLK2	0
OGFOD2_ES	CLK2	1.09897E-09
OGFOD2_AA	CLK2	8.21596E-07
OGFOD2_ES	CLK2	5.20099E-12
OGFR_AP	CLK2	0
OGFR_RI	CLK2	0
OGG1_AT	CLK2	8.13946E-07
OGG1_RI	CLK2	3.27178E-07
OGG1_RI	CLK2	6.4424E-07
OLFM1_AT	CLK2	0.008611364
OLFML2B_AP	CLK2	0.0229817

OPA1_ES	CLK2	0.01028277
OPA3_AP	CLK2	0.00579489
OPA3_AT	CLK2	1.02881E-05
OPTN_ES	CLK2	0.000344672
ORAI3_AT	CLK2	3.12708E-06
ORMDL1_AP	CLK2	5.5502E-08
ORMDL1_AT	CLK2	5.71297E-09
ORMDL1_RI	CLK2	0
OS9_RI	CLK2	0
OS9_ES	CLK2	1.30284E-12
OS9_ES	CLK2	0.009791748
OSBPL1A_AP	CLK2	7.00176E-06
OSBPL1A_AT	CLK2	1.78183E-07
OSBPL3_ES	CLK2	0.001215035
OSBPL5_AP	CLK2	0
OSER1_AP	CLK2	4.59354E-07
OSGEP_AP	CLK2	0
OSGEP_AD	CLK2	0
OSGEP_RI	CLK2	0
OSGEPL1_AA	CLK2	0.006495054
OTUD5_AP	CLK2	0.00010043
OXNAD1_AT	CLK2	4.97241E-10
OXR1_AT	CLK2	6.84353E-05
OXSR1_ES	CLK2	0.000258961
P4HTM_ES	CLK2	0
PABPC1L_AT	CLK2	0.005220219
PABPC4_ES	CLK2	0.016288885
PABPC4_ES	CLK2	6.02451E-05
PABPC4_ES	CLK2	0.002246967
PABPN1_RI	CLK2	0
PACRGL_AT	CLK2	0.000569149
PACRGL_AD	CLK2	0.012987477
PACS1_AP	CLK2	2.70604E-10
PACS2_AP	CLK2	0
PACS2_AD	CLK2	0.01170455
PAIP1_AD	CLK2	5.18005E-05
PAK1_AP	CLK2	1.28373E-05
PAK4_ES	CLK2	7.89116E-05
PALLD_ES	CLK2	0.000120698
PAM16_AA	CLK2	4.88396E-09
PAN3_AP	CLK2	0.00988951
PANK1_AP	CLK2	0.002492004
PAPD4_ES	CLK2	0.000298452
PAPOLA_AT	CLK2	0.003354685
PARD3_AD	CLK2	9.14779E-08
PARD3_AD	CLK2	7.98258E-09
PARL_AD	CLK2	5.45633E-06
PARP11_AT	CLK2	0.037407043
PARP2_RI	CLK2	3.90175E-15
PARP8_AT	CLK2	0.026641842
PARP9_AT	CLK2	8.13012E-10
PASK_AT	CLK2	1.12926E-06
PAXIP1-AS2_AT	CLK2	1.56407E-06

PBRM1_ES	CLK2	5.5839E-07
PBRM1_ES	CLK2	0.000351853
PBRM1_ES	CLK2	0.000540562
PBX4_AT	CLK2	5.27751E-05
PCBD2_AT	CLK2	8.37717E-07
PCBP2_AP	CLK2	5.81521E-07
PCBP2_AT	CLK2	0
PCBP2_ES	CLK2	0.006432837
PCBP2_ES	CLK2	0.002430751
PCBP4_AA	CLK2	0.00306601
PCCB_AT	CLK2	0.00855446
PCDH11X_AT	CLK2	5.06999E-15
PCDH11Y_AT	CLK2	0
PCDH18_ES	CLK2	0.00059955
PCDH18_AD	CLK2	1.33068E-06
PCED1A_AA	CLK2	6.66801E-08
PCGF2_AP	CLK2	6.0824E-10
PCGF2_AP	CLK2	1.21497E-07
PCGF3_RI	CLK2	0.000263188
PCGF3_AA	CLK2	8.05647E-12
PCK2_AD	CLK2	2.67699E-09
PCLO_AT	CLK2	0.006666176
PCNA_AP	CLK2	0.000626259
PCNP_ES	CLK2	5.56157E-13
PCNP_ES	CLK2	0.001088622
PCNP_AA	CLK2	3.79378E-10
PCNXL2_AP	CLK2	3.81068E-06
PCNXL4_ES	CLK2	0.002010157
PCSK5_AT	CLK2	2.90027E-07
PCSK5_AT	CLK2	0.001816172
PCSK7_ES	CLK2	1.15442E-15
PCSK7_RI	CLK2	0
PCTP_AA	CLK2	9.98192E-08
PCYT1A_AT	CLK2	8.47237E-07
PCYT2_AA	CLK2	0
PDCD2_AT	CLK2	4.24664E-08
PDCD2L_AP	CLK2	0.000394227
PDCD4_ES	CLK2	1.07893E-05
PDCD6_AD	CLK2	1.28084E-05
PDDC1_AA	CLK2	7.55247E-09
PDE4D_AP	CLK2	0.000489562
PDE4DIP_AP	CLK2	0.012852509
PDE4DIP_AT	CLK2	4.74728E-16
PDE4DIP_AT	CLK2	3.37238E-11
PDE7A_AT	CLK2	9.2973E-11
PDHA1_AT	CLK2	0
PDHA1_ES	CLK2	2.15417E-05
PDHX_AP	CLK2	0.007499563
PDLIM4_ES	CLK2	5.34381E-09
PDLIM5_AT	CLK2	0.000432189
PDLIM5_AA	CLK2	0.002916923
PDLIM7_AT	CLK2	6.55409E-17
PDLIM7_AA	CLK2	0

PDP1_AP	CLK2	6.09312E-12
PDXDC1_AT	CLK2	3.08807E-05
PDXP_AP	CLK2	5.55816E-09
PDZD3_RI	CLK2	1.39875E-17
PECR_AT	CLK2	1.68779E-05
PELP1_ES	CLK2	0
PER2_AT	CLK2	3.45735E-05
PEX10_AA	CLK2	1.65996E-05
PEX11B_AP	CLK2	4.32337E-08
PEX13_AT	CLK2	0.000525102
PEX16_AP	CLK2	0.007545653
PEX19_ES	CLK2	1.68878E-08
PEX26_AT	CLK2	3.5777E-10
PEX26_RI	CLK2	0.000167586
PEX5_AD	CLK2	1.44951E-07
PFDN1_ES	CLK2	0.001016962
PFDN5_ES	CLK2	0.000256269
PFDN5_ES	CLK2	1.63642E-05
PFDN5_ES	CLK2	3.18949E-08
PFKFB2_AP	CLK2	8.74684E-08
PFKFB3_AT	CLK2	0.02269632
PFKM_RI	CLK2	0
PFKP_AP	CLK2	0
PGAM5_AP	CLK2	9.26474E-06
PGAP1_AT	CLK2	0.043497697
PGAP2_AD	CLK2	1.83065E-11
PGAP2_AD	CLK2	1.85574E-15
PGAP2_ES	CLK2	4.75944E-14
PGAP2_ES	CLK2	0.000205924
PGBD2_AA	CLK2	5.9195E-05
PGPEP1_ES	CLK2	8.92314E-05
PHB2_ES	CLK2	0
PHC2_AP	CLK2	3.83546E-05
PHF12_RI	CLK2	6.54482E-15
PHF12_RI	CLK2	0
PHF12_RI	CLK2	0
PHF14_ES	CLK2	0.010126572
PHF19_AP	CLK2	8.30241E-06
PHF6_AT	CLK2	6.19418E-05
PHLDB3_AT	CLK2	0.000278724
PHLPP2_AT	CLK2	6.78926E-07
PHTF2_AT	CLK2	0.001662442
PHYKPL_AT	CLK2	7.7936E-10
PHYKPL_ES	CLK2	7.6943E-11
PIAS2_AT	CLK2	0.001099475
PID1_AP	CLK2	0
PID1_ES	CLK2	1.23832E-13
PIDD_AP	CLK2	1.84522E-06
PIDD_RI	CLK2	4.00128E-17
PIDD_AA	CLK2	1.29816E-10
PIGA_ES	CLK2	4.1882E-08
PIGG_AT	CLK2	2.92516E-10
PIGG_AD	CLK2	0.000925838

PIGH_AT	CLK2	0.000285801
PIGL_AT	CLK2	2.75742E-09
PIGT_AD	CLK2	0.00427633
PIGV_AP	CLK2	0.000222226
PIGV_AD	CLK2	0.00034225
PIGX_AP	CLK2	0.010588628
PIGX_AT	CLK2	0.016386672
PIK3C3_AT	CLK2	2.02396E-08
PILRB_AP	CLK2	0.006856044
PILRB_AA	CLK2	2.34348E-05
PILRB_ES	CLK2	0.001325159
PITPNM2_AT	CLK2	4.70709E-05
PITX2_AT	CLK2	6.64155E-07
PIWIL4_AP	CLK2	1.64133E-07
PKMYT1_AP	CLK2	0
PKMYT1_AT	CLK2	0.009412337
PKN1_AP	CLK2	2.41506E-05
PKN2_AT	CLK2	1.662E-07
PLA2G10_ES	CLK2	1.97616E-12
PLA2R1_AT	CLK2	6.89875E-05
PLAGL1_RI	CLK2	0
PLAUR_AT	CLK2	5.19669E-08
PLAUR_ES	CLK2	0.003318497
PLCB3_AT	CLK2	7.41094E-05
PLCD1_AP	CLK2	0.004967232
PLCH1_AT	CLK2	1.09872E-06
PLCH2_AT	CLK2	1.28486E-13
PLD2_RI	CLK2	0
PLD3_ES	CLK2	0.00621136
PLEC_AP	CLK2	3.11558E-06
PLEKHA5_ES	CLK2	0.001434538
PLEKHA6_AA	CLK2	9.91488E-06
PLEKHA7_AT	CLK2	1.77516E-05
PLEKHA8_AT	CLK2	7.73693E-09
PLEKHB2_AP	CLK2	2.34047E-07
PLEKHB2_ES	CLK2	3.14612E-05
PLEKHB2_ES	CLK2	0.000574254
PLEKHB2_AD	CLK2	0.000202946
PLEKHG4_RI	CLK2	0
PLEKHG6_AP	CLK2	3.40891E-13
PLEKHJ1_AT	CLK2	0
PLEKHJ1_RI	CLK2	0
PLEKHS1_AP	CLK2	0
PLEKHS1_AT	CLK2	0.006553115
PLS1_AP	CLK2	3.8317E-12
PLSCR1_AT	CLK2	1.03044E-08
PLXNC1_AT	CLK2	3.85144E-06
PML_AT	CLK2	0.001420594
PML_RI	CLK2	8.8772E-07
PMP22_AP	CLK2	1.50812E-07
PMPCB_AT	CLK2	8.32942E-06
PMPCB_RI	CLK2	1.18392E-12
PMS1_AT	CLK2	0.015377162

PNKD_AP	CLK2	3.88625E-13
PNKD_AT	CLK2	5.5994E-14
PNKP_AP	CLK2	0.000879669
PNKP_AA	CLK2	6.11992E-08
PODNL1_AP	CLK2	0.000200103
PODXL_RI	CLK2	0
POFUT2_AD	CLK2	0
POFUT2_ES	CLK2	2.08967E-10
POGK_RI	CLK2	3.92252E-05
POLA2_AT	CLK2	0
POLB_AP	CLK2	1.30318E-10
POLD4_AD	CLK2	5.01895E-09
POLD4_AD	CLK2	2.98546E-06
POLH_AT	CLK2	0.006996867
POLL_RI	CLK2	0
POLL_AD	CLK2	2.1959E-06
POLL_ES	CLK2	6.50981E-05
POLL_RI	CLK2	0.00026054
POLM_AA	CLK2	0.002780806
POLM_RI	CLK2	6.39887E-06
POLM_AD	CLK2	1.77068E-08
POLM_RI	CLK2	1.33696E-07
POLM_RI	CLK2	0.00116127
POLM_ES	CLK2	0.000151261
POLR2G_AD	CLK2	4.69039E-06
POLR2H_AA	CLK2	0.012053337
POLR2H_ES	CLK2	2.01345E-05
POLR2J2_ES	CLK2	0.006493372
POLR2J3_AT	CLK2	1.08046E-12
POLR2J3_AT	CLK2	0.004585493
POLR2J3_RI	CLK2	7.11339E-12
POLR2J3_ES	CLK2	0
POLR2J3_ES	CLK2	0.013849659
POM121_AT	CLK2	0
POM121C_AP	CLK2	5.82502E-06
POMGNT1_RI	CLK2	0
POMGNT1_RI	CLK2	0
POMT1_AD	CLK2	5.97579E-13
POMT1_ES	CLK2	0.005101898
POMZP3_ES	CLK2	2.8603E-05
PON2_AD	CLK2	0
PPAPDC1B_AD	CLK2	6.72163E-06
PPARD_AT	CLK2	0.000606093
PPCS_AD	CLK2	4.60007E-09
PPFIA1_AT	CLK2	3.26451E-13
PPFIBP2_AP	CLK2	0.005500527
PPHLN1_AT	CLK2	4.24629E-12
PPIA_ES	CLK2	5.79707E-16
PPIE_AD	CLK2	0.001783653
PPM1A_AT	CLK2	0.010843676
PPM1B_AA	CLK2	4.19956E-13
PPM1F_AT	CLK2	1.30202E-05
PPM1M_RI	CLK2	0

PPM1N_AP	CLK2	8.88718E-14
PPP1CC_RI	CLK2	0
PPP1R14A_AP	CLK2	0.000254603
PPP1R14B_AP	CLK2	0.004282847
PPP1R1B_AP	CLK2	5.57775E-08
PPP1R7_AT	CLK2	2.13136E-06
PPP1R7_ES	CLK2	7.92877E-05
PPP1R8_AD	CLK2	0.007827876
PPP2R1B_ES	CLK2	0.0015127
PPP2R4_AP	CLK2	3.18183E-07
PPP2R5C_AT	CLK2	0.011833374
PPP3CB_ES	CLK2	0.00184958
PPP4C_ES	CLK2	9.3465E-15
PPP4R1_ES	CLK2	1.12379E-05
PPP4R1L_AT	CLK2	0.007734807
PPP4R2_AT	CLK2	0.003119552
PPRC1_ES	CLK2	0.000446619
PQLC1_ES	CLK2	0.003419312
PQLC2_RI	CLK2	0.010765086
PQLC3_ES	CLK2	5.80495E-08
PRAP1_AD	CLK2	0.004266262
PRDM5_AT	CLK2	1.18225E-07
PRICKLE3_AT	CLK2	0
PRKAB1_AP	CLK2	0.000574926
PRKACB_AP	CLK2	1.28424E-08
PRKAG1_AD	CLK2	2.55639E-05
PRKCA_AT	CLK2	2.83925E-10
PRKCB_AA	CLK2	1.64032E-18
PRKCSH_AP	CLK2	0.002844649
PRKCZ_AP	CLK2	0.009930657
PRKD2_AP	CLK2	7.54824E-09
PRKD2_AD	CLK2	0.001381459
PRMT2_AT	CLK2	0.000658452
PRMT2_RI	CLK2	0
PRMT2_AD	CLK2	1.70416E-13
PROM1_AP	CLK2	0.00097818
PROM2_AA	CLK2	0.000246193
PRPF38B_AT	CLK2	2.79256E-11
PRPF39_ES	CLK2	3.24712E-17
PRPF39_ES	CLK2	7.8048E-19
PRPF8_RI	CLK2	0.000738433
PRR13_AA	CLK2	0.000526042
PRR13_AA	CLK2	0.001053733
PRR13_AA	CLK2	0.001014047
PRR13_AD	CLK2	0.000621814
PRRG1_AT	CLK2	1.87311E-12
PRRG2_AA	CLK2	1.21755E-05
PRRX1_AT	CLK2	2.08398E-06
PRSS23_AT	CLK2	0.003319807
PRSS23_RI	CLK2	0.001986028
PSEN1_AD	CLK2	0.008270429
PSEN1_AD	CLK2	0.011692823
PSENEN_AA	CLK2	3.83759E-05

PSIP1_AT	CLK2	0.001216439
PSMA2_AT	CLK2	3.27423E-08
PSMC5_RI	CLK2	0
PSMD6_ES	CLK2	2.83241E-07
PSMD8_AT	CLK2	3.80135E-20
PSME1_RI	CLK2	0
PSME2_AA	CLK2	4.09549E-13
PSME3_AP	CLK2	0.002062966
PSMF1_AA	CLK2	0.004130222
PSMG3_AP	CLK2	0.000181154
PSMG4_RI	CLK2	2.64282E-10
PSMG4_RI	CLK2	0
PSMG4_AA	CLK2	4.83786E-13
PSPC1_AT	CLK2	0.001466199
PSPC1_AT	CLK2	0.007311295
PSPH_AP	CLK2	0.000439704
PTAR1_AT	CLK2	7.49778E-09
PTCD2_AT	CLK2	3.03964E-07
PTCH1_AP	CLK2	0.016052525
PTCH2_AT	CLK2	0.000460078
PTGES2_ES	CLK2	0
PTGES2_AD	CLK2	0.004499189
PTGR1_AA	CLK2	0
PTK2_RI	CLK2	4.11826E-14
PTK2B_RI	CLK2	0
PTK7_AP	CLK2	3.93043E-15
PTOV1_RI	CLK2	0
PTOV1_RI	CLK2	0
PTPLA_AT	CLK2	0.008509898
PTPN2_AT	CLK2	0.008283641
PTPN3_AP	CLK2	1.62275E-17
PTPN6_AA	CLK2	0
PTPRC_AT	CLK2	5.80759E-05
PTPRE_AP	CLK2	0.001126284
PTPRE_AD	CLK2	0
PTPRJ_AT	CLK2	2.17923E-08
PTPRK_AT	CLK2	2.13183E-12
PTPRO_RI	CLK2	3.6679E-09
PTRH2_AP	CLK2	0
PTRH2_ES	CLK2	1.81915E-06
PTS_AP	CLK2	0.017820707
PTS_ES	CLK2	1.27887E-05
PTTG1_AP	CLK2	2.91455E-10
PUM2_AD	CLK2	0.00340876
PUM2_ES	CLK2	0.000960608
PVR_ES	CLK2	0.001241332
PVRL1_AT	CLK2	4.5858E-06
PVRL2_AT	CLK2	0.009732641
PVRL3_AT	CLK2	1.06519E-06
PVRL3_AA	CLK2	1.71283E-15
PXK_AT	CLK2	1.23607E-15
PXMP2_AP	CLK2	0
PXMP2_AT	CLK2	8.46712E-08

PXN_AP	CLK2	0.002644863
PXN_AA	CLK2	0.002030534
PYCR1_AP	CLK2	0.000236351
PYGO2_AP	CLK2	0.008809882
QKI_AT	CLK2	9.51627E-05
QKI_RI	CLK2	0.00856269
QTRT1_RI	CLK2	0
R3HDM4_ES	CLK2	0
R3HDM4_ES	CLK2	1.03096E-08
RAB11B_AT	CLK2	1.25663E-12
RAB15_AD	CLK2	0
RAB15_AD	CLK2	0.005606233
RAB34_AP	CLK2	0.011145383
RAB3GAP1_AT	CLK2	0.000473448
RAB3GAP1_ES	CLK2	5.31473E-09
RAB3IP_AP	CLK2	1.87856E-15
RAB6A_ME	CLK2	7.84675E-07
RABEPK_AT	CLK2	8.10091E-05
RABGGTA_RI	CLK2	0.014578011
RABGGTB_AT	CLK2	3.38445E-11
RABGGTB_ES	CLK2	0
RABL2B_AT	CLK2	0
RABL2B_AD	CLK2	3.30494E-10
RABL2B_AA	CLK2	6.32167E-06
RABL2B_ES	CLK2	4.98234E-17
RABL2B_ES	CLK2	3.41231E-16
RABL2B_ES	CLK2	0.002031026
RAC1_ES	CLK2	5.62108E-13
RACGAP1_AD	CLK2	0.001204752
RAD1_AP	CLK2	3.56543E-06
RAD21_AP	CLK2	7.34895E-15
RAD21_AP	CLK2	1.05435E-11
RAD23B_AP	CLK2	2.01941E-05
RAD51B_AT	CLK2	0.002053646
RAD51C_AT	CLK2	0.000239717
RAD52_AT	CLK2	5.25959E-12
RAE1_AT	CLK2	7.37159E-14
RAF1_ES	CLK2	0.015516571
RALBP1_AP	CLK2	0.002256119
RALGDS_AP	CLK2	0.000235909
RALY_ES	CLK2	0.001313396
RAMP1_AP	CLK2	4.84144E-05
RAN_AD	CLK2	1.11313E-09
RANBP1_AP	CLK2	0.006226499
RANBP1_RI	CLK2	0
RANBP1_RI	CLK2	0
RANBP10_AT	CLK2	0
RANBP10_AA	CLK2	0.008604484
RANBP3_ME	CLK2	1.93E-06
RANBP3_AA	CLK2	0
RANBP3_ES	CLK2	6.43463E-06
RANBP3_ES	CLK2	3.95135E-10
RANGAP1_AP	CLK2	2.2688E-07

RANGAP1_AP	CLK2	0
RANGRF_RI	CLK2	2.4442E-08
RAP1B_ES	CLK2	1.03722E-09
RAPH1_AT	CLK2	0.01109311
RAPH1_AT	CLK2	3.91067E-07
RARA_AP	CLK2	8.74248E-10
RARG_AP	CLK2	0.001694443
RARRES2_RI	CLK2	0
RARRES2_RI	CLK2	4.32241E-12
RASA4_ES	CLK2	0.003487569
RASA4B_ES	CLK2	0.007071373
RASAL2_AP	CLK2	1.75213E-07
RASEF_AT	CLK2	5.36585E-16
RASGEF1B_AT	CLK2	1.83501E-08
RASSF5_AP	CLK2	7.22904E-08
RASSF7_RI	CLK2	2.52517E-06
RASSF7_RI	CLK2	2.26786E-11
RASSF7_RI	CLK2	9.03922E-08
RAVER2_ES	CLK2	3.10287E-08
RAVER2_AA	CLK2	2.08639E-08
RBBP8_AP	CLK2	0.000175627
RBCK1_AT	CLK2	3.40308E-06
RBCK1_ES	CLK2	0.004782679
RBFOX2_AP	CLK2	0.000186044
RBM14-RBM4_ES	CLK2	0.010309381
RBM17_AP	CLK2	3.51147E-07
RBM25_AT	CLK2	4.80194E-14
RBM3_RI	CLK2	0
RBM33_AT	CLK2	1.34654E-09
RBM38_AP	CLK2	0.000757371
RBM39_ES	CLK2	0.00011699
RBM39_ES	CLK2	8.05799E-12
RBM39_ES	CLK2	1.16453E-13
RBM4_AP	CLK2	4.58368E-05
RBM4_AT	CLK2	3.81609E-11
RBM4_AD	CLK2	7.02091E-08
RBM47_AP	CLK2	2.22743E-08
RBM47_AP	CLK2	4.86249E-10
RBM48_RI	CLK2	0
RBM4B_AP	CLK2	0.000114518
RBM5_AT	CLK2	2.01512E-06
RBM6_RI	CLK2	0
RBM6_ES	CLK2	0.001372445
RBM6_ES	CLK2	4.13829E-08
RBM6_ES	CLK2	2.0795E-14
RBM6_ES	CLK2	5.02568E-09
RBM6_AA	CLK2	3.98034E-05
RBMX_RI	CLK2	1.35771E-16
RBP5_AT	CLK2	4.43027E-07
RCAN1_AP	CLK2	2.23762E-10
RCAN1_ES	CLK2	2.00531E-12
RCBTB1_AT	CLK2	1.47643E-10
RCE1_AP	CLK2	0.003409062

RCOR3_AT	CLK2	1.83766E-05
RCOR3_ES	CLK2	4.20914E-08
RCOR3_ES	CLK2	2.86735E-15
RDH13_AT	CLK2	0.003926874
RECQL5_AT	CLK2	2.08295E-14
RELA_RI	CLK2	0
RELL1_AT	CLK2	2.35557E-06
RELL2_RI	CLK2	0
REPIN1_AA	CLK2	0.001526112
REPIN1_AD	CLK2	0.007880766
REPS1_ES	CLK2	2.7176E-05
RER1_AP	CLK2	0
RET_AT	CLK2	0.015437551
REV1_RI	CLK2	2.5895E-06
RFC4_AD	CLK2	0.000639767
RFC5_ES	CLK2	1.91259E-11
RFC5_ES	CLK2	0.011111141
RFNG_AP	CLK2	0.000750791
RGL3_AT	CLK2	0.000255804
RGMB_AP	CLK2	0.012850633
RGS1_AT	CLK2	4.35602E-17
RGS10_AP	CLK2	0.00081917
RGS11_AT	CLK2	2.2201E-18
RHBDD2_ES	CLK2	0.000797294
RHBDF1_AT	CLK2	0
RHBDF1_RI	CLK2	0
RHBDL2_AP	CLK2	6.07094E-06
RHOA_ES	CLK2	0.012135112
RHOC_AA	CLK2	0
RHOF_AT	CLK2	3.41388E-16
RHOT2_RI	CLK2	0
RIC8B_AP	CLK2	1.19902E-07
RIMKLB_AT	CLK2	6.2106E-08
RIOK2_AT	CLK2	4.87084E-05
RIPK3_AD	CLK2	0
RMDN2_AT	CLK2	2.51536E-13
RMND1_AD	CLK2	3.45111E-10
RMND5B_AD	CLK2	0.012588396
RNASE1_ES	CLK2	0.007015194
RNASE1_ES	CLK2	2.4001E-05
RNASE1_ES	CLK2	2.65331E-06
RNASEH2C_RI	CLK2	6.00135E-06
RNASEL_AT	CLK2	0
RNF121_ES	CLK2	0.000297642
RNF123_AD	CLK2	0
RNF13_AD	CLK2	0.012484553
RNF13_ES	CLK2	2.31402E-05
RNF14_ES	CLK2	0.000975667
RNF14_ES	CLK2	4.03451E-05
RNF14_ES	CLK2	4.62648E-07
RNF146_AT	CLK2	1.82143E-09
RNF149_AT	CLK2	0
RNF166_AP	CLK2	1.20367E-13

RNF166_RI	CLK2	0
RNF167_RI	CLK2	0.009624214
RNF170_AD	CLK2	0.000101685
RNF213_AT	CLK2	0.001304269
RNF220_AT	CLK2	9.56457E-13
RNF220_AD	CLK2	0.006653394
RNF31_AD	CLK2	4.24445E-05
RNF32_AT	CLK2	0.001791033
RNF34_AT	CLK2	0.004270151
RNF38_AP	CLK2	0.004754
RNF4_AP	CLK2	7.86468E-06
RNF43_AT	CLK2	0
RNF44_AA	CLK2	0.012031408
RNF7_ES	CLK2	1.85725E-11
RNF7_ES	CLK2	3.59048E-05
RNF8_ES	CLK2	0.001806431
RNGTT_AT	CLK2	0.018754596
RNH1_AP	CLK2	0.005240318
RNH1_ES	CLK2	0.002923308
RNH1_ES	CLK2	0.002469191
RNMTL1_ES	CLK2	5.11109E-06
RNPC3_AA	CLK2	0
RNPS1_AD	CLK2	0
RNPS1_AD	CLK2	0.000117763
ROGDI_AA	CLK2	8.41856E-10
ROGDI_AD	CLK2	1.86994E-19
RPAIN_AT	CLK2	0
RPAP1_RI	CLK2	1.57798E-10
RPL10_RI	CLK2	0.002468016
RPL13_RI	CLK2	2.54099E-05
RPL13_AD	CLK2	3.65178E-06
RPL14_AD	CLK2	4.98272E-10
RPL15_AD	CLK2	0.002201027
RPL17_AP	CLK2	0.00512098
RPL17_AD	CLK2	0.005025233
RPL18A_ES	CLK2	0.001248881
RPL19_ES	CLK2	0.000250204
RPL21_AD	CLK2	3.01037E-13
RPL26L1_AP	CLK2	9.97282E-05
RPL26L1_AD	CLK2	0.000823482
RPL28_AT	CLK2	0
RPL28_AT	CLK2	5.18324E-11
RPL28_RI	CLK2	6.55143E-17
RPL29_RI	CLK2	0.003497347
RPL32_RI	CLK2	6.09997E-13
RPL32_RI	CLK2	0
RPL34_AT	CLK2	9.84854E-08
RPL35_AD	CLK2	0
RPL37A_RI	CLK2	8.69862E-11
RPL39L_AP	CLK2	1.28338E-09
RPL6_AD	CLK2	7.98333E-06
RPL6_ES	CLK2	4.60283E-06
RPL8_RI	CLK2	0.00089822

RPLP0_AA	CLK2	0.000301561
RPLP0_AA	CLK2	0.001617172
RPP25L_AD	CLK2	0.004068373
RPP38_ES	CLK2	0.006245905
RPS11_ES	CLK2	0.011003201
RPS15_AA	CLK2	3.40991E-05
RPS15_ES	CLK2	0.000206758
RPS15_ES	CLK2	0.000746578
RPS15A_ES	CLK2	0.00044996
RPS15A_AD	CLK2	8.24076E-11
RPS2_RI	CLK2	0.000332811
RPS20_AA	CLK2	1.22647E-13
RPS21_AA	CLK2	0
RPS21_AD	CLK2	0
RPS25_ES	CLK2	0.001411592
RPS27A_RI	CLK2	0.000528267
RPS27L_AT	CLK2	0
RPS3_AT	CLK2	0.000868342
RPS3A_ES	CLK2	0
RPS3A_ES	CLK2	0
RPS6_AA	CLK2	7.97342E-18
RPS6_AD	CLK2	0
RPS6_RI	CLK2	0.000154653
RPS9_RI	CLK2	3.08052E-12
RPS9_AA	CLK2	0
RPS9_ES	CLK2	0.01370985
RPUSD4_ES	CLK2	0.000713876
RRNAD1_RI	CLK2	0.00097129
RRP8_RI	CLK2	4.68121E-08
RRP8_AA	CLK2	3.93454E-08
RSAD1_AD	CLK2	0
RSPRY1_AD	CLK2	2.85098E-08
RTEL1_AD	CLK2	5.49089E-05
RTKN2_AT	CLK2	8.59945E-08
RTKN2_AT	CLK2	2.44384E-07
RTN4_AP	CLK2	3.06379E-13
RUFY1_AP	CLK2	0.002745143
RUFY1_ES	CLK2	7.48655E-06
RUNX1_ES	CLK2	0.00118565
RWDD1_ES	CLK2	0.004956694
S100A1_AA	CLK2	0.000443537
S100A13_AP	CLK2	7.59682E-05
S100A16_AD	CLK2	0.002511843
S100A2_AP	CLK2	1.64006E-05
S100A4_AP	CLK2	4.04217E-05
S100PBP_AT	CLK2	0.006074297
SAA2_AT	CLK2	0.001970778
SAFB2_AT	CLK2	1.77914E-17
SAMHD1_AT	CLK2	0.008014591
SAP30BP_ES	CLK2	9.59392E-09
SAR1B_AP	CLK2	2.90648E-13
SARNP_AT	CLK2	8.44594E-05
SAT1_AT	CLK2	0

SATB2_AP	CLK2	0.00013856
SAYSD1_AP	CLK2	0.000276234
SCAF11_AP	CLK2	4.86302E-07
SCARB1_AT	CLK2	6.65863E-09
SCIMP_AT	CLK2	1.5954E-07
SCLY_AT	CLK2	0.000267181
SCMH1_ES	CLK2	0.004445573
SCNM1_AP	CLK2	0.001768599
SCNN1A_AP	CLK2	0.004051893
SCNN1A_ES	CLK2	5.19927E-07
SCO2_AP	CLK2	0.000517206
SCP2_ES	CLK2	6.48722E-08
SCRN2_AP	CLK2	2.28406E-05
SCRN2_RI	CLK2	0
SCRN2_AA	CLK2	0
SCYL1_RI	CLK2	8.75946E-08
SCYL1_AA	CLK2	3.44219E-09
SCYL3_ES	CLK2	0.000598858
SDC2_AD	CLK2	4.26304E-08
SDC3_RI	CLK2	0
SDCBP_ES	CLK2	1.33456E-07
SDCCAG3_ES	CLK2	0.001810636
SDCCAG3_ES	CLK2	6.06451E-05
SDHAF2_ES	CLK2	1.70735E-05
SDR39U1_AP	CLK2	0
SEC14L1_AP	CLK2	0.007014582
SEC14L2_AT	CLK2	1.95487E-13
SEC16A_AA	CLK2	0.000603158
SEC22C_AT	CLK2	2.29192E-10
SEC23A_AP	CLK2	0.001070344
SEC23A_AT	CLK2	2.98025E-09
SEC24A_AT	CLK2	9.45905E-10
SEC24B_ES	CLK2	0.000367009
SEC24C_RI	CLK2	0
SEC31A_AP	CLK2	5.0959E-05
SEC31A_AD	CLK2	6.67484E-11
SEC31A_ES	CLK2	7.77559E-12
SEC31A_ES	CLK2	0.003955184
SEC61A2_AT	CLK2	3.17108E-16
SEC61G_AD	CLK2	0.012804148
SECTM1_AA	CLK2	5.93507E-14
SEMA4B_AP	CLK2	0.00017209
SEMA4G_AD	CLK2	0.004129224
SEMA6A_AP	CLK2	1.42331E-12
SEMA6A_ES	CLK2	3.66298E-06
SENP6_ES	CLK2	8.76591E-05
SEPHS1_ES	CLK2	2.61644E-05
SEPT10_AT	CLK2	0.00025447
SEPT2_AP	CLK2	0.009593305
SEPT2_ES	CLK2	0.000754303
SEPT6_AT	CLK2	2.21931E-09
SEPT8_AA	CLK2	1.29774E-16
SEPT9_AP	CLK2	0.00120318

SERF1A_AP	CLK2	0.013628468
SERHL2_AD	CLK2	0.00012938
SERINC2_AP	CLK2	9.36528E-11
SERP2_ES	CLK2	6.83009E-05
SERPINA1_RI	CLK2	0.000584287
SERPINA1_RI	CLK2	0.000101968
SERPINA1_AA	CLK2	6.65497E-05
SERPINA1_AA	CLK2	3.19528E-06
SERPINA1_ES	CLK2	0.001842907
SERPINA1_ES	CLK2	1.60927E-06
SERPINA1_ES	CLK2	0.001512324
SERPINA1_ES	CLK2	0.005593325
SERPINB6_AP	CLK2	0.000688634
SERPINB6_AP	CLK2	0
SERPING1_AP	CLK2	0.01037054
SERTAD3_AP	CLK2	2.21285E-05
SESN1_AP	CLK2	1.19012E-06
SET_AP	CLK2	0.000602728
SETD4_AP	CLK2	3.63315E-08
SETD4_AT	CLK2	0
SETD6_AA	CLK2	1.40163E-11
SETDB1_AT	CLK2	1.28227E-09
SETMAR_AT	CLK2	2.9559E-06
SF1_AA	CLK2	0.000126681
SF1_AD	CLK2	0.002675995
SF3B1_AT	CLK2	1.3725E-05
SF3B1_AD	CLK2	0.003468139
SFMBT2_AT	CLK2	1.87708E-05
SFSWAP_ES	CLK2	3.20389E-09
SFSWAP_AD	CLK2	0.003074188
SFXN5_AT	CLK2	3.31552E-07
SGCE_ES	CLK2	0.002516637
SGK1_AP	CLK2	1.06629E-06
SGK2_AP	CLK2	0
SGK2_ES	CLK2	0
SGSH_AT	CLK2	2.44137E-13
SGSM3_RI	CLK2	0
SH2B1_AP	CLK2	7.49174E-08
SH2B1_AD	CLK2	4.98468E-07
SH2B1_RI	CLK2	2.48343E-17
SH2B1_RI	CLK2	0
SH2D3A_RI	CLK2	5.2104E-08
SH3BP1_ES	CLK2	2.144E-07
SH3BP2_AP	CLK2	4.78366E-05
SH3BP2_ES	CLK2	4.03253E-07
SH3KBP1_AP	CLK2	0.000141676
SH3RF2_AT	CLK2	1.76812E-07
SH3YL1_AP	CLK2	0.000358244
SH3YL1_AP	CLK2	0
SH3YL1_ES	CLK2	0.004579678
SH3YL1_AD	CLK2	3.58008E-09
SHC1_AP	CLK2	0.005993264
SHF_AP	CLK2	0.000435482

SHH_AP	CLK2	0.000181514
SHPK_AT	CLK2	0.000227773
SHPK_AT	CLK2	0.000172489
SHROOM1_AA	CLK2	0
SHROOM1_ES	CLK2	3.68215E-15
SIAH1_AP	CLK2	1.08021E-07
SIAH1_AT	CLK2	0.000318125
SIDT2_RI	CLK2	3.52835E-11
SIDT2_RI	CLK2	0
SIGIRR_AP	CLK2	0.000199657
SIGIRR_RI	CLK2	0
SIMC1_AT	CLK2	7.71661E-16
SIN3B_AT	CLK2	1.56888E-09
SIN3B_ES	CLK2	0.002371094
SIRT3_AA	CLK2	1.65745E-08
SIRT5_AT	CLK2	2.16628E-10
SIRT6_AA	CLK2	0.000779906
SLAMF7_ES	CLK2	5.18378E-12
SLC10A3_RI	CLK2	0.000177689
SLC10A3_RI	CLK2	0.000274686
SLC11A2_AT	CLK2	0.006429651
SLC12A2_ES	CLK2	0.002606573
SLC12A9_AT	CLK2	0.000940975
SLC12A9_AA	CLK2	0
SLC13A3_AT	CLK2	3.03083E-05
SLC14A2_AT	CLK2	4.77238E-05
SLC15A4_AP	CLK2	1.31065E-10
SLC15A4_AP	CLK2	9.69568E-06
SLC15A4_RI	CLK2	2.59567E-12
SLC16A1_AT	CLK2	7.18942E-10
SLC16A3_AP	CLK2	4.85525E-10
SLC16A5_AP	CLK2	0.006689799
SLC16A5_AP	CLK2	7.76212E-15
SLC16A5_AT	CLK2	6.39971E-06
SLC19A1_AP	CLK2	5.73265E-05
SLC1A4_AP	CLK2	0.00503791
SLC20A2_AP	CLK2	1.85592E-05
SLC22A18_AP	CLK2	1.18453E-09
SLC22A18_AP	CLK2	0.001559978
SLC22A18AS_ES	CLK2	2.34794E-14
SLC22A23_AT	CLK2	0.012367062
SLC24A1_AT	CLK2	1.62322E-07
SLC25A14_RI	CLK2	0
SLC25A16_ES	CLK2	0
SLC25A19_AP	CLK2	0
SLC25A22_AP	CLK2	2.75564E-08
SLC25A26_AT	CLK2	2.95111E-10
SLC25A29_AP	CLK2	2.18992E-12
SLC25A29_AA	CLK2	1.66153E-14
SLC25A29_AA	CLK2	7.26961E-07
SLC25A3_AD	CLK2	2.14272E-19
SLC25A35_AT	CLK2	1.19862E-12
SLC25A37_AA	CLK2	1.05979E-05

SLC25A37_AD	CLK2	0.006464596
SLC25A39_AA	CLK2	1.84457E-07
SLC26A1_AT	CLK2	0.00216222
SLC26A6_AP	CLK2	0
SLC29A1_AP	CLK2	0.00016851
SLC29A1_ES	CLK2	0.000114519
SLC29A1_ES	CLK2	1.30022E-06
SLC2A11_AT	CLK2	6.58619E-08
SLC30A5_AA	CLK2	0.008168979
SLC35A2_AT	CLK2	1.26649E-05
SLC35B1_AA	CLK2	0.003873347
SLC35C1_RI	CLK2	2.43205E-12
SLC35C2_AA	CLK2	1.12948E-06
SLC35D1_AT	CLK2	2.30884E-11
SLC35E2_AT	CLK2	6.2408E-06
SLC35F5_AT	CLK2	3.57836E-06
SLC35F5_AD	CLK2	2.61766E-12
SLC35G1_AT	CLK2	4.12318E-12
SLC37A3_AA	CLK2	0
SLC37A4_ES	CLK2	1.94737E-09
SLC39A1_AP	CLK2	0.001096782
SLC39A13_RI	CLK2	0
SLC39A13_RI	CLK2	0
SLC39A13_RI	CLK2	0
SLC39A14_AP	CLK2	7.12038E-13
SLC39A5_AP	CLK2	0.002450579
SLC39A8_AP	CLK2	5.32357E-06
SLC3A1_AP	CLK2	2.97848E-17
SLC3A2_AP	CLK2	4.14952E-05
SLC3A2_ES	CLK2	4.79441E-05
SLC44A1_AT	CLK2	1.11584E-05
SLC44A5_AT	CLK2	9.93102E-14
SLC4A8_AT	CLK2	0.004865811
SLC52A1_AP	CLK2	8.14773E-11
SLC52A2_AD	CLK2	0.00042621
SLC52A2_AD	CLK2	0.002538438
SLC52A3_RI	CLK2	0.000229006
SLC6A6_AT	CLK2	0.000111545
SLC6A8_AP	CLK2	0
SLC6A9_AT	CLK2	2.54943E-13
SLC8B1_AP	CLK2	1.70356E-16
SLC8B1_AP	CLK2	3.31191E-10
SLCO2B1_RI	CLK2	0
SLCO3A1_AT	CLK2	0.009413844
SLCO4A1_AD	CLK2	4.43523E-18
SLCO4A1_RI	CLK2	0
SLIRP_AT	CLK2	0.009224871
SLTM_ES	CLK2	0.004737785
SMAD2_AT	CLK2	0.007301747
SMAD6_AP	CLK2	2.36524E-06
SMARCC2_AD	CLK2	0.000380022
SMARCD3_RI	CLK2	1.46538E-18
SMARCE1_AT	CLK2	8.01456E-05

SMC5_ES	CLK2	1.63515E-06
SMG1_AT	CLK2	1.0131E-10
SMIM12_AD	CLK2	0.005392323
SMIM5_AP	CLK2	1.07922E-07
SMN1_RI	CLK2	4.26102E-05
SMN1_AA	CLK2	0.001262069
SMPD4_ES	CLK2	0.00068037
SMTN_ES	CLK2	4.45775E-07
SMUG1_ES	CLK2	2.17351E-06
SMYD5_AA	CLK2	6.81812E-16
SNAPC2_AP	CLK2	7.44664E-06
SNAPC3_AT	CLK2	0.003721979
SNED1_AT	CLK2	0.001544769
SNRPN_AP	CLK2	5.7613E-06
SNTB1_AP	CLK2	1.75308E-05
SNX13_RI	CLK2	0
SNX13_AA	CLK2	0.011789289
SNX19_AP	CLK2	1.09655E-08
SNX19_AD	CLK2	5.88613E-06
SNX20_AT	CLK2	0.000238464
SNX21_AT	CLK2	0
SNX3_AD	CLK2	1.68556E-05
SNX5_AT	CLK2	6.00622E-08
SNX6_AA	CLK2	6.99659E-09
SOD2_AP	CLK2	3.84304E-07
SOD2_AT	CLK2	8.42784E-08
SP140L_AT	CLK2	0.000925518
SPAG1_AT	CLK2	1.4899E-06
SPATA13_AP	CLK2	0.001765395
SPATA20_RI	CLK2	0
SPATA20_AD	CLK2	9.56288E-12
SPATS2_AT	CLK2	0.002038414
SPDL1_AT	CLK2	3.01308E-12
SPG7_AT	CLK2	0.000505074
SPHK2_RI	CLK2	0
SPIN2A_AP	CLK2	3.98395E-06
SPIN2B_AD	CLK2	0.000124231
SPIN3_AT	CLK2	0.000346935
SPINK5_AT	CLK2	8.59735E-09
SPRED2_AP	CLK2	2.1933E-10
SPRY2_AP	CLK2	1.84701E-16
SPSB3_AP	CLK2	5.85516E-09
SPTB_AT	CLK2	0.002027962
SPTLC1_AT	CLK2	0.014728444
SRCAP_ES	CLK2	0.000809184
SREBF1_AP	CLK2	1.13926E-14
SREBF1_RI	CLK2	0
SRI_AP	CLK2	0
SRI_AT	CLK2	7.12978E-11
SRP68_AP	CLK2	0.001773657
SRP9_ES	CLK2	2.08006E-14
SRPK2_AT	CLK2	0.006401456
SRRM1_AD	CLK2	0.006975091

SRRM1_ES	CLK2	3.29295E-18
SRRT_ES	CLK2	2.5084E-16
SRSF1_RI	CLK2	0
SRSF11_AP	CLK2	7.45131E-07
SRSF11_ES	CLK2	8.09969E-07
SRSF11_ES	CLK2	0.006280476
SRSF2_RI	CLK2	0
SRSF2_RI	CLK2	0
SRSF2_RI	CLK2	0
SRSF2_AD	CLK2	2.03927E-10
SRSF5_AT	CLK2	0
SRSF5_AA	CLK2	0
SRSF5_AD	CLK2	3.19986E-07
SRSF5_AD	CLK2	4.78226E-08
SRSF6_ES	CLK2	0
SRSF7_AA	CLK2	5.54323E-15
SRSF7_AD	CLK2	1.18803E-12
SRSF7_ES	CLK2	6.48588E-05
SS18_ES	CLK2	5.28408E-07
SSBP1_AD	CLK2	4.38306E-05
SSBP4_ES	CLK2	0.00019489
SSFA2_AP	CLK2	0.004502729
SSFA2_AD	CLK2	2.53026E-13
SSH1_AT	CLK2	0.007270244
SSH2_AT	CLK2	4.11984E-11
SSH3_RI	CLK2	0
SSH3_AA	CLK2	8.32937E-05
SSR1_AT	CLK2	0.001094563
SSR4_AP	CLK2	9.26813E-06
ST20_AP	CLK2	1.46782E-05
ST20_AP	CLK2	1.02177E-15
ST3GAL4_AP	CLK2	7.81467E-05
ST5_AP	CLK2	7.49827E-09
ST5_ES	CLK2	0.000265389
ST6GALNAC1_AT	CLK2	6.18662E-07
ST6GALNAC1_ES	CLK2	2.22587E-07
ST7_AT	CLK2	0.000279284
ST8SIA1_AT	CLK2	0.000149959
STAG1_AT	CLK2	0.006431527
STAG2_ES	CLK2	1.74478E-06
STAMBP_AP	CLK2	1.19634E-05
STAMBP_AT	CLK2	3.47336E-06
STAP2_AA	CLK2	4.63671E-15
STAP2_AA	CLK2	3.72851E-09
STARD10_AP	CLK2	0.000239291
STARD3_AA	CLK2	7.92313E-13
STAT1_AT	CLK2	0.00090542
STAT2_AA	CLK2	0.014291794
STAT3_AD	CLK2	0.004139379
STAT6_AP	CLK2	6.1898E-07
STEAP4_AT	CLK2	4.01511E-14
STK16_AA	CLK2	0.001255826
STK16_AD	CLK2	7.02871E-06

STK17B_AP	CLK2	3.21477E-05
STK25_AP	CLK2	1.15398E-07
STRA13_AD	CLK2	0.000409292
STRA13_AD	CLK2	0
STRA13_ES	CLK2	7.29925E-13
STRADA_RI	CLK2	4.20046E-13
STRADA_RI	CLK2	2.95937E-14
STRADA_RI	CLK2	3.33207E-06
STRADA_AA	CLK2	1.10646E-06
STRADB_AA	CLK2	1.32257E-09
STRAP_ES	CLK2	0.000742859
STRIP2_AT	CLK2	0.011200024
STRN3_ES	CLK2	0.010214012
STRN4_AA	CLK2	0.000181212
STX10_RI	CLK2	0
STX16_AD	CLK2	0.007333975
STX16_RI	CLK2	0.001255839
STX2_ES	CLK2	1.33321E-06
STXBP2_AP	CLK2	0
STYXL1_AD	CLK2	0.002177493
SUGP1_AA	CLK2	0
SUGP2_AT	CLK2	0.000131055
SUGP2_ES	CLK2	2.86052E-05
SUGP2_AD	CLK2	1.98426E-12
SUGP2_ES	CLK2	2.39474E-09
SULF2_ES	CLK2	0.000855719
SULT1A1_AP	CLK2	0.010803679
SULT1A1_AP	CLK2	0.002445801
SULT1A1_ES	CLK2	8.30377E-13
SULT1A2_AA	CLK2	3.76083E-08
SULT1A2_RI	CLK2	0
SULT1A3_AP	CLK2	0
SULT1A3_AP	CLK2	1.89908E-07
SULT1A3_RI	CLK2	0
SULT1A4_AP	CLK2	0
SUMF1_AT	CLK2	7.20885E-08
SUMF2_ES	CLK2	3.47082E-08
SUMO3_AT	CLK2	6.01868E-07
SUN1_ES	CLK2	4.80312E-10
SUOX_AP	CLK2	0
SUOX_ES	CLK2	0.00067405
SUOX_ES	CLK2	0.001196381
SUPT20H_ES	CLK2	0.000246461
SUPT4H1_AP	CLK2	9.18794E-15
SUPT4H1_AD	CLK2	1.12154E-15
SUPT4H1_AD	CLK2	5.48827E-14
SUPT5H_ES	CLK2	3.98554E-07
SUPT7L_RI	CLK2	4.14429E-12
SUPT7L_RI	CLK2	0
SV2A_AT	CLK2	1.19594E-06
SVEP1_AT	CLK2	0.000211698
SYK_ES	CLK2	0.005048053
SYMPK_AP	CLK2	3.27701E-11

SYNE2_AP	CLK2	3.44403E-06
SYNE2_AA	CLK2	3.85239E-06
SYNGR2_AA	CLK2	6.06102E-15
SYNGR2_AA	CLK2	0
SYNJ2_AP	CLK2	0.002029467
SYNJ2_AT	CLK2	2.49138E-06
SYNM_RI	CLK2	5.77738E-07
SYNPO_AT	CLK2	0.011363541
SYP_AT	CLK2	2.75901E-09
SYS1_AT	CLK2	4.95377E-08
SYT15_AA	CLK2	1.29406E-13
SYT17_AP	CLK2	3.184E-05
SYTL1_AP	CLK2	0
SYTL2_AP	CLK2	0.000181701
SYTL2_ES	CLK2	5.86681E-15
SYTL2_ES	CLK2	0.000848356
SYTL4_AT	CLK2	5.01949E-07
SZT2_AT	CLK2	0.002676911
TAB3_AT	CLK2	7.83692E-14
TAB3_RI	CLK2	2.74535E-11
TADA2A_AD	CLK2	0.002324422
TADA2B_AP	CLK2	2.64831E-16
TADA3_AT	CLK2	1.7766E-09
TAF12_AD	CLK2	3.5349E-14
TAF1C_RI	CLK2	2.82727E-07
TAF1D_AT	CLK2	0.000379696
TAF1D_RI	CLK2	7.27461E-12
TAF1D_RI	CLK2	8.29297E-08
TAF1D_AA	CLK2	0.007988554
TAF6_AP	CLK2	0
TAF6_AD	CLK2	1.08057E-06
TAF6_AD	CLK2	1.25079E-06
TAF9_AP	CLK2	8.1849E-14
TAGLN_RI	CLK2	5.23003E-05
TANK_AP	CLK2	0.005821054
TANK_AT	CLK2	0.004013002
TANK_ES	CLK2	8.10341E-06
TANK_ES	CLK2	4.05043E-05
TAOK2_AP	CLK2	0
TAOK3_AP	CLK2	0
TARBP2_AA	CLK2	3.5631E-07
TATDN1_AT	CLK2	0.010421752
TATDN1_ES	CLK2	0.014710226
TAZ_RI	CLK2	0
TAZ_AD	CLK2	0
TBC1D1_AT	CLK2	0.005308723
TBC1D10A_AP	CLK2	1.63593E-06
TBC1D10A_ES	CLK2	6.64835E-08
TBC1D14_AP	CLK2	0.002186946
TBC1D17_ES	CLK2	0
TBC1D3_AD	CLK2	5.4888E-09
TBC1D7_AT	CLK2	2.26024E-05
TBC1D7_AD	CLK2	0.000596888

TBC1D7_AD	CLK2	0.007467433
TBC1D8B_AT	CLK2	0
TBC1D9B_AP	CLK2	4.39203E-12
TBCD_AT	CLK2	0.000328279
TBCE_ES	CLK2	1.00394E-05
TBL2_AT	CLK2	1.07677E-06
TBL2_AD	CLK2	0.000590789
TBL2_AA	CLK2	1.96439E-11
TC2N_AD	CLK2	0.002201849
TCAIM_AP	CLK2	0.002409724
TCEAL4_AA	CLK2	2.45171E-13
TCEAL8_ES	CLK2	0.00131273
TCEB1_AP	CLK2	0.006597613
TCEB1_AT	CLK2	0.003662942
TCEB1_ES	CLK2	0.008974367
TCF12_AP	CLK2	0.003363627
TCF12_ES	CLK2	0.00036645
TCF25_ES	CLK2	5.28173E-13
TCF3_AP	CLK2	1.93057E-16
TCF7_AT	CLK2	1.37599E-06
TCFL5_AT	CLK2	0.009387294
TCHP_AT	CLK2	0.006087038
TCIRG1_RI	CLK2	0
TCOF1_AT	CLK2	1.18E-08
TCTN1_AT	CLK2	0.013056688
TDP2_AD	CLK2	0
TEAD4_AP	CLK2	0.008299716
TECPR2_AT	CLK2	0.004289622
TECR_AA	CLK2	0.001208838
TEFM_RI	CLK2	0
TES_AP	CLK2	1.30005E-09
TET2_AT	CLK2	3.57764E-06
TET2_RI	CLK2	2.6083E-05
TEX10_AT	CLK2	0.001625085
TEX264_ES	CLK2	7.84815E-05
TEX264_ES	CLK2	0.000377501
TEX264_AD	CLK2	5.68598E-12
TEX30_AP	CLK2	4.84256E-15
TFDP1_ES	CLK2	6.80829E-07
TFDP1_ES	CLK2	1.94152E-08
TFG_AD	CLK2	0.000100127
TGIF1_AP	CLK2	0
TGIF1_AD	CLK2	2.744E-09
TGIF1_AD	CLK2	0.000325853
THAP2_AT	CLK2	0.000447079
THAP4_AP	CLK2	6.53639E-07
THAP5_AP	CLK2	0.012107137
THAP6_AT	CLK2	5.33796E-10
THAP7_RI	CLK2	0.00243428
THNSL2_AP	CLK2	0.003503707
THOC5_ES	CLK2	0.001013644
THOP1_AP	CLK2	0
THRA_AT	CLK2	4.3942E-05

THTPA_AD	CLK2	1.19063E-05
THTPA_RI	CLK2	0.011827375
THYN1_RI	CLK2	0.015333988
TIAL1_AT	CLK2	8.87227E-17
TIAL1_ES	CLK2	2.48255E-16
TIMM10_AA	CLK2	0.001183005
TIMM17B_AA	CLK2	1.36643E-08
TIMM8B_ES	CLK2	3.44502E-09
TINAGL1_RI	CLK2	0
TK1_RI	CLK2	9.13488E-05
TLE1_AT	CLK2	1.03582E-11
TLE2_RI	CLK2	0
TLE2_RI	CLK2	0
TLK1_ES	CLK2	4.0671E-06
TM2D3_RI	CLK2	1.10591E-16
TM2D3_AD	CLK2	1.43365E-13
TM9SF1_AT	CLK2	1.53169E-07
TMC4_AA	CLK2	0.004382516
TMC5_AP	CLK2	0.000750816
TMC6_AP	CLK2	0
TMC6_AD	CLK2	0
TMC6_ES	CLK2	2.39518E-09
TMC6_RI	CLK2	2.37687E-06
TMCC1_AP	CLK2	7.31507E-06
TMCO1_AP	CLK2	0.000249272
TMCO4_ES	CLK2	0.001026801
TMCO6_RI	CLK2	0
TMCO6_RI	CLK2	0
TMEM107_RI	CLK2	2.41398E-09
TMEM126B_RI	CLK2	0
TMEM128_AD	CLK2	3.0861E-12
TMEM134_ES	CLK2	5.2249E-10
TMEM134_AA	CLK2	2.92977E-14
TMEM134_ES	CLK2	5.8747E-07
TMEM134_AA	CLK2	0.000924879
TMEM134_AD	CLK2	0.001805473
TMEM134_AD	CLK2	0.000110664
TMEM135_AT	CLK2	1.20467E-05
TMEM138_AT	CLK2	0
TMEM138_RI	CLK2	0
TMEM139_RI	CLK2	0.000374566
TMEM150A_ES	CLK2	5.47221E-15
TMEM150A_AD	CLK2	2.94546E-18
TMEM159_ES	CLK2	6.0468E-06
TMEM161B_AT	CLK2	9.54212E-07
TMEM161B_AD	CLK2	4.67106E-20
TMEM161B_RI	CLK2	0
TMEM161B_RI	CLK2	0
TMEM168_ES	CLK2	1.4658E-06
TMEM175_AA	CLK2	8.72527E-08
TMEM175_ES	CLK2	8.59108E-19
TMEM175_ES	CLK2	1.87438E-16
TMEM177_AT	CLK2	3.84083E-05

TMEM184A_AP	CLK2	1.48076E-13
TMEM185A_AT	CLK2	0.001488299
TMEM2_AP	CLK2	8.3314E-05
TMEM205_RI	CLK2	3.46375E-12
TMEM205_RI	CLK2	1.06168E-17
TMEM205_RI	CLK2	0
TMEM205_RI	CLK2	0
TMEM205_ES	CLK2	0.000936547
TMEM205_ES	CLK2	0.000992231
TMEM205_AD	CLK2	2.86256E-05
TMEM205_AD	CLK2	0.013780801
TMEM219_AP	CLK2	7.00787E-10
TMEM223_AT	CLK2	0
TMEM230_AD	CLK2	0.004363167
TMEM230_AD	CLK2	2.11109E-05
TMEM230_AD	CLK2	2.5146E-05
TMEM234_RI	CLK2	0.000125383
TMEM234_RI	CLK2	1.95707E-19
TMEM234_ES	CLK2	8.0739E-14
TMEM242_AT	CLK2	0.003203523
TMEM243_AT	CLK2	1.353E-06
TMEM251_AD	CLK2	0.001220319
TMEM33_RI	CLK2	0.002145505
TMEM39B_AT	CLK2	1.99501E-10
TMEM43_ES	CLK2	0.012770753
TMEM44_ES	CLK2	0
TMEM55B_RI	CLK2	0
TMEM55B_AD	CLK2	0.001003845
TMEM59_AP	CLK2	1.66993E-06
TMEM59_AP	CLK2	2.35325E-14
TMEM63A_AP	CLK2	0
TMEM63A_ES	CLK2	0
TMEM67_AT	CLK2	0.024981903
TMEM68_AT	CLK2	0.00014939
TMEM87A_AP	CLK2	0.000431014
TMEM88_AA	CLK2	1.87102E-06
TMEM8B_AP	CLK2	0.002505028
TMEM8B_AT	CLK2	0.002269043
TMEM91_AP	CLK2	0.000747386
TMEM91_RI	CLK2	9.84761E-08
TMEM91_RI	CLK2	0.000395297
TMEM91_RI	CLK2	0.000192527
TMEM98_AT	CLK2	9.0404E-06
TMEM9B_AP	CLK2	0.000136351
TMPRSS2_AP	CLK2	0.000148572
TMSB4X_RI	CLK2	6.98375E-06
TMSB4X_RI	CLK2	4.49907E-11
TMTC4_ES	CLK2	0.001498113
TMUB1_AP	CLK2	0.000557572
TMUB2_AP	CLK2	0.004307866
TMUB2_AT	CLK2	6.60142E-13
TMUB2_ES	CLK2	0.010986335
TMUB2_AA	CLK2	0.000496846

TMUB2_ES	CLK2	0.000438553
TMUB2_ES	CLK2	7.37721E-08
TMUB2_AD	CLK2	0.005319984
TMUB2_AD	CLK2	0.000606769
TMX2_AA	CLK2	4.66793E-06
TNC_ES	CLK2	0.000142687
TNC_ES	CLK2	0.001951567
TNC_ES	CLK2	0.013040864
TNC_ES	CLK2	0.006132991
TNFAIP8_AP	CLK2	2.15077E-08
TNFRSF10B_RI	CLK2	3.22901E-07
TNFRSF10C_AT	CLK2	2.32562E-05
TNFRSF14_AA	CLK2	0
TNFRSF1A_AT	CLK2	1.3433E-12
TNFRSF25_RI	CLK2	7.79344E-12
TNFRSF25_AA	CLK2	0.00019081
TNFSF15_AP	CLK2	1.64406E-17
TNIK_AT	CLK2	0.003142228
TNIP1_AA	CLK2	7.05882E-07
TNK2_ES	CLK2	4.60744E-06
TNK2_AA	CLK2	8.06047E-11
TNK2_ES	CLK2	1.21182E-11
TOM1L2_ES	CLK2	0
TOP3B_RI	CLK2	0
TOP3B_RI	CLK2	0
TOP3B_AD	CLK2	0.006942111
TOP3B_RI	CLK2	0.00308824
TOP3B_ES	CLK2	0
TOPORS_ES	CLK2	4.19831E-06
TOR2A_RI	CLK2	2.49899E-14
TOR2A_RI	CLK2	0
TP53_AT	CLK2	0.006323016
TP53I3_ES	CLK2	4.65564E-05
TP53I3_RI	CLK2	0
TPD52_AP	CLK2	3.25206E-12
TPD52L1_ES	CLK2	0.006841804
TPGS2_AT	CLK2	0.000715606
TPM1_AD	CLK2	0.003086446
TPM1_ES	CLK2	1.7642E-10
TPM1_AA	CLK2	7.27329E-17
TPPP3_AD	CLK2	5.66666E-08
TPRA1_AP	CLK2	0.007892026
TPT1_RI	CLK2	0.006544489
TPT1_AD	CLK2	8.13302E-12
TRA2A_ES	CLK2	7.75934E-19
TRA2B_ES	CLK2	0.011981121
TRABD_AP	CLK2	0.001413781
TRABD_RI	CLK2	8.75897E-06
TRABD2A_AT	CLK2	0.008135729
TRADD_AP	CLK2	0
TRAF1_AP	CLK2	2.44692E-05
TRAF4_AT	CLK2	0
TRAFD1_ES	CLK2	7.54962E-05

TRAFD1_AD	CLK2	0.000863964
TRAK1_AT	CLK2	8.34132E-12
TRAM1_RI	CLK2	0.01292491
TRAP1_AP	CLK2	0
TRAPPC1_RI	CLK2	7.71368E-05
TRAPPC2_RI	CLK2	3.23752E-05
TRAPPC2_ES	CLK2	1.51866E-07
TRAPPC4_AT	CLK2	0.000373647
TRDMT1_AT	CLK2	0.00028295
TREX1_RI	CLK2	1.42698E-05
TREX1_RI	CLK2	0
TREX1_RI	CLK2	9.43172E-08
TRIM11_AP	CLK2	2.39151E-10
TRIM11_AT	CLK2	0
TRIM13_RI	CLK2	2.97514E-10
TRIM13_RI	CLK2	4.3507E-18
TRIM13_AD	CLK2	0.006329823
TRIM14_AT	CLK2	1.15098E-09
TRIM24_AD	CLK2	0.0017183
TRIM36_AT	CLK2	9.83653E-05
TRIM4_AT	CLK2	0.000244222
TRIM41_RI	CLK2	2.72439E-05
TRIM5_RI	CLK2	0.002318309
TRIM5_RI	CLK2	4.83001E-12
TRIM7_AT	CLK2	0.012492568
TRIM73_AP	CLK2	0.012908119
TRIM73_AT	CLK2	1.91664E-11
TRIO_AT	CLK2	2.1633E-08
TRIT1_AT	CLK2	0.000275938
TRMT13_AT	CLK2	3.93885E-13
TRMT2A_AA	CLK2	3.81728E-09
TRMT2B_AT	CLK2	0.003018725
TRMT2B_AD	CLK2	2.73335E-05
TRMT44_AT	CLK2	0.000708416
TRMU_AT	CLK2	0
TRMU_ES	CLK2	0.002436565
TRNT1_AT	CLK2	0.003029062
TRNT1_AD	CLK2	4.54626E-07
TROAP_AT	CLK2	1.83305E-10
TROAP_AT	CLK2	2.15144E-06
TROAP_RI	CLK2	0
TRPM6_AT	CLK2	2.5646E-07
TRPT1_ES	CLK2	0.00331871
TSC1_AT	CLK2	0.000165703
TSC2_ES	CLK2	1.60903E-12
TSC2_ES	CLK2	5.27096E-08
TSC22D1_AP	CLK2	0
TSEN2_AT	CLK2	4.31922E-06
TSG101_AT	CLK2	0.000380007
TSPAN17_RI	CLK2	1.34465E-06
TSPAN17_RI	CLK2	1.51762E-08
TSPAN17_AD	CLK2	0.008867068
TSPAN17_RI	CLK2	0

TSPAN31_AT	CLK2	2.13716E-11
TSPAN8_AP	CLK2	9.60556E-05
TSSC4_ES	CLK2	4.87333E-07
TST_RI	CLK2	0.000107682
TTC13_ME	CLK2	0.001265805
TTC14_AT	CLK2	0.005401809
TTC14_RI	CLK2	0
TTC14_RI	CLK2	0
TTC17_AT	CLK2	0.000282073
TTC31_RI	CLK2	0
TTC31_RI	CLK2	0
TTC31_RI	CLK2	0
TTC31_RI	CLK2	0
TTC39C_AP	CLK2	0.000224808
TTC39C_AP	CLK2	1.90451E-10
TTC7A_AP	CLK2	0.00281407
TTC9C_AT	CLK2	2.22517E-11
TTC9C_ES	CLK2	1.17714E-05
TTI1_ES	CLK2	0.008650769
TTI2_RI	CLK2	0.004580797
TTLL11_AT	CLK2	0.000940491
TTLL12_AP	CLK2	1.49965E-06
TTLL3_AT	CLK2	0.007132137
TTLL3_RI	CLK2	2.27163E-07
TTLL5_AT	CLK2	9.76853E-09
TTYH3_AP	CLK2	8.43794E-12
TUBA4A_AP	CLK2	4.47331E-10
TUBB3_AP	CLK2	4.5412E-05
TUBB3_AP	CLK2	1.89201E-06
TUBGCP3_AT	CLK2	4.25974E-05
TUBGCP5_AT	CLK2	0.000168972
TVP23A_AT	CLK2	4.84075E-06
TVP23B_AP	CLK2	1.11332E-09
TVP23C_AT	CLK2	3.36408E-05
TWF1_AD	CLK2	4.74545E-09
TWSG1_AT	CLK2	1.41469E-07
TXN2_AP	CLK2	2.64888E-10
TXNDC17_AD	CLK2	1.24268E-05
TXNDC9_AT	CLK2	1.79828E-16
TXNRD2_RI	CLK2	0
TYROBP_AA	CLK2	5.30341E-07
TYSND1_ES	CLK2	0.014012144
U2AF1L4_AA	CLK2	0.000177487
U2AF2_AD	CLK2	0.011037872
UAP1_ES	CLK2	0.00128986
UAP1_ES	CLK2	0.009490856
UBA1_AP	CLK2	1.56132E-17
UBA52_AP	CLK2	9.53508E-05
UBA52_AD	CLK2	0.001929689
UBALD1_AP	CLK2	1.18802E-13
UBALD1_RI	CLK2	4.03263E-07
UBALD1_RI	CLK2	0
UBAP2_AP	CLK2	2.76485E-12

UBAP2L_AD	CLK2	0.00359365
UBE2D3_AP	CLK2	2.63711E-14
UBE2D3_AD	CLK2	5.17559E-09
UBE2D3_AD	CLK2	1.19696E-05
UBE2G2_ES	CLK2	0
UBE2J2_ES	CLK2	0.000110137
UBE2N_AD	CLK2	1.75658E-05
UBE2V1_ES	CLK2	0.000521247
UBE2Z_AP	CLK2	0
UBE3B_AT	CLK2	2.23797E-07
UBE3B_AA	CLK2	0.009872115
UBFD1_AT	CLK2	0.000918246
UBL7_AD	CLK2	0.000662875
UBL7_AD	CLK2	9.90836E-06
UBP1_ES	CLK2	4.4068E-20
UBQLN1_ES	CLK2	9.03217E-05
UBR7_ES	CLK2	1.50875E-10
UBTF_AP	CLK2	0.005607786
UBXN11_AT	CLK2	0
UBXN4_RI	CLK2	4.1129E-17
UCHL5_AP	CLK2	7.6662E-06
UCHL5_AT	CLK2	5.96279E-08
UCHL5_ES	CLK2	9.78237E-09
UCK1_AA	CLK2	8.81388E-06
UCKL1_AP	CLK2	0
UCKL1_RI	CLK2	1.02276E-17
UEVLD_AT	CLK2	3.51615E-14
UGDH_AP	CLK2	0.000568645
UGGT2_AT	CLK2	3.11232E-09
UGP2_AP	CLK2	5.93594E-15
UGT1A1_AT	CLK2	0.000198252
UGT1A10_AT	CLK2	1.00067E-06
ULK1_AP	CLK2	0
ULK3_RI	CLK2	0
UMPS_ES	CLK2	3.59198E-05
UNC119_AP	CLK2	0
UNC119_RI	CLK2	1.2677E-17
UNC93B1_AA	CLK2	4.71756E-13
UNG_AP	CLK2	2.23457E-08
UNK_AT	CLK2	0
UNKL_AT	CLK2	0.000806647
UPF1_AD	CLK2	0.000672198
UPF3A_ES	CLK2	0.013811341
UPK3B_AT	CLK2	1.11216E-13
UPP1_AT	CLK2	2.18873E-09
UPP1_AA	CLK2	0.006776176
UQCC1_AT	CLK2	1.46E-18
UQCR10_AD	CLK2	3.29214E-06
UQCR11_AT	CLK2	8.82126E-13
UQCRQ_RI	CLK2	1.75106E-10
URGCP_RI	CLK2	6.00395E-16
USE1_AT	CLK2	4.40516E-09
USF2_RI	CLK2	0.000205252

USMG5_ES	CLK2	0.001058366
USP1_AP	CLK2	0.012865425
USP19_AT	CLK2	5.60316E-10
USP21_ES	CLK2	3.49259E-12
USP3_ES	CLK2	5.35255E-05
USP33_ES	CLK2	1.09014E-06
USP35_AP	CLK2	4.77503E-16
USP36_RI	CLK2	0
USP4_AT	CLK2	1.7097E-15
USP4_AT	CLK2	1.4257E-16
USP4_ES	CLK2	0.002756145
USP45_AT	CLK2	3.26151E-05
USP48_AP	CLK2	3.11175E-06
USP48_AT	CLK2	8.19135E-06
USP49_AT	CLK2	6.01731E-05
USP54_ES	CLK2	0.000593923
USP6NL_AT	CLK2	3.44506E-15
UVRAG_AP	CLK2	3.30307E-05
VAMP1_RI	CLK2	5.34787E-11
VAMP1_RI	CLK2	0
VAMP1_RI	CLK2	0.000151127
VAMP2_RI	CLK2	0
VAMP3_AP	CLK2	0
VEGFA_ES	CLK2	9.90674E-07
VEGFB_AA	CLK2	0.001972326
VEZT_ES	CLK2	4.34357E-07
VEZT_ES	CLK2	0.008134884
VIL1_AT	CLK2	0
VKORC1_ES	CLK2	1.17501E-07
VPS13C_AT	CLK2	0.00010646
VPS16_AP	CLK2	2.17491E-16
VPS16_RI	CLK2	0
VPS28_AP	CLK2	0.000182058
VPS28_RI	CLK2	1.01978E-10
VPS28_RI	CLK2	0
VPS28_RI	CLK2	0
VPS28_RI	CLK2	3.71075E-07
VPS33A_AT	CLK2	9.3649E-05
VPS53_AT	CLK2	0.000162912
VPS8_AT	CLK2	0.001144366
VPS9D1_AP	CLK2	4.14656E-10
VTI1A_AT	CLK2	1.86578E-06
VWA2_AT	CLK2	0.000376701
WAC_ES	CLK2	0
WARS2_AA	CLK2	0.000168916
WASH4P_RI	CLK2	0
WDPCP_AT	CLK2	3.21379E-07
WDR11_RI	CLK2	0
WDR13_RI	CLK2	3.8997E-06
WDR20_AT	CLK2	0.016264638
WDR24_ES	CLK2	2.04442E-05
WDR33_AT	CLK2	3.51668E-09
WDR53_ES	CLK2	1.60737E-05

WDR55_AT	CLK2	0.000108912
WDR55_RI	CLK2	1.85309E-20
WDR55_RI	CLK2	0
WDR6_RI	CLK2	0
WDR6_AA	CLK2	1.09021E-07
WDR75_AA	CLK2	0
WDR92_AT	CLK2	5.39953E-06
WHSC1_ES	CLK2	0.000165954
WHSC1L1_AT	CLK2	9.93037E-11
WIBG_AT	CLK2	0.00365632
WIPF1_AT	CLK2	0.000217512
WLS_AT	CLK2	3.18304E-10
WNK2_AT	CLK2	0.001605959
WNT2B_AP	CLK2	1.47127E-10
WRAP53_AP	CLK2	0.000416687
WRAP73_RI	CLK2	2.0051E-10
WRNIP1_AA	CLK2	0.002196122
WSB1_AT	CLK2	1.7012E-06
WSB1_ES	CLK2	1.23676E-07
WSB2_AP	CLK2	0.001872953
WWC1_AA	CLK2	0.001978916
WWP2_AT	CLK2	0.002166779
XAF1_AA	CLK2	4.30252E-15
XPA_ES	CLK2	0
XPNPEP3_AT	CLK2	1.10856E-06
XPO1_AP	CLK2	1.62966E-12
XRCC3_AP	CLK2	0.008668761
XRCC3_AT	CLK2	0.012435488
XRRA1_AT	CLK2	0.004805534
XRRA1_RI	CLK2	6.92041E-06
YAF2_AT	CLK2	1.92883E-07
YBEY_AD	CLK2	0.01318079
YBX3_ES	CLK2	3.49618E-11
YDJC_AD	CLK2	0.001565671
YIF1B_AP	CLK2	1.75021E-07
YIF1B_AT	CLK2	0.000656141
YIPF2_AD	CLK2	0.003465856
YME1L1_RI	CLK2	0.006916959
YME1L1_AA	CLK2	5.41063E-09
YPEL3_AT	CLK2	3.83894E-14
YPEL3_AD	CLK2	0
YPEL3_RI	CLK2	2.14035E-16
YPEL5_ES	CLK2	1.96415E-09
YTHDC1_ES	CLK2	0.004715695
YWHAE_ES	CLK2	0.006598649
YWHAQ_AP	CLK2	0.002323215
YWHAZ_ES	CLK2	6.29245E-05
YY1AP1_AA	CLK2	8.55461E-11
ZADH2_AP	CLK2	2.74659E-05
ZBED1_AP	CLK2	0.006267314
ZBED5_AA	CLK2	0.00076298
ZBP1_AT	CLK2	9.50228E-06
ZBTB17_AA	CLK2	0.000263631

ZBTB17_RI	CLK2	2.07635E-17
ZBTB45_AD	CLK2	9.63672E-06
ZBTB7B_AP	CLK2	4.81416E-10
ZBTB8OS_AT	CLK2	0.005156529
ZC3H14_ES	CLK2	0.00231903
ZC3H14_ES	CLK2	0.008708361
ZC3H18_ES	CLK2	0.006684186
ZC3H7A_AP	CLK2	1.80286E-14
ZCCHC10_AT	CLK2	0.002580329
ZCCHC4_AT	CLK2	0.000246776
ZCCHC8_AP	CLK2	2.05221E-07
ZCCHC8_RI	CLK2	2.158E-14
ZDHHC16_ME	CLK2	6.70796E-07
ZDHHC20_ES	CLK2	0.000521498
ZDHHC4_AA	CLK2	0.000228418
ZDHHC4_ES	CLK2	0.001943301
ZDHHC4_ES	CLK2	2.8828E-05
ZDHHC4_AA	CLK2	0.001230137
ZDHHC4_ES	CLK2	0.009971912
ZDHHC9_AP	CLK2	4.48624E-07
ZEB2_AT	CLK2	0.000903463
ZFAND2B_RI	CLK2	0
ZFAND2B_RI	CLK2	0
ZFAND2B_RI	CLK2	0.000775425
ZFAND2B_RI	CLK2	0.007728346
ZFAND4_AT	CLK2	0.000108703
ZFAND5_AP	CLK2	0.002976996
ZFP36_AD	CLK2	0
ZFP41_AT	CLK2	3.08156E-07
ZFYVE26_AT	CLK2	4.60565E-05
ZFYVE27_AA	CLK2	0.014174381
ZFYVE27_ES	CLK2	2.55775E-07
ZFYVE28_AT	CLK2	0.000288987
ZMIZ1_ES	CLK2	0.005183171
ZMYM3_AT	CLK2	2.70708E-05
ZMYM5_AT	CLK2	0.001920873
ZMYND11_AP	CLK2	0.007683632
ZMYND11_AP	CLK2	3.0423E-06
ZMYND8_AD	CLK2	0.000138979
ZNF124_AT	CLK2	2.97417E-05
ZNF141_AT	CLK2	1.07528E-12
ZNF154_RI	CLK2	1.99724E-11
ZNF160_AP	CLK2	0
ZNF169_AT	CLK2	0.00224908
ZNF19_AT	CLK2	0.000990998
ZNF195_ES	CLK2	1.70092E-05
ZNF195_ES	CLK2	4.35228E-06
ZNF197_AT	CLK2	5.52307E-05
ZNF20_AT	CLK2	5.99925E-05
ZNF200_AD	CLK2	1.66665E-09
ZNF207_ES	CLK2	0.000458711
ZNF213_AA	CLK2	7.25557E-09
ZNF223_AT	CLK2	0.000717998

ZNF226_RI	CLK2	0
ZNF23_AD	CLK2	0.000591127
ZNF233_AT	CLK2	3.41641E-08
ZNF24_AA	CLK2	8.30392E-11
ZNF248_AT	CLK2	0.0035811
ZNF263_AT	CLK2	0.000670871
ZNF263_ES	CLK2	0.001189407
ZNF264_AT	CLK2	1.25414E-06
ZNF266_AP	CLK2	4.16381E-13
ZNF276_AP	CLK2	3.24556E-07
ZNF276_RI	CLK2	0.000167935
ZNF277_AT	CLK2	2.2558E-05
ZNF280D_AT	CLK2	0.000832479
ZNF280D_AT	CLK2	1.27552E-09
ZNF283_AT	CLK2	0.015544794
ZNF292_AT	CLK2	0.003587633
ZNF3_AP	CLK2	2.44071E-15
ZNF317_AA	CLK2	1.86992E-13
ZNF326_AT	CLK2	4.70678E-08
ZNF330_AT	CLK2	6.55453E-11
ZNF333_AT	CLK2	1.84931E-06
ZNF333_AT	CLK2	2.02991E-15
ZNF337_AP	CLK2	7.73258E-12
ZNF33A_AT	CLK2	0.002019619
ZNF347_AD	CLK2	5.26104E-05
ZNF384_AP	CLK2	0.000201367
ZNF384_AD	CLK2	0.007028209
ZNF396_AT	CLK2	0.000515283
ZNF397_AT	CLK2	4.36329E-06
ZNF410_AP	CLK2	0.001681594
ZNF419_AT	CLK2	8.75772E-07
ZNF429_AT	CLK2	0.000228421
ZNF430_AT	CLK2	8.51478E-06
ZNF431_AT	CLK2	2.03352E-05
ZNF44_AT	CLK2	2.72432E-07
ZNF44_AT	CLK2	0.013750379
ZNF468_ES	CLK2	0.013092761
ZNF493_AT	CLK2	4.7107E-07
ZNF506_ES	CLK2	3.55414E-06
ZNF506_ES	CLK2	6.34454E-05
ZNF511_AT	CLK2	0.000279851
ZNF511_RI	CLK2	0
ZNF512_AT	CLK2	0.000910701
ZNF519_AT	CLK2	0.008316568
ZNF519_AT	CLK2	0.000647857
ZNF524_AP	CLK2	1.97986E-06
ZNF525_AT	CLK2	0.000775003
ZNF544_AT	CLK2	1.91364E-05
ZNF548_AT	CLK2	0.007741661
ZNF549_AT	CLK2	4.95901E-06
ZNF550_ES	CLK2	0.002160415
ZNF558_AP	CLK2	7.79735E-15
ZNF561_AA	CLK2	2.76388E-06

ZNF568_AT	CLK2	3.49983E-05
ZNF577_RI	CLK2	0.000105084
ZNF580_AP	CLK2	5.66702E-05
ZNF581_AP	CLK2	6.34913E-06
ZNF585A_AT	CLK2	2.92536E-15
ZNF585A_AT	CLK2	0.000128251
ZNF585B_AP	CLK2	3.10085E-05
ZNF586_AT	CLK2	8.87518E-05
ZNF587_AP	CLK2	0.000295072
ZNF596_AD	CLK2	2.61314E-15
ZNF606_AT	CLK2	5.89518E-08
ZNF616_AT	CLK2	0.000109659
ZNF646_RI	CLK2	0.000132693
ZNF655_AP	CLK2	0.004684469
ZNF655_AT	CLK2	4.37759E-15
ZNF66_AT	CLK2	0.014207014
ZNF670_AT	CLK2	1.11922E-10
ZNF675_AT	CLK2	4.73348E-09
ZNF684_AT	CLK2	2.80155E-07
ZNF687_ES	CLK2	0.000256318
ZNF69_AT	CLK2	3.9052E-07
ZNF692_RI	CLK2	9.92643E-05
ZNF692_RI	CLK2	4.10437E-13
ZNF692_AA	CLK2	0.007184911
ZNF696_AT	CLK2	1.47834E-09
ZNF7_AT	CLK2	8.06793E-06
ZNF700_AP	CLK2	5.68345E-18
ZNF706_AP	CLK2	2.88041E-18
ZNF706_AD	CLK2	2.15236E-12
ZNF706_RI	CLK2	1.02438E-11
ZNF706_AA	CLK2	8.42906E-06
ZNF714_AT	CLK2	0.015393388
ZNF717_AP	CLK2	6.40074E-05
ZNF717_AT	CLK2	1.29275E-10
ZNF738_AT	CLK2	4.23454E-11
ZNF76_RI	CLK2	0
ZNF76_RI	CLK2	0
ZNF76_AA	CLK2	0
ZNF76_AA	CLK2	2.0221E-16
ZNF771_AT	CLK2	6.79836E-07
ZNF771_AT	CLK2	0.001029206
ZNF774_AT	CLK2	5.71154E-08
ZNF783_AT	CLK2	2.15505E-08
ZNF799_AP	CLK2	3.87789E-07
ZNF808_AT	CLK2	0.015616958
ZNF814_AT	CLK2	2.19634E-05
ZNF814_AT	CLK2	2.57017E-08
ZNF814_AT	CLK2	3.39987E-14
ZNF816_AT	CLK2	0.000232319
ZNF83_AP	CLK2	0
ZNF91_AT	CLK2	4.68473E-13
ZNFX1_AT	CLK2	0.016553987
ZNHIT3_AT	CLK2	0

ZNRF1_AT	CLK2	4.25845E-11
ZP3_AP	CLK2	1.62244E-06
ZRSR2_AT	CLK2	0.011670817
ZSCAN2_AT	CLK2	0.013757349
ZSWIM7_RI	CLK2	7.61331E-07
ZSWIM7_ES	CLK2	1.33489E-07
ZSWIM7_ES	CLK2	9.43485E-10
ZWINT_RI	CLK2	0
ZYX_AP	CLK2	1.40273E-06

:ween CLK2 expression and PSI value of differential splicing genes

Correlation
0.369970524
0.267851672
0.33244672
-0.171443096
0.110590417
0.326934928
0.321053074
0.346340729
0.162525744
-0.160561388
0.42282689
0.352629423
0.155486749
0.344347809
0.274883994
0.311169307
0.135332618
0.173687971
0.166144093
0.513855893
0.43521022
0.1197667
0.136370316
0.150544521
-0.31945589
0.126767248
0.443791605
0.438405206
0.240237037
0.162682999
0.459123278
0.365252716
0.47900625
0.556170469
0.140186109
0.300490987
0.128938844
0.202020937
0.188730843
0.105468892
0.165434067
0.11295339
0.20515388
0.157525662
0.145567396
0.189081737
0.296730986
-0.180751197
0.29114418
0.365344272

0.385111564
0.295102678
0.156300256
0.288538417
-0.199886512
0.150069165
0.212941026
0.13416489
0.216014839
0.244702873
0.393474702
0.207184394
-0.208721643
0.444402425
0.344083404
0.365801094
0.401206431
0.431180298
0.109992334
0.168683134
-0.143432113
0.127891118
0.149165744
-0.192483064
0.295420904
0.119460819
0.327667389
0.175049815
0.454378266
0.410561563
0.335976789
0.218167947
0.152170915
0.120591066
0.211468382
0.334235097
0.315292725
0.237686276
0.300824004
0.12622232
0.154101671
-0.168805054
0.157701703
0.212140645
0.179072204
0.108865662
0.197728068
0.128286775
0.338685916
-0.103766158
0.134226987
0.286883846
-0.264308222

-0.156134233
0.118336972
-0.153704756
0.105868786
-0.218455571
-0.180960966
0.213848378
0.337278311
0.435600266
0.318353298
0.182206124
-0.126672666
0.594906868
0.155171464
0.357862423
0.334758989
0.122792168
0.429404297
0.231122396
0.110715332
0.119912955
0.263701124
0.382023422
0.508198915
0.184570923
0.233499488
0.233312545
0.497659901
0.485601241
0.393405352
0.416986323
0.391091208
0.153616442
0.126606049
0.145688726
0.102408351
0.115098042
0.101281279
0.140081245
-0.201406482
0.166518696
0.272607764
-0.135167923
0.105020423
-0.129262792
0.113462924
0.343347441
0.155014001
0.163989372
0.264257342
0.269335378
0.216362192
0.287615459

0.18349301
0.106378764
0.168011502
0.392299741
0.152037485
0.340540304
0.329730764
0.157088945
0.291712362
0.324180484
0.127715492
0.237622017
0.108828918
-0.131825568
0.181686382
0.192937381
0.235005013
0.297835525
0.164803465
0.267260234
0.241877557
0.171965022
-0.106824474
0.388327909
-0.172560552
0.136895763
0.332419406
0.170983262
0.168837046
-0.227634894
0.288067231
0.103793608
0.101653257
0.124978622
-0.123677494
0.122743952
0.114095121
0.183715604
0.318996086
0.128056673
0.246385411
0.172210904
0.179195487
0.460980257
0.181024123
0.108543075
-0.121973692
0.513064607
0.173675619
0.180833888
0.477444955
0.303828289
0.324902019

0.126128225
0.123921286
0.271641486
0.142201047
0.201746375
0.134189503
0.336226815
0.272879699
0.296789902
0.227801353
0.39854863
0.195075608
0.105787214
0.362154613
0.287773722
-0.183592077
0.507301765
0.170799794
0.175199761
0.170125546
0.177018436
-0.156307054
0.322269466
0.326094832
-0.12481954
0.453368599
0.292952904
0.177880383
0.577858207
-0.260238538
0.164718965
0.20577943
0.116711836
-0.155138832
0.214030679
0.327615977
0.157123034
0.1179537
-0.108927338
0.119543674
0.229946723
0.386121652
0.121665971
0.136757509
-0.256881701
0.498381564
-0.213015437
0.298964576
0.229612283
0.19175783
0.443062122
0.178273046
0.368267688

0.109832284
0.175224708
0.149076347
0.243614023
0.100229131
0.293961725
0.109772367
0.379034078
0.491335593
0.12779158
0.362297842
0.523584777
0.147775857
0.182484745
0.283618359
0.20713835
0.297759753
0.141405177
0.106585269
0.148413167
0.134711942
0.154349715
0.330285707
0.456446552
0.292281659
0.233369504
-0.278976478
0.123022318
0.163727507
0.345874874
-0.147656059
-0.117512999
0.128719079
0.304439656
0.233078072
0.298154126
0.111331826
0.342474189
0.197580221
0.309009368
0.311914561
0.554673295
0.148703498
0.133501893
0.114291795
0.169509132
0.103151351
0.176068577
0.142241122
0.441752734
0.202421327
0.422458446
0.198092075

0.190859115
0.167430824
0.138920167
0.139017781
0.11279534
0.461719713
0.249523406
0.198518154
0.208144916
-0.109597717
0.101209369
0.108412193
0.436326704
0.533313796
0.168022197
0.205132522
0.218313556
0.231697224
0.114523227
0.144850364
0.329338114
0.23491187
0.118303963
0.136252914
0.101307229
0.117431715
0.258034117
0.338798897
0.291556668
0.142150369
0.196906797
0.231016078
0.324863695
0.444477652
0.341966617
0.308070367
0.133543758
0.205704607
0.189550225
-0.213329326
0.144274492
0.340586163
0.281985571
0.105031162
0.165350265
0.119720804
0.189472909
0.398293435
0.191388743
0.380589036
0.156019654
0.488396271
0.205503703

-0.147094191
0.15188688
0.374329933
-0.121072841
0.149559018
0.219243916
0.125571328
0.149137981
0.110383921
0.344849306
0.100325855
0.110134276
0.194521924
0.21823303
0.20326934
0.143625761
0.247090798
0.355956399
0.333722169
0.37526059
0.257440406
-0.100173352
0.112641806
0.137242795
-0.207146234
0.58063369
0.125091071
0.211479799
0.178592523
0.348018295
0.114003944
0.111169431
0.247319801
0.149088656
0.101977946
0.319703722
0.120805623
0.249685375
0.386316171
0.169527443
0.234606069
0.377181472
0.334223782
0.235541812
0.244360375
0.196452445
-0.109845841
0.185928335
0.210907825
0.185031169
0.142884411
0.12937179
0.118508449

0.167165386
0.182939568
-0.209215248
0.141184362
0.185384421
0.136433968
0.136715929
-0.163034182
0.158602001
-0.279736773
0.130762185
0.449781714
0.193500928
0.152891579
0.296051086
0.303673207
0.216746044
0.313641377
0.116193266
0.102323
0.381432877
0.340888996
0.43156927
-0.209817644
0.3767314
0.314132459
-0.166952818
0.339308982
0.117296317
-0.220781665
-0.261058039
0.302177831
0.513699353
0.329613396
0.171822454
0.102874846
0.112887542
0.234454954
0.198864198
-0.15441249
-0.140151795
0.348245735
0.271401675
0.274469019
0.516375088
0.113799541
0.323439851
0.196539837
0.138391826
-0.16760304
0.18225085
0.173305471
0.428491155

0.14321188
0.360088085
-0.192787364
-0.233430891
-0.134026948
0.283687387
0.181568223
0.236444536
-0.184268588
0.121305517
0.248591357
0.17774366
0.112885014
0.404947204
0.27405906
-0.24013169
-0.153613699
0.190560691
0.147854767
0.358506273
0.169670067
0.196897828
-0.160723197
0.35876276
-0.236533644
0.142032889
0.151865863
0.30633311
-0.164318908
-0.122966867
0.359993736
0.416790555
0.416940396
0.239833429
0.537480052
0.234533436
0.10111159
0.130340344
0.108705705
0.117721101
0.191953199
-0.205228134
0.114315803
0.194446352
0.326702671
-0.224732562
0.171090105
0.124316015
0.273997551
0.448507061
0.30849303
0.174330244
0.163312027

0.116853177
-0.22356418
-0.132373801
0.160456352
0.179765773
0.342145112
0.528897367
0.34292157
0.396307951
0.143305127
0.118244778
0.206432394
0.156777892
0.370988974
-0.12581178
0.296764788
0.119595868
-0.16276828
0.100879711
0.236316088
0.106569649
0.286729255
0.112325632
0.142059024
0.427835545
0.151525898
0.344382342
0.309017151
0.344156816
-0.167275741
-0.228201057
0.102217255
-0.251862528
-0.16521919
0.123170798
0.334766737
0.427697781
-0.204339792
-0.164668058
0.151676249
0.111904714
-0.242843602
0.371520427
0.116518301
0.124714509
0.19466026
0.135688979
0.115706695
0.376058513
0.271914853
0.476914064
0.158634078
0.473428517

-0.101125721
0.224493772
-0.184136122
0.184634356
0.224680734
0.422399532
0.411265081
0.341665736
0.119285147
0.206641213
0.392830717
0.294010523
-0.20032667
0.110693191
0.213571299
0.201975
0.119977776
0.238671523
0.14042746
0.228935449
0.232606253
0.457466615
0.113048265
0.106584549
-0.203123965
0.132382007
0.168136127
0.367788572
0.123155973
0.151481839
0.193534962
0.159786736
0.177312522
0.151084099
0.209698054
0.12866035
0.112233991
0.11475968
0.481864411
0.113251561
0.115376294
0.129844505
0.119339705
0.216166547
0.30746332
0.131904378
0.534785092
-0.133896708
-0.403437984
0.352218273
0.173901001
0.180190583
0.308116886

0.309593501
0.120874319
0.495290039
0.282906683
0.197110009
0.125308798
0.276871543
0.373937713
-0.166370212
0.322285283
0.151704625
0.400045331
0.410852267
-0.156659388
0.405320415
0.162452267
0.311771394
0.144103888
0.118428527
-0.273936815
0.13956444
0.203878515
0.334943391
0.23535403
-0.176240102
0.307684613
0.358158029
0.365092617
0.251128445
0.17472967
0.158225548
0.162023024
-0.102418346
0.170297996
0.118004391
0.138942694
0.193288799
0.346540941
0.261902431
-0.235896742
-0.157471053
0.447698723
0.161338723
0.323563116
0.39436958
-0.239532004
0.174467463
0.121493503
0.112193452
-0.260242596
0.400534846
0.105326907
0.305104771

0.284480757
0.102032026
-0.126676305
-0.129412193
-0.305311408
0.173718279
-0.149932071
0.105569354
0.339608743
0.139011239
0.118842762
-0.1567808
0.123655686
-0.255072149
-0.129689154
0.333042106
0.154238025
0.25172962
0.232509116
0.162421371
-0.277717664
-0.297106656
0.348672951
0.219838011
0.15767795
0.148284265
0.187669665
0.533138674
0.257499463
0.207373543
0.198252743
0.105396526
0.115585533
-0.202179688
0.244428443
0.292586605
-0.233774158
0.423179413
0.490995572
0.44149052
0.331039077
0.150829743
0.125695873
-0.124983187
0.183957322
0.379023775
0.330585475
0.168858977
0.111238339
0.123779076
0.20601769
-0.2123678
0.142256497

0.113639562
0.124542765
-0.131659506
0.212189929
0.380560858
0.191969974
0.102355111
0.26310668
0.157175624
0.131418464
0.291553396
0.181300224
0.165413853
0.179132992
-0.288105095
-0.197940235
0.39719725
-0.28503104
0.369128488
-0.279038985
0.243826528
0.113054927
-0.212765451
0.139047032
0.127969924
0.162941021
0.129809473
0.385535092
0.350184903
0.215025403
0.104853867
0.119791338
0.132994902
0.131178761
0.118032569
-0.117014652
0.127420321
0.163708504
0.163844096
0.207426909
-0.177733547
0.52248764
-0.191210309
0.128066368
0.234341314
0.129847036
0.164009338
0.181634037
0.114953669
0.163630952
0.191001928
0.177691977
0.158773649

0.16085545
0.22401446
0.389625228
0.370959532
0.345647126
0.191952367
0.30964313
0.185902966
0.35045587
0.514286775
0.527111885
0.183276756
0.268769903
0.119968579
-0.11800907
0.351616659
-0.171450366
-0.28639973
0.351231587
0.238797268
0.242886132
0.197476331
0.194647068
0.398266537
0.263173243
0.20507508
0.297582842
0.429225862
-0.134349931
0.265716879
0.486600696
0.149324938
0.19477776
0.207062872
0.115318488
0.117888787
0.107336936
0.185705039
0.118836239
0.149188012
-0.266665582
0.478862742
0.378548978
0.145366012
0.211486567
-0.286740108
0.226945696
0.429722214
-0.104850073
0.257771808
0.137303476
0.254467651
-0.158698325

0.339271618
0.392736056
0.332680341
0.395444355
0.501301611
0.433175936
0.44578821
0.17548138
0.218734075
0.121131775
0.184264181
0.112836881
0.274147922
0.218851026
-0.138556327
0.137128337
0.108644627
0.367695254
0.20891979
0.268732147
0.114506371
0.422875254
0.436497273
0.364967739
0.459527705
0.191494689
0.37712672
0.209094065
0.138095559
0.204276263
0.111172399
0.11969411
0.298695102
0.155980562
0.110232221
-0.193641737
0.331084765
0.134306313
0.42815733
0.489213875
0.205096716
0.179262428
0.13630566
-0.288134917
-0.208853566
0.222360776
0.212023998
0.222280441
0.450892947
0.194372646
0.152510091
0.155907078
0.113119501

0.320472179
0.231541959
0.221461993
0.14031919
-0.150040896
0.125226721
0.212872842
0.137057298
0.234745924
0.346657425
0.286300879
0.150483885
0.2542952
0.13963078
0.185515501
0.508134875
-0.14139358
-0.295836257
0.358776877
0.339263022
0.364557023
0.132355379
-0.175985982
0.202502527
0.269818163
0.306392107
0.423524629
0.351069128
-0.141035829
-0.126408695
0.24851665
0.348412278
0.237411433
0.362733434
-0.166423719
0.109293131
0.323167775
0.303381655
-0.234268572
0.261015836
0.283848345
0.267321822
0.210487447
0.291390794
0.528247195
0.208598619
-0.200613421
0.265985371
0.156716278
0.128542913
0.224334345
0.325150744
0.289859889

0.480260951
0.178350631
0.332152714
0.243044365
0.134900158
0.221608125
0.17458333
0.153082901
0.103057268
0.408444657
0.254330918
-0.258462206
0.338269722
0.320446298
0.338084702
0.317586521
0.117294016
-0.26193871
-0.108413431
0.237499083
0.128266192
0.183512629
0.10866598
0.235164551
0.151730571
0.372100179
0.192955082
0.202141179
0.197629238
0.132376381
0.281098325
0.127349931
0.233643184
0.229436389
0.150430918
0.168500511
0.128207907
0.112164379
0.275540489
0.148024217
0.220441167
0.287677659
0.143267529
-0.109849492
0.297246184
-0.198876396
-0.112546642
0.150569736
0.131691662
0.278758668
0.280354458
0.306335159
0.314635894

0.207736304
-0.161460363
0.206520585
0.288090684
0.243371944
0.512256041
0.349656244
0.105022266
0.274574803
0.136531296
0.11414488
0.496531221
0.138537777
-0.109364617
-0.180820281
-0.188018136
0.126615195
0.117798091
0.302966497
-0.350700202
0.145137435
0.307062593
-0.175816111
0.114200747
0.107177796
0.186855778
0.222337896
-0.275050816
-0.316077372
0.186444052
0.138536998
0.196507704
0.18204921
0.270499405
0.214524517
0.211548197
0.385225077
0.374611899
0.500690623
0.284078161
0.363501732
0.246686014
0.147843004
0.185602146
0.17435603
0.134756645
0.311567307
-0.110400453
0.436825662
0.228641951
0.27867666
0.370857941
0.121974254

0.29248734
0.116470169
0.162673014
0.102618804
0.184246606
-0.101624652
0.192027408
0.129240829
0.44546276
0.176010653
0.391840815
0.12132314
0.105646293
0.411954073
0.556502975
0.107769732
-0.140836186
0.116080238
0.242681941
0.180030465
0.266301998
0.213870922
0.213681398
-0.100525576
0.116645835
0.205548128
0.285303889
0.199704649
0.168542767
0.192455675
0.116276898
0.150879974
0.483326219
0.536481573
0.349500343
0.388257323
0.344034971
0.185230498
0.255878137
-0.269093895
-0.105166335
0.299389317
0.36447364
0.183871779
0.174175572
0.419410876
0.465954392
0.159612111
0.266676157
0.369238666
0.43431653
-0.201944952
0.36001378

0.276490765
0.169581085
0.270422848
0.141148717
0.114789147
0.371107395
-0.272123146
0.119442291
0.108705942
0.444089667
0.548982593
0.172293703
0.450116493
0.14658293
0.103643665
0.321731217
0.207856905
-0.254631144
0.368599777
-0.194448342
-0.12703211
-0.112431846
0.537814662
0.432065322
0.391872707
-0.112776331
0.451134859
0.197102038
0.164403163
-0.185905678
0.306799016
0.313824894
0.362558716
-0.13694054
0.158941639
0.324990115
0.193274987
0.361479872
0.131520563
0.113199092
-0.356123822
0.301155599
-0.377301015
0.12496808
0.529191499
0.389255172
-0.116158384
0.465106384
0.386822871
0.143013067
0.428715724
0.407491856
-0.366610661

0.20597366
0.230849354
0.117447889
0.272839562
0.171322368
0.278251855
0.383344298
0.245792351
0.118757518
-0.19340979
0.362045914
-0.156064768
0.126929452
0.110527779
0.304232013
0.245524576
0.28943234
0.14602869
0.188839557
0.116692545
0.116632637
0.160218055
0.177262567
0.174757359
0.106638325
0.110295701
0.292783047
0.357551659
0.268064062
0.19961048
0.352768653
0.176882924
0.300076876
0.119266222
-0.348762715
0.222012768
0.336295742
0.281980266
-0.216297449
-0.105667037
0.27523226
0.124236256
0.171574112
0.163843374
0.121145997
0.108124661
0.174927638
0.139539347
0.214883349
0.476424566
0.118917446
0.109560038
0.157052521

0.324896126
0.150586271
0.138681369
0.209182768
0.212052448
0.185043806
0.101407798
0.111057427
0.494023858
0.414810816
0.152482788
0.148136325
0.372166956
-0.170662524
0.333045332
0.251991486
-0.254373956
-0.310144439
-0.149644673
0.110411232
0.268088602
0.138851406
0.21494418
0.403034915
0.429357952
0.411691453
0.551095086
0.488509564
0.167650667
0.139970521
0.373303147
0.308585991
0.123450792
0.118681799
0.125021457
0.360902061
0.15884046
0.289204328
-0.294259963
0.201647965
0.142260882
0.138973413
0.341423589
0.180424924
0.452923425
0.201051452
0.191496521
-0.203080655
0.227276706
0.105487887
-0.207944054
-0.262208742
0.159339941

0.186585602
-0.126046467
0.171554803
0.117772259
0.215580995
0.264013061
0.259592649
0.174119468
0.307989335
0.142475439
0.370575775
0.148804663
0.200007946
0.195388038
-0.157344808
-0.115071872
0.329225171
0.166940136
0.166041821
0.421417359
0.177309094
0.13640501
0.136191377
-0.180287926
0.117757423
0.357838196
0.445760537
0.29097269
-0.184708881
0.202878226
0.143749
0.120853831
0.467263569
0.224090235
0.222242361
-0.15525924
0.253463044
0.151297077
0.162077254
0.276964934
0.116330585
0.277263243
0.132754579
0.158681253
0.201712501
0.125874503
0.212596723
0.180932813
0.158272827
0.326877039
0.296208449
0.310017074
0.244228435

-0.186996708
-0.337531534
0.158209108
0.330504402
0.370541623
0.158350209
0.12529826
-0.249446054
0.427916439
0.146991558
0.18309173
0.269802021
0.348135714
0.165150852
0.460868396
0.332397032
0.113400898
0.252121335
0.121598813
0.229957467
-0.111730928
0.133344309
0.173290993
0.150203572
0.176340561
-0.12841088
0.3047761
0.256066816
0.172889925
0.135325093
0.184695145
0.303067833
0.118863726
0.25151221
0.119160345
0.274808936
0.182113285
0.262147223
0.230445293
0.275160933
0.300519772
0.238821717
0.126372706
-0.149699038
-0.208497557
0.356842291
-0.102306724
-0.12263924
0.189033973
0.235047034
0.188013564
0.173554178
0.419118212

-0.27568123
0.123614475
0.178107855
0.190475417
0.48240879
0.117773465
-0.128679294
0.226053782
0.472907458
0.393241017
0.126780047
0.502014436
0.20354449
0.105744709
0.397822087
0.461070084
0.320111546
-0.106070443
0.206288189
-0.113041315
0.181622274
0.138266606
0.203442934
0.142402888
0.290514344
0.2307841
0.3373238
0.141377006
0.176764757
0.172792335
0.363835131
0.283213182
0.351484825
-0.114410744
0.308155246
0.187895496
0.2046501
0.111162793
0.136410814
0.36654293
0.257196195
0.183887549
0.175901099
0.414814973
0.212466652
0.232643286
0.231595702
0.122742101
-0.12887488
0.177864919
0.156022216
0.183876238
0.29847426

0.395328676
0.107471575
-0.133478731
0.197731051
0.117463283
-0.14181576
0.1442065
0.476318399
0.403081076
-0.141910253
0.134193924
0.101590439
0.309765518
0.313370336
0.16082741
0.310929226
0.15149372
-0.123888778
0.22430838
0.123994913
-0.372267621
0.356561256
0.493487174
0.213239268
0.114895791
0.255162758
0.148724557
0.518172916
-0.120345358
-0.182553782
0.55915536
-0.171695039
0.275504895
-0.202291463
-0.118951783
-0.196870969
-0.259035902
0.199730504
0.355922056
-0.133409588
0.484082235
0.307932958
0.223546361
0.233798677
0.174016381
0.264085479
0.36710567
0.318131322
-0.18222113
0.475544226
0.33571508
0.131270857
-0.233884524

0.192234553
0.25084464
0.278268052
0.435983589
-0.340050451
-0.151611148
0.142234189
0.121950316
0.403895412
0.338787093
0.436706156
0.365225777
0.439666573
-0.158778135
0.410233246
0.43877399
0.160679897
-0.328629253
0.136143035
0.202994698
0.138198778
0.328424181
-0.10142011
0.10223431
0.325093111
0.210763762
0.309722162
0.111707088
0.188280598
0.450508966
0.201131466
0.365944841
0.168005812
0.173190471
0.113815807
0.296649293
0.307532066
0.13930002
0.137199464
-0.196809074
0.20463629
0.259460092
0.255541504
0.316012774
0.233286475
0.326267955
0.237777264
0.23020426
0.364994165
0.174848494
0.118444462
-0.223469293
0.109091308

0.248295735
0.155899807
0.104018292
0.101725175
-0.132959991
0.122386557
-0.11098918
0.167187016
0.143368163
0.116654191
0.509311667
0.170704935
-0.323381793
0.240777399
0.192412831
0.268894726
0.333763911
0.259490479
0.393600146
0.21005505
0.196481364
0.147538283
0.140076173
0.239517314
0.16449019
-0.174646295
0.24805862
0.202494945
0.297029824
0.360153805
-0.175665822
-0.225593119
0.135071362
0.269004897
-0.182913175
0.133518454
0.180286357
-0.19425753
0.151709027
0.134335875
0.119342783
0.223435789
0.160997479
0.317333017
0.480292326
-0.1446291
0.399757357
0.121982133
0.245844935
0.223013901
0.124402952
0.16877074
-0.291655718

0.120973616
0.113616852
0.139631051
0.391858166
-0.215968625
0.273444918
0.385117524
0.164123382
0.194665513
0.176593259
0.140516023
0.144286972
0.122660895
0.252585614
-0.121968955
0.375030236
0.334311667
0.317105964
0.263580987
0.119931345
0.437189328
0.168216692
0.29055247
0.332354192
0.150386763
0.326246757
0.155148316
0.100343072
0.143944636
0.286791531
-0.188473706
0.133749536
0.313737412
0.446200332
0.147718077
0.210261793
0.175414853
0.145658992
0.125638942
0.215439433
0.149369431
0.231610333
-0.186852911
-0.264673116
0.296517019
0.312630518
0.252873511
-0.161966703
0.120708206
0.180030437
-0.247034247
-0.161201019
0.348102738

0.102371402
0.493032324
0.144353066
-0.140429739
0.234016921
0.152002668
0.239851945
0.437340076
0.492818902
0.247528839
0.284500044
0.441430198
0.241633854
0.122368566
0.422282628
0.114010383
-0.200610584
0.157783969
0.18052558
0.446016946
0.155529597
0.473920791
0.124305348
0.389656262
-0.10246889
-0.168142356
-0.295008398
0.213823669
0.34249678
0.217800894
0.157554859
0.111468518
-0.100852317
0.208947456
0.401886131
0.124643795
0.265167908
0.184371052
0.143604702
0.107960301
0.100700954
0.107197147
0.205292822
0.10927762
0.148337253
0.169793161
-0.301721491
0.206598528
0.215036668
0.156341805
0.391949196
0.492355629
0.190131305

0.12757537
0.107080021
0.214192698
0.123016946
0.302971075
0.155731933
0.137671549
0.126439183
0.187556799
-0.294820521
0.176890769
0.193986167
0.256184114
0.159245127
0.409126353
0.135366961
-0.177405292
0.12669433
0.196173981
0.212084753
0.167407544
0.149710495
0.218633774
-0.114183824
0.167459257
0.450954407
0.110757253
0.16100898
0.250677278
0.256350432
0.435958871
0.124760294
0.206499745
0.114429658
0.23973769
-0.119390568
-0.107945799
0.142698834
-0.137445038
0.292763106
0.374832394
0.419257325
0.124828886
0.23574286
0.45913329
0.232447484
-0.22632143
0.21465433
0.194600233
-0.192164286
-0.223350959
0.215514282
-0.211843549

0.153915024
0.307322353
0.238296977
0.140492351
0.205594244
0.29363997
0.328065869
0.449170365
0.114514415
-0.136122155
-0.143498299
0.149206785
0.15761242
0.373110792
-0.169918767
0.310033087
0.128210083
0.130712903
0.141475847
0.116815597
0.279497794
0.250245744
-0.14730378
0.262377135
0.246522748
0.207335082
0.150363012
0.531706809
0.242866585
0.28546756
0.413942687
0.293798558
0.267899458
-0.116386532
0.136921705
0.204714805
0.194212636
0.280914189
0.131205691
0.210442349
0.155746837
0.109918917
0.207747129
0.12248817
0.190502152
-0.111308309
0.354812489
-0.120838
-0.114546896
0.106852687
0.317060669
-0.118601323
0.259041116

0.220607914
0.141706555
0.399813872
0.14516532
0.143648994
0.310242814
0.15511423
-0.273051223
0.416212725
0.214337133
-0.154417123
0.431813913
0.197340633
0.27892213
0.157248538
0.103508259
0.186592258
0.312079757
0.167299691
0.410025693
0.52033936
0.244693225
0.107976486
0.12661053
0.100294886
0.212655125
0.103724831
0.193116447
0.140904457
0.232150251
0.151189405
0.162996237
0.225371848
0.238829843
0.261137134
0.372927554
0.421322369
0.123674299
0.474710583
-0.111811237
0.222454775
0.160033458
0.127609747
0.161556538
0.295796886
0.13723045
0.207521225
0.364008462
0.267584084
0.214981476
0.282506932
0.206161098
0.323554853

0.122070473
-0.247921942
0.336868054
0.252684438
0.315497543
0.132626298
0.26889649
-0.247514156
0.105733623
0.278017104
0.436599403
0.366508713
0.25310115
0.126582317
-0.20196466
0.155504078
0.219946536
-0.105314243
0.11888641
0.100088413
-0.122448249
0.268743009
0.295231661
0.115538941
0.194526887
0.153625474
0.524278285
0.147171001
0.184633108
0.416703076
0.231036955
0.108725063
0.210694703
0.263668383
0.312252141
-0.279669231
0.138140789
0.164026909
0.11488722
0.182806087
0.243462155
0.138633603
0.466680329
0.190164715
0.14964217
0.156161166
0.144232656
0.156953176
0.255873128
0.160075563
0.305662285
-0.12036278
-0.154507985

0.351621046
0.187290497
0.292368141
0.372981515
0.26159636
0.20162111
-0.169206673
-0.142033736
0.104093166
0.120998817
0.160849655
0.110954947
0.107107894
0.463169289
0.245504556
0.316764961
0.280658534
-0.236435271
-0.162425331
0.132935783
0.202391443
0.109891659
0.251689706
0.421378403
0.209090514
0.144654473
0.197028942
0.130986723
0.393410951
0.359582529
0.124219262
0.19927509
0.266825091
0.483786123
0.42845668
0.466729537
-0.135063483
0.193946896
0.365502308
0.385903857
-0.121935535
0.112323905
-0.155270184
0.133649478
0.132155035
0.114162864
0.116021611
0.381768178
0.168662858
0.157455818
0.119643953
0.193432295
0.157593653

0.104691578
0.537239536
-0.177390505
0.212980735
0.369446849
0.38472647
0.217212781
0.238736688
0.101926785
0.316611912
-0.135884761
0.281346823
0.145992698
0.224230021
0.119349581
0.171739289
-0.102345992
0.311303979
-0.131413458
0.378455876
0.119360518
0.159268418
-0.105128696
0.21421582
0.134512515
-0.101492852
-0.105435849
-0.104950723
-0.226255462
0.147417821
0.253779358
0.145376938
0.181896467
0.391248569
0.193561096
0.136757916
0.327507896
0.316164966
0.11930825
0.374108102
0.125083067
0.380138027
0.265914187
0.187168781
0.451811547
0.143773221
0.114960331
0.149081507
0.219979041
0.183361156
0.255253534
-0.321644589
0.265610241

0.45179133
0.133527214
0.25212721
0.144582696
-0.131239982
-0.327680474
0.361074538
-0.102840794
0.397458941
-0.145120621
0.184518824
0.144807679
0.58129485
0.210343299
0.149631787
0.142577508
0.363948602
0.154383407
-0.180286942
0.120672506
0.102894857
0.16632142
0.130568201
0.175479015
0.225002467
0.285739287
0.123433664
0.298171356
0.318836516
0.188630475
0.291606767
0.144998668
0.297154086
0.178164505
0.146458537
0.113884608
0.12353392
0.190237628
0.162742135
0.403977187
0.272370308
0.37679976
0.147526304
0.182969416
0.107897523
0.191410987
0.132961649
0.456371487
0.185087634
-0.28614587
0.121851046
-0.131227028
-0.229350921

0.135072103
0.187412817
0.277350364
0.252651928
0.415687836
0.123621116
0.299915783
0.252363182
-0.10616814
0.321642267
0.187857674
0.116182211
-0.140223605
0.195306132
0.188714674
0.121136694
0.127291281
-0.172678852
0.180530753
0.398061546
0.265886136
0.178321246
-0.246449793
0.398279423
-0.101915401
0.160342552
0.116633025
0.18024104
0.107613934
0.283383403
0.347366395
0.102795012
0.259373648
0.154946599
0.342377638
0.337354562
0.119186101
0.125179843
0.105846405
0.172328945
0.159826874
-0.170827959
0.166910846
0.144237039
0.370532533
0.203268227
0.243423251
0.238780907
-0.173928221
0.121798902
-0.179712801
0.381427192
0.339349264

0.212943721
0.105573606
-0.162767341
0.147861691
0.507634294
0.390072658
0.543523849
0.127321897
0.163570139
0.265699595
0.176843666
0.227114738
0.583132709
0.43483557
0.289999911
0.447500394
0.291297853
0.301041158
0.218941721
0.308947088
0.207510493
0.410282772
0.207002201
-0.11926336
0.218837652
0.152519198
0.369339796
0.172457368
0.198701761
0.388812467
0.27093998
0.138634041
-0.141335379
0.124901214
0.466950309
0.244251035
0.463801541
0.5224646
0.179198754
0.313173476
0.168305373
0.162877524
0.111754716
0.141332906
0.162901466
0.142397067
0.220908183
-0.19076878
0.494330631
0.300209588
0.462463035
0.14349143
0.200963773

0.494106126
0.121055573
0.165723084
0.175986543
0.25115539
-0.121642084
-0.11327016
0.174395689
0.403433074
0.102120878
0.261748323
-0.16193114
0.180076323
0.408016377
-0.159222438
0.26835007
0.251426439
0.253009665
0.109555215
0.123549082
0.117668883
0.416746501
0.219030845
-0.124173003
0.122127732
0.146298859
-0.112558676
-0.130919417
0.41745652
0.291406549
0.24908082
0.274762604
0.101167605
0.275266284
0.166921555
-0.150379428
0.266456182
-0.199024166
0.152946283
0.285533392
0.320556318
0.301125379
0.227560848
0.271340633
0.321672101
0.160565746
0.344978729
0.112776869
0.330855162
0.306181895
0.328197125
0.268393054
0.333720307

0.34180724
0.112864879
0.22713914
0.19901704
-0.113317585
-0.113573449
0.505307608
0.127047795
0.301064465
0.372313002
0.316504864
-0.136920209
-0.131619942
0.349957083
-0.293279725
0.235526896
0.11370436
0.146387645
0.156312451
0.129244451
-0.108417176
0.276403205
0.124807546
-0.383191068
0.233606978
0.17734129
0.482483727
0.477618483
0.207204231
0.270185827
0.127771277
0.37515236
0.234933106
0.457611553
0.211091847
0.157194524
0.283554322
0.101104915
0.122426286
0.506569
0.134068507
0.354299895
0.257682368
0.349584818
-0.190247697
0.29461977
0.395628419
0.462162208
0.200498931
0.204096412
0.421385094
0.13493679
0.269102299

0.212006198
0.173126957
0.136178566
0.179834335
0.120066967
0.4876733
0.339267911
-0.124333547
0.282046663
0.317071001
0.118583543
0.205448917
0.127278077
0.437657905
-0.460747662
0.21568633
-0.107085593
0.107375015
0.253535981
0.488349895
0.470703685
0.156575076
0.229661246
-0.104033415
0.299982335
0.396963443
0.213438047
0.102706548
0.107303915
0.219883297
0.145643599
0.140234448
0.292116235
0.107226207
0.114166476
0.117819528
0.236287599
0.173738223
0.282819897
0.431540904
0.290002175
0.101838963
0.434822381
0.251653185
0.204314421
0.283725429
0.456496501
0.523030721
0.204576102
0.211079258
0.206429837
0.109564181
-0.126244929

0.10706817
0.106956484
0.183264425
0.149032435
0.193555956
0.224951412
-0.240916993
0.442664594
0.404124861
0.291558762
0.106923836
0.186648496
0.216320882
0.128497461
0.373846667
0.207556827
0.423088129
0.374144981
0.421272255
0.111241928
0.161832423
0.256597903
0.165616044
0.151342117
0.50301558
0.116495476
0.100266503
0.16683935
0.127350018
0.527324391
0.143528929
0.102357081
0.240122737
0.362110733
0.105127141
0.168326505
0.181288464
0.159456489
0.135023842
0.241890942
-0.111775679
0.119419365
0.119266184
0.122306828
0.221260692
0.238567131
0.188806988
-0.105739196
0.321420068
-0.17705459
0.253633351
0.201949481
0.199303208

0.138902678
0.141308025
0.144097502
-0.168244324
-0.204350051
0.207237936
0.466740645
0.113663859
0.126395549
0.123432043
-0.109701839
0.198275788
0.157656098
0.100797296
0.119002581
0.222016305
0.254746081
0.214434581
0.151918905
0.281443164
0.245889571
0.11266903
0.142453961
0.296252927
0.136096445
0.25845548
-0.191896344
-0.130285186
0.207255569
0.129996156
0.327133177
0.459458802
0.220658337
0.202147369
0.387920315
0.226883571
0.147452246
0.182829926
0.181308036
0.238654847
0.143763383
0.103818973
0.331171204
0.273158469
-0.267112713
0.444598109
0.176540623
-0.111551131
0.114197899
-0.14657718
0.105766911
0.339637494
0.477434065

0.2809678
0.173241937
0.223651873
0.34552328
0.178814636
0.377667537
-0.182118431
0.178934538
0.138884226
0.144426275
0.111455775
0.232156008
0.258821801
0.156520729
0.217887015
0.136877074
0.152047172
0.179092864
0.228909688
0.221629044
-0.165261289
0.370569101
0.373855554
-0.193626642
-0.165462263
0.27672316
0.324941867
0.308642853
0.154384099
0.151182127
0.162582819
0.435590929
0.170472859
0.318940334
0.452851026
0.408384263
0.105943102
0.14749636
-0.166603056
-0.152113392
0.206016285
0.130139317
0.253904054
0.264449945
-0.136024512
0.276605452
0.146091124
0.197855748
0.341596981
0.265058319
0.106530658
0.260077411
0.13799197

-0.151107063
-0.245702423
0.119148672
0.153528012
0.149103783
0.106643597
0.100174136
0.232127154
0.112786061
0.175802275
0.133774207
-0.169304152
-0.206207442
0.216335973
0.371434508
-0.108357775
0.173466661
-0.223980314
0.289344653
-0.16555579
0.409644923
0.225428974
0.121773738
0.164843578
0.116249273
0.180088864
0.300722756
0.390592767
0.11414397
0.193587067
0.132815337
0.181359627
-0.178360369
-0.238783243
0.214122175
0.133409528
0.119013394
0.154647694
0.399855022
0.298859345
0.372204097
0.584173146
0.368468734
0.113396065
0.283341655
-0.237813604
0.190779389
-0.132965908
0.203127089
0.217576891
0.185127747
0.292160948
-0.101257587

0.298171247
0.308273111
0.13856845
0.224230937
0.154773374
0.373071709
0.367168036
0.261432914
0.17098597
0.433830129
0.265010918
0.241709243
0.193944734
0.112463917
0.507236974
0.193825757
0.101626779
0.151989381
0.12468367
0.1874174
0.229731276
0.218483569
0.135052241
0.157338809
0.190112629
0.104762839
0.177021721
0.113536224
0.292659052
-0.118228911
0.282160141
0.458730862
0.10271066
0.381851923
0.188247555
0.412295898
0.374732432
0.295825136
0.116592336
0.17395595
0.378467707
0.186767973
0.142824429
0.24229374
0.299095127
0.115771609
-0.285087867
0.330277509
0.130215414
0.106306985
0.297740975
-0.181165067
0.468173292

0.30587822
0.464668769
0.152237273
0.119126417
0.224916201
0.19675709
0.164073875
0.110940183
0.111402749
0.21178328
0.105035039
0.129759371
0.317212215
0.182457022
0.104479597
-0.131635116
0.130211649
0.122061208
0.106391405
0.22453203
-0.119181113
0.21940774
0.474169469
-0.143538223
0.234930276
0.17499573
0.260137953
0.205707089
0.124311915
-0.108276691
0.238949485
0.125805138
0.141892844
0.388832119
0.30251771
0.137298223
0.105724698
-0.274271858
0.342410638
0.353872756
-0.140604654
0.144404155
0.136494474
0.136845669
0.142537792
0.28964264
0.181750039
0.196939578
-0.122446712
0.128637117
0.110158635
0.10464601
0.171188479

0.13480795
-0.228743803
0.451457761
0.2128187
-0.356777171
0.393473894
0.297890602
-0.128477705
0.11960445
0.155832405
0.26053063
0.3732721
0.296872245
0.132584956
0.111898512
0.144189827
0.238993945
-0.212284496
0.100489753
-0.149318275
0.362446486
0.115848603
0.383389778
0.309829666
0.392857846
0.320406447
0.436389482
0.486628587
0.109754824
-0.110156211
0.345033264
0.370318913
0.16600685
0.126787637
0.390517397
0.231619582
0.288927483
0.243806298
0.358336143
0.198044555
-0.129457475
0.179843629
0.260097062
0.122091785
0.137570497
0.134563716
-0.190379293
0.107864847
0.202358029
0.126355311
0.326736612
0.230105661
0.221858831

-0.125336807
0.128627856
0.152925478
0.101263925
0.162370325
0.10968274
0.487490802
0.406525567
0.236698072
-0.291838963
0.450219272
0.112412565
0.105896909
0.145539544
0.241319231
0.324884754
0.204867
-0.16397209
-0.101949051
0.2731065
0.412495561
0.358644243
0.259290329
0.149530499
0.340736422
0.332636585
0.128625233
0.296196788
0.134919668
0.16676151
0.316107882
0.279915016
0.134273601
0.128488799
0.152904535
0.283015042
-0.307210638
0.100990675
0.127315229
0.153075262
0.133898127
0.167147034
0.251613302
-0.114112181
0.513541304
0.527650716
0.521918494
0.10960528
0.102477104
0.396487491
0.187342732
0.258194896
0.214511936

0.380962351
0.230807344
0.252071524
0.105975208
0.210302694
0.253170372
0.130838612
0.364596717
0.232421821
0.121804541
0.11233517
0.115328032
0.330624604
-0.232835483
0.222722813
0.195347039
0.275487112
0.221392561
0.102138546
0.231541934
0.156145416
-0.192844975
0.117663053
0.155549293
-0.107100659
-0.2139293
-0.309067313
0.578250876
-0.250390608
-0.149198638
0.160300591
0.281459594
0.304506939
0.169501158
-0.272432737
0.223242236
0.23143085
0.256896342
0.375074337
0.160478388
-0.197225128
0.372743929
0.132905215
0.227052892
0.313263986
0.241690565
0.170839154
0.336616558
0.208993577
0.261648279
0.289259065
-0.264307516
-0.12929306

0.17804249
0.226642989
0.322884284
-0.120268385
0.31325657
0.50172984
0.195923302
0.40347557
0.132094481
0.110424177
0.17443522
0.5380321
0.101064324
0.195116593
0.142213126
0.276479109
0.104168653
0.138319433
-0.173191374
0.103795223
0.341270299
0.112002599
0.351232656
0.139719497
0.472063307
0.432237429
0.187739527
0.104665791
0.412903236
0.332634168
0.532497405
0.219297364
0.223736852
-0.168929239
0.479310721
0.300169038
0.258964588
0.104112176
0.112464015
0.175578415
0.194920483
0.187987842
0.407539152
0.149849419
0.438227372
0.10422886
0.175918112
0.120434804
0.126953588
0.180296417
-0.250388211
0.361854253
-0.304336761

0.461241574
-0.108035354
0.161641087
-0.13398511
0.293323486
0.113200364
0.169992978
0.130158749
-0.119170079
0.117471876
-0.189712891
-0.38883479
0.104156257
0.276651315
0.121601918
0.129442291
-0.115906929
0.116445939
0.124050377
0.126174515
0.17891268
0.394826802
0.363867992
0.160194305
0.251659062
0.355901464
-0.439903475
0.263685689
0.126205187
0.125041998
0.192246707
0.256736
0.127631063
0.116754606
0.116995275
0.134350903
0.151685623
0.299524829
0.161803826
0.139248161
0.36387955
0.27061095
0.339608738
0.121031633
0.294610857
0.36799737
-0.222679187
0.400655526
0.260775494
0.25069831
0.185503364
0.190274817
0.13834443

0.150237001
0.131405058
0.119799193
0.114068431
0.106010998
0.172311038
0.154456936
0.140466862
0.146131393
0.267829721
0.149412056
0.30027146
0.385010027
0.420620139
0.13304012
0.144358843
0.418268266
-0.138745836
0.359914619
0.374515935
0.347534169
0.420270283
0.135730164
0.286883498
0.379581779
0.102861981
0.138560347
0.137439558
0.226117796
0.22731843
0.465247531
0.229713938
0.167764784
0.221760286
-0.21395404
0.299434911
0.124848023
0.185919803
0.135083683
0.11714458
0.146100824
0.164599971
0.123867428
0.177789489
0.170701017
-0.119312817
0.128689992
0.344699272
-0.127741639
0.178586811
0.299181735
0.163554363
0.456628717

0.15849823
0.151404312
0.208326328
-0.240556959
0.217171613
0.151758442
0.115683742
0.121844202
0.119854094
0.207869853
0.144594906
0.223821576
0.176038189
0.418045702
0.406597954
0.221622053
0.244230022
0.142940936
0.162027144
0.442314464
0.218498665
0.130032218
0.166802383
0.178635111
0.394800294
0.112470627
0.301461574
0.142878599
0.261589783
0.136305137
-0.245188013
0.252662618
0.148338385
0.387685643
0.168485416
0.269098992
0.281662561
0.120171466
0.332956166
-0.103882174
0.146636559
0.155329084
0.119597647
0.29115317
0.151114474
0.163169099
0.141094279
-0.152277672
0.10807001
0.140340381
-0.247133005
0.336791547
0.134937006

-0.102955154
0.155707615
0.267041902
0.146525548
0.140210167
0.159951677
0.165819484
0.193368157
0.102100888
0.199070897
0.132173385
0.11527546
0.141385516
0.525177471
0.106943626
0.176313225
0.201525724
0.14288801
0.218902404
0.372153595
0.278240093
0.250704258
0.194026281
0.159495714
0.125185436
-0.180697351
0.12089783
0.176260803
0.244659268
-0.123751065
0.210373392
0.119569415
0.202404514
0.467912514
0.495232139
0.300634682
0.487622554
0.22111398
0.208441691
0.342691366
0.401007045
0.22539824
0.207298908
0.169080566
0.209434908
0.158358475
0.216399807
0.148595771
0.439550298
0.118243434
0.243965799
0.114631164
0.146473788

0.155801002
-0.153900322
0.156327694
0.46371151
0.321706312
0.220078988
-0.150355438
0.27288108
0.398845528
-0.154834288
0.48649933
-0.328987815
-0.249399123
0.126680471
0.23311544
0.261938825
0.139937838
0.106697298
0.156008315
0.145265329
-0.113672937
0.125495457
0.137627003
0.44166918
0.121563099
0.133880359
0.264747207
0.182267075
0.287825567
-0.254422158
0.256905435
-0.114056098
0.318113787
-0.187436533
0.133346287
0.104510947
0.17795076
0.247869434
0.131839895
0.312651587
-0.104405581
0.216202176
0.444367004
0.447097541
0.360477384
0.22995999
-0.26002668
0.288777835
0.312874343
0.205195808
0.355771046
0.292090808
0.182963272

0.113300891
0.21607615
0.127445516
0.55148612
0.156579506
0.160515395
0.200809464
0.213330752
0.110344007
-0.181416326
-0.120449148
0.288198488
0.201947192
0.274988531
-0.187659698
0.192417384
0.287785174
0.285251342
0.422517524
0.247985986
0.136025665
0.421121777
0.522118265
0.448068611
0.294852698
0.126290541
0.189023258
0.342752651
0.170451689
0.168813502
0.182541308
0.305328644
0.1164914
0.267897685
0.143365034
0.125847733
0.153385208
-0.160783759
0.358943612
0.300405267
0.335648936
0.259304454
0.383056614
0.108340563
0.349430877
0.458032362
-0.108634632
-0.122673462
-0.111970563
0.1958906
0.147512763
0.352480076
-0.164076204

0.198935283
0.266585729
0.116059264
0.220082765
0.170198462
0.134190706
0.14152308
0.203291545
0.195624572
0.329423478
0.186104664
-0.120967519
0.130595374
-0.189215283
-0.178485426
0.392235353
0.104579901
0.234245415
0.187621806
0.152522971
0.37112839
0.178825485
0.224379696
0.118935404
0.210461497
0.221907301
-0.138010078
0.199673133
0.125607478
0.455629823
0.278330774
-0.128583189
0.286674575
0.144855892
0.361491153
0.188240835
0.159458455
0.14896316
0.219284233
0.144183847
-0.335311782
0.208545703
0.111242915
0.101788304
0.139526707
-0.316544103
0.435093119
0.374109128
0.268703731
0.120218514
0.313265311
0.113706069
0.112541269

0.350321745
0.333956265
0.459855289
0.205276254
0.204546356
0.101488476
0.412989159
0.37552917
0.455154937
0.262310895
0.440786341
0.552145287
0.211884096
0.226029342
0.402279529
0.319746425
0.292141419
0.166148808
0.207972358
0.169929549
0.155073015
-0.118469987
0.300445999
0.111970372
0.271975516
0.418525653
0.163674694
-0.136053517
-0.184193927
0.179871147
0.327701112
0.16432288
0.234449247
0.149581935
0.206743574
0.213633967
0.151288328
0.149245961
0.110706022
0.198406196
0.181908306
0.192668457
0.320605988
0.243698392
0.145900133
0.293348612
0.138256942
0.102094753
0.119572817
-0.208580215
0.309961881
0.134409938
0.186609852

0.101782029
0.219589124
0.14716682
0.38638801
0.294789165
0.29774341
0.311488324
0.184364418
0.20208604
0.249971018
0.140515556
0.1056605
0.107160843
0.155823532
0.534970301
0.111829736
0.11820555
0.200140666
0.472291681
0.12768071
0.376644706
-0.159153428
0.173757211
0.289284647
0.246249946
0.138907079
0.106360911
-0.126320334
0.293838803
0.2259413
0.365127083
0.469216442
0.215861161
0.441638144
0.537536504
-0.223061779
0.222571723
-0.206972796
0.256973169
0.402014032
0.141623216
0.134580825
0.15260423
0.317295885
0.327269447
0.308373586
0.208155091
0.284677266
0.452431447
0.201408605
0.152350007
0.11694507
0.27332601

0.189891927
0.191783202
0.319316804
0.436227431
-0.128857388
0.195458938
0.186569493
0.105624216
0.245659129
-0.225785042
0.154055765
0.172947995
0.455647206
0.155797415
0.319425415
0.138963061
-0.208396752
0.125183567
-0.306554075
0.274368278
0.125646358
0.33372786
-0.248590928
0.310586051
0.21281434
-0.147988828
0.282041311
0.222026546
0.110661047
0.415148113
0.202297769
0.200791209
-0.306330687
0.160597817
0.114633495
0.119976792
0.185363312
0.170681332
0.478091143
0.41988641
0.211047725
-0.117471433
0.10164279
0.472246895
0.367673212
-0.116273582
0.186201566
0.223501474
0.127708918
0.517893516
0.241065974
-0.176148655
0.142990704

0.111603089
0.516834364
0.280114792
-0.177220431
0.183408967
-0.202337897
-0.148635979
0.27631314
0.127599198
0.126497608
0.300608687
0.133874011
0.112726081
-0.122702208
0.109021701
0.119002576
0.14836194
0.296526519
0.335091787
-0.200673649
-0.108383156
0.114419681
0.513038622
0.235888336
-0.103588653
0.433871714
0.10880147
0.118934563
0.134527498
0.370705249
0.143640149
0.192415565
0.172355041
-0.165600434
0.164280822
0.148004525
0.283437684
0.319638146
0.205993898
0.232429671
0.161861741
0.359754619
0.245275158
0.119500922
0.146202735
0.206259988
-0.104859984
-0.256308888
0.126350684
0.114763004
0.136944337
0.553972444
-0.169907272

0.17383766
0.104819496
0.101182857
0.338149842
0.334024708
0.135132135
0.232661285
0.244224277
0.399965387
0.160876604
0.280023127
0.443573692
0.444795911
0.191260257
0.337450486
0.303419628
0.217476774
0.117336351
-0.140410135
0.408336446
0.396188803
0.246628038
0.195828901
0.179241098
0.152491397
0.131754059
0.416111078
0.422056758
0.246558921
0.360667657
0.286866314
0.246679252
0.31155078
0.207154489
0.13801093
0.129839854
0.16084827
-0.203373055
-0.420490721
0.506979574
0.132422562
0.31973104
0.350619971
0.187903884
-0.20331133
0.356617509
0.407424042
0.463748417
0.199589262
0.221631412
0.353679328
0.33522862
0.17118467

0.157152516
-0.132450156
0.14509587
0.28620843
0.346552504
0.429780517
0.402788733
0.13734959
0.13719614
0.173951831
0.102782142
0.254581715
-0.369254293
0.118837382
0.176771875
0.17513593
0.158612136
0.355863368
0.306383029
0.122863659
-0.20643816
0.134766313
-0.143072808
-0.262450006
0.103491468
0.423268813
0.461183996
0.137045919
0.198621251
0.312629003
0.463396554
0.49694633
-0.110437525
0.157814558
0.145401909
0.194912172
0.126017692
0.127254144
0.140310674
0.100371681
0.125878547
0.150922822
-0.19183779
0.158717446
0.157871148
0.138495618
0.271114567
0.13205358
0.143758732
-0.119054817
-0.295333504
-0.106142841
0.145037821

0.146149023
0.222839272
0.116044262
0.142808416
0.190153024
0.116911585
0.125508903
0.10360226
0.114317622
0.229000202
0.211810747
0.175776502
0.532661883
0.291470327
0.28164552
0.146737441
0.3449461
0.123101416
0.205703888
0.189522444
0.267915153
0.279101092
0.117107245
0.487499614
0.433165423
0.112592311
0.123253576
0.37710448
0.134393362
0.312345319
0.406175545
-0.115652874
0.169285395
0.470141795
0.286580976
0.112687918
0.140949629
0.10796472
0.262241573
0.339211071
0.22477341
0.110819279
0.101344426
0.281406214
0.353873887
0.104801272
0.132974686
0.175601621
0.110405823
0.370501457
0.113699968
0.432048081
0.160530368

0.138747278
-0.281263671
0.103613457
0.368351744
0.164450055
-0.1799787
0.217533254
-0.148163678
0.146850534
0.176578514
0.392369115
0.207628108
0.261327885
0.434650503
0.259965161
0.349488218
0.113884679
-0.25148098
0.130662125
0.110621658
0.152710258
0.174199397
0.102787324
0.284333025
-0.104267772
0.102395394
0.276429326
-0.231677472
-0.164524875
-0.298772781
0.243464685
-0.123666194
0.171920643
0.140726241
0.523698908
-0.12646298
0.123615438
0.209162559
-0.262969267
0.196679476
0.36312202
0.212011366
0.1224459
0.156665062
0.290480483
0.225326389
0.443630155
-0.190821714
-0.148056252
0.200535458
0.234081852
0.109121037
0.449751724

0.27367489
0.162277854
0.208616093
0.161132666
0.12033196
0.116035709
0.401739408
0.448385906
0.151183092
0.422092411
0.374596523
0.471951772
0.452917017
-0.156205175
0.248268679
0.124543401
-0.275600967
0.182046869
0.109522676
0.118237516
-0.137836745
0.199440864
0.107853751
0.214077511
-0.23721732
0.281138715
0.256984302
-0.169592487
0.197741136
0.169118532
-0.157795996
0.189529651
0.251608321
0.172437502
0.242078204
-0.21838804
0.26069044
0.181577567
0.335419268
0.455555243
0.206663588
0.100914757
0.155981759
-0.106054004
0.134113414
0.108224851
0.345177723
0.161872049
0.129253823
0.303527094
0.206195561
0.469656379
0.287456363

0.12138344
0.312079757
0.241502623
0.181916374
0.500507065
0.16069
0.13032762
0.143551653
0.356923338
0.214615675
-0.107663023
-0.155050422
0.141817409
0.183597262
0.356671419
0.162887764
0.137911765
0.115531714
0.520006819
0.341499409
-0.185866415
0.224433149
0.237123769
0.183148071
0.428451484
0.346676211
0.310588412
0.141653953
0.244898952
0.319416958
0.154894253
0.202919588
0.501650707
0.461694838
0.171341876
0.495184547
0.345926223
0.297130103
0.231444478
0.365167383
-0.13958848
0.141655937
0.102746072
0.304742883
-0.249400433
0.104965114
0.352503315
0.19312195
0.293763891
0.26324621
0.330084401
-0.2425921
0.154531167

0.136443291
0.102073766
0.256000201
0.286167639
0.167994859
0.202226744
0.331137995
0.410657765
-0.325326733
0.336408561
0.124808109
0.17266977
0.193597407
-0.185279348
-0.16789542
0.105763249
0.322024991
0.170214295
0.143069504
0.165355605
0.129398451
0.524019627
0.381869318
0.201797536
0.128985245
0.113167363
0.110405703
0.523579721
0.219455414
0.161242922
0.333226225
0.375309143
0.155757874
0.266545107
0.534716456
0.600106048
0.210712477
-0.16253373
-0.157011728
-0.1379578
0.257894944
-0.197928266
-0.148104747
0.39656196
0.156478754
0.514074545
0.211816133
0.534752865
0.19168906
-0.100292022
0.17701415
-0.24408957
0.132277329

0.161019494
0.356864453
0.370698944
0.456335467
0.220005905
0.36967467
0.18884389
0.156572137
0.266706352
0.121193334
0.153922975
0.259551793
0.131488939
0.264146547
0.146748202
0.262414627
0.127101311
-0.19861785
0.162639919
0.128980119
-0.128959111
0.127825091
0.320945004
0.395341516
0.20209886
0.290402642
-0.109544934
-0.104309119
-0.123270087
0.186632542
-0.215776205
0.103442147
0.272945892
0.131793615
-0.216477772
-0.141935032
0.121884079
0.102771188
0.241200992
0.310188084
0.357176093
0.334661436
0.247929731
0.117652091
0.111364696
0.126931011
0.166441677
0.267605229
-0.187454232
0.114001008
0.140190804
0.180034158
0.151423536

0.344112124
0.183818369
0.256958824
-0.116676927
0.126977322
0.109218111
0.113105925
0.313902095
0.125646295
0.152598941
0.214704059
0.313014946
0.200672081
0.144409655
0.153411836
0.129166493
0.173783858
0.134614
0.107453355
0.207575342
0.138281911
0.515069689
0.506731418
0.139945553
0.111108762
0.158680055
-0.125482114
0.395099088
0.21217417
0.169452097
0.102353366
0.100819064
0.150908817
0.11656937
0.17446865
-0.129322495
0.111203017
0.193578905
0.158546562
0.173594177
0.292687355
0.126257921
0.359488983
0.127362676
-0.137437421
0.177592865
0.190754152
0.167708499
-0.180436654
0.248926803
-0.14592685
0.239188591
0.140911146

0.426215842
0.142962101
0.226266534
0.267777027
0.121464431
0.141680152
0.135017973
0.19475145
0.297784049
0.211747002
0.156603671
-0.176208674
-0.13922749
0.250739481
-0.10097922
0.120157045
0.323625057
0.300587228
0.226140159
-0.271507873
-0.197931141
0.324521787
0.281665468
-0.128696133
0.11297271
0.154728753
0.112430359
0.14462892
0.190732473
0.130072217
0.202238329
-0.167548927
-0.185205513
0.177115419
0.213081665
0.102817439
0.103540423
0.208880994
0.152831794
0.166135016
0.151267242
0.517162822
0.138187336
0.110097417
0.142073629
-0.197383253
0.139862524
-0.177692267
-0.18069523
0.189634889
0.124057807
0.318095528
0.194553887

0.172063404
0.144899708
-0.167546899
-0.187506601
0.322801093
0.159370984
0.171807987
0.163063819
0.150477484
0.304922384
0.224516439
-0.160960174
0.158996169
0.117940674
0.320881578
-0.102331657
0.26597582
-0.242105354
0.212712521
0.152190014
-0.210351416
0.161939806
0.297874318
0.112037912
0.249010337
-0.185412586
0.348647426
-0.35072721
0.283226741
0.278451138
0.184925942
0.101122687
0.166273884
-0.265109278
0.271801218
0.466967192
0.454659262
0.387518065
0.334246782
0.206005997
-0.136771482
-0.224764002
0.231698611
0.210390698
0.100905554
-0.176411912
0.230458517
0.310800108
-0.153238056
0.39431389
0.297173564
-0.100025325
-0.451597702

0.268268959
-0.199044505
0.105235938
-0.102812469
0.204577866
0.218477279
0.252670023
0.504355559
-0.2022284
